# Saccharide success: exploring the role of d-fructose-based thioureas as organocatalysts for the enantioselective Friedel–Crafts alkylation reaction†

**DOI:** 10.1039/d5ra00456j

**Published:** 2025-03-18

**Authors:** Samson Lalhmangaihzuala, Vanlalngaihawma Khiangte, Khiangte Vanlaldinpuia

**Affiliations:** a Department of Chemistry, Pachhunga University College, Mizoram University Aizawl 796001 Mizoram India mapuiakhiangte@gmail.com; b Department of Chemistry, Mizoram University Tanhril Aizawl 796004 Mizoram India

## Abstract

In this study, a series of sixteen (16) d-fructose-based bifunctional thioureas were examined as organocatalysts for the enantioselective Friedel–Crafts alkylation of indoles and pyrrole with β-nitrostyrenes. This investigation is a part of our ongoing project, which aims to expand the scope of application of d-fructose-based thioureas. Under the optimized low-temperature reaction conditions, the corresponding adducts were obtained with good yield (up to 95%) and excellent enantioselectivity (>99% ee).

## Abstract 2

In this study, a series of sixteen (16) d-fructose-based bifunctional thioureas were examined as organocatalysts for the enantioselective Friedel–Crafts alkylation of indoles and pyrrole with β-nitrostyrenes.

## Graphical abstract

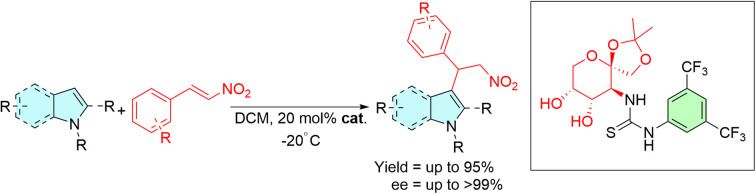

## 1. Introduction

Chiral indolyl compounds showcase a diverse range of applications and play a significant role in drug discovery, agrochemicals and functional materials.^1,2^ Therefore, the development of efficient synthetic methodologies to directly functionalize indoles and their derivatives bears considerable importance. The asymmetric Friedel–Crafts alkylation of indoles represents one of the most effective approaches for obtaining optically active indoles with high enantioselectivity.^1,3,4^ A plethora of enantiomerically enriched indoles were successfully prepared using several electrophilic species, including ketoesters, epoxides, imines, enamines, and nitroolefins, as well as unsaturated compounds including aldehydes, ketones, ketophosphonates and imidazoles.^5,6^ In recent years, a simple yet highly efficient enantioselective alkylation of indoles using chiral organocatalysts and metal complexes has been developed.^5,7–15^ However, the reported methods have several drawbacks such as limited substrate scope, low yields and selectivities, use of expensive reagents and/or catalysts and limited scalability, among others. Hence, the development of a new catalytic system and improved reactions condition that address these challenges is still essential for the efficient and sustainable synthesis of enantioenriched indolyl compounds.

Since the year 2000, the application of chiral organocatalysts has significantly transformed various asymmetric processes, leading to a dramatic expansion of research activities in academia.^16,17^ After the seminal introduction made by Kunz research groups on saccharide-derived thiourea promoted enantioselective Strecker and Mannich reactions,^18^ the utilization of chiral bifunctional thiourea organocatalysts bearing carbohydrate scaffolds has become a powerful tool for the synthesis of relevant non-racemic compounds. Carbohydrates are highly prevalent biomolecules that possess distinctive characteristics such as high optical purity, structural diversity, a high degree of functionality and well-defined stereocenters.^19–23^ These qualities make them readily manipulable for the development of valuable synthetic molecules such as chiral auxiliaries, reagents, and catalysts.^24–26^

**Scheme 1:**
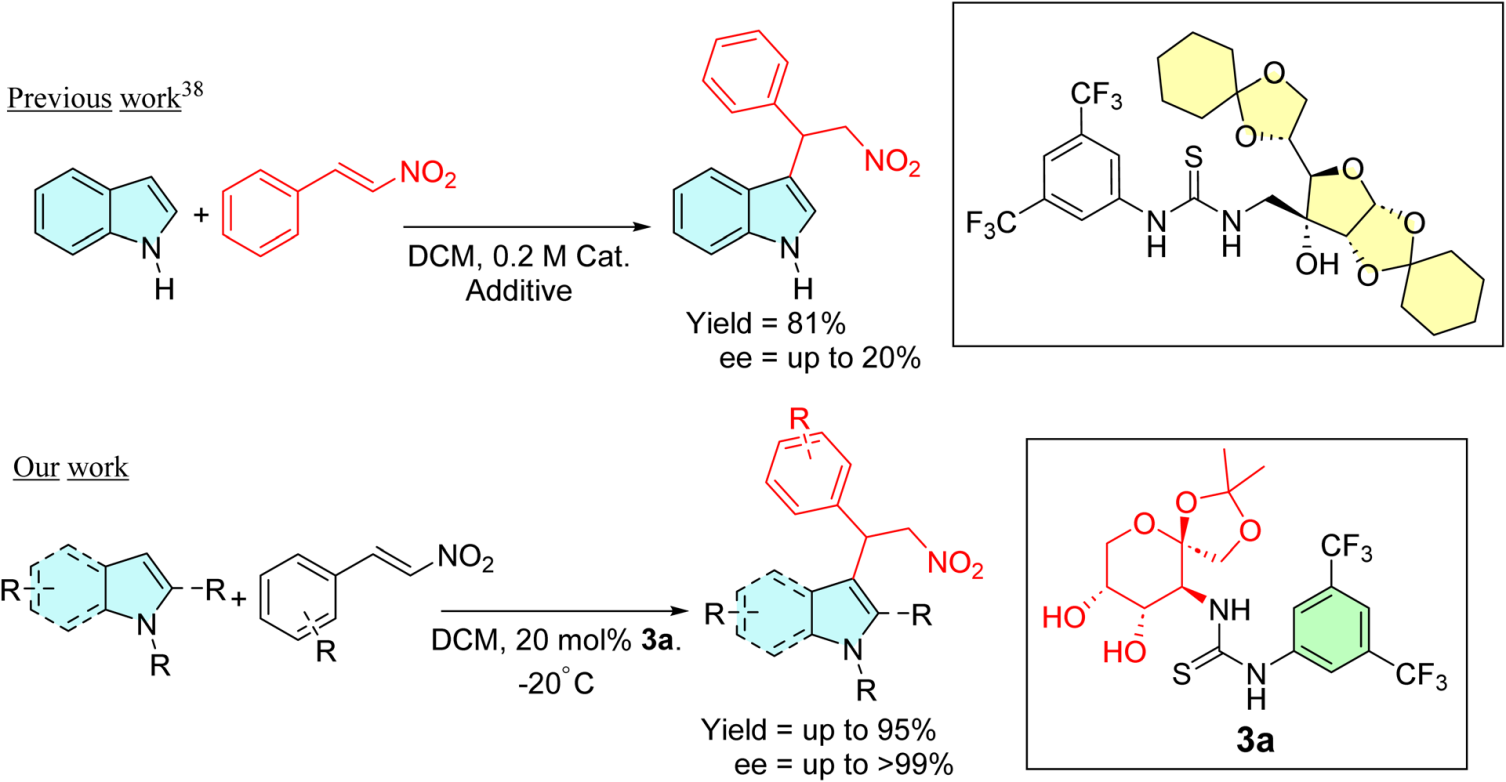
Comparison of previous work conducted by Turks *et al.* with our present work.

Various asymmetric organic transformations, including but not limited to the Michael addition, aldol reactions, Morita–Baylis–Hillman reactions, and aza-Henry reactions, have since been successfully accomplished using carbohydrate-based thioureas as catalysts.^27–40^ However, to the best of our knowledge, there is only one example that illustrates saccharide-derived thiourea-catalyzed enantioselective addition of indoles to β-nitrostyrenes. Unfortunately, this report described that although a high yield of the alkylated adducts was obtained, the enantiomeric excess was poor, reaching a maximum of only 20% ee (Scheme 1).^41^ In light of our interest in the fields^42–45^ and our goal of establishing an improved reaction condition, we have decided to investigate the catalytic activities of d-fructose-based thioureas in the asymmetric Friedel–Crafts alkylation reactions.

## 2. Result and discussion

**Fig. 1:**
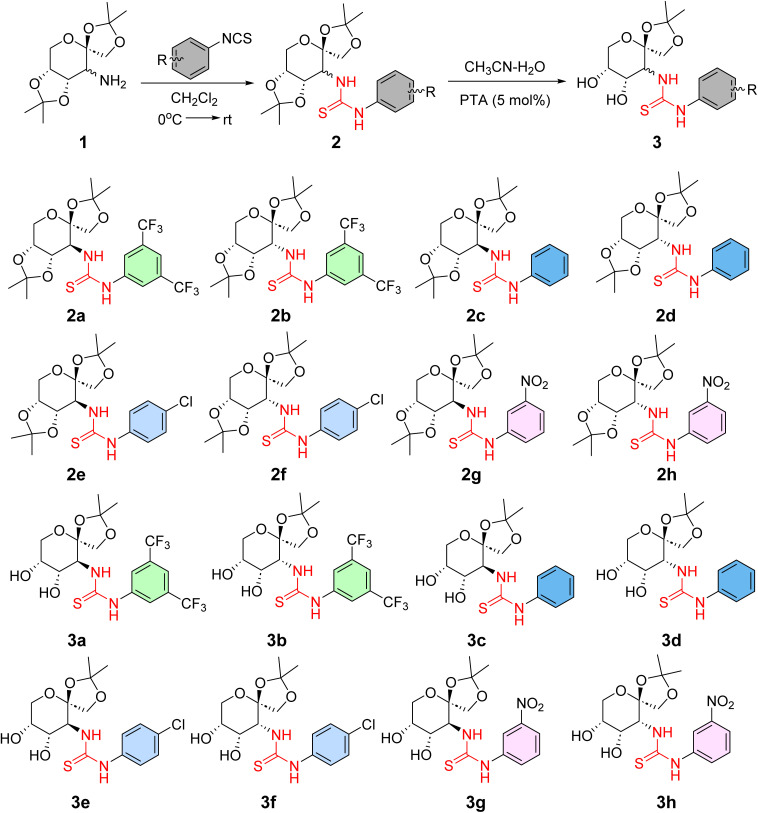
d-Fructose-derived thioureas^46^ screened for the Friedel–Crafts alkylation reaction.

Initially, a collection of bifunctional saccharide-based thiourea catalysts 2a–2h and 3a–3h (Fig. 1) was prepared according to our previously reported literature,^46^ and their catalytic performance to promote the Friedel–Crafts addition of various indoles and pyrrole to nitroalkenes was assessed. The alkylation of indole using *trans*-4-chloro-β-nitrostyrene was selected as a model reaction for screening of the catalyst and other reaction parameters. The results obtained are presented in Table 1.

**Table 1:** Optimization of reaction condition in the presence of thiourea organocatalyst^a^

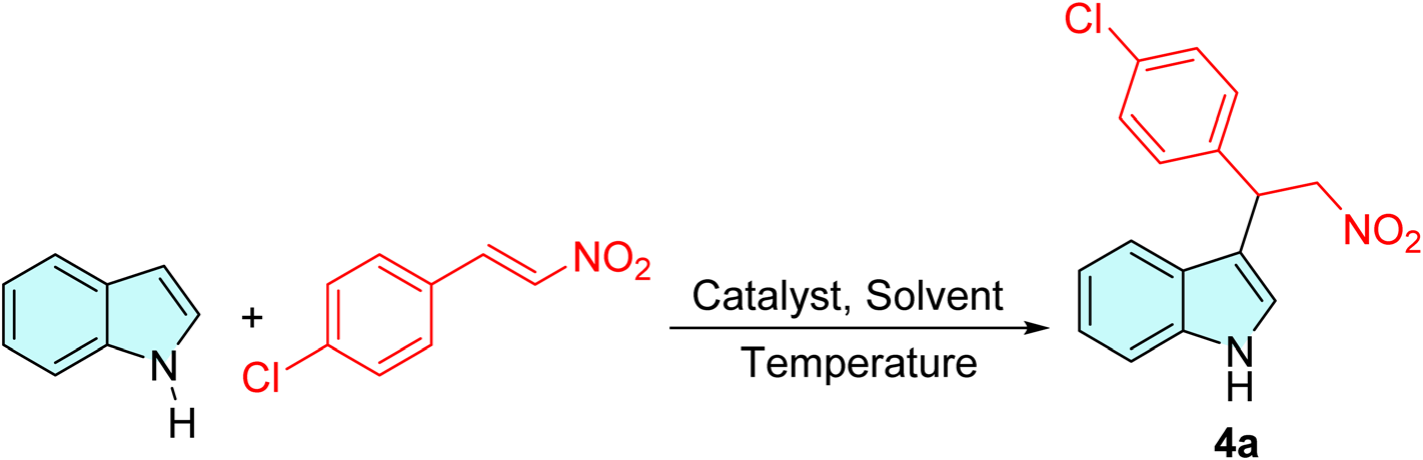						
Entry	Catalyst (in mol%)	Temperature (°C)	Time (h)	Solvent	Yield^b^ (%)	ee^c^ (%)
1	2a–2h (20)	RT	96	Toluene	Trace	n.d^d^
2	3a (20)	RT	94	Toluene	87	9
3	3b (20)	RT	92	Toluene	88	4
4	3c (20)	RT	120	Toluene	60	*rac*
5	3d (20)	RT	128	Toluene	57	*rac*
6	3e (20)	RT	96	Toluene	63	*rac*
7	3f (20)	RT	72	Toluene	69	*rac*
8	3g (20)	RT	78	Toluene	72	*rac*
9	3h (20)	RT	88	Toluene	77	*rac*
10	3a (20)	RT	72	DCM	85	13
11	3b (20)	RT	76	DCM	82	3
12	3a (20)	RT	144	CH_3_CN	80	8
13	3b (20)	RT	126	CH_3_CN	82	*rac*
14	3a (20)	RT	168	EtOH	25	6
15	3a (20)	RT	120	H_2_O	Nr^d^	Nd^e^
16	3a (20)	RT	72	Neat	Nr^d^	Nd^e^
17	3a (20)	−10	126	DCM	84	67
18	3a (20)	−20	144	DCM	83	97
19	3b (20)	−20	128	DCM	85	76
20	3a (20)	−20	136	Toluene	84	58
21	3b (20)	−20	144	Toluene	81	42
22	3a (10)	−20	152	DCM	51	73
23	3a (15)	−20	163	DCM	74	89

a Reaction condition: to a solution of thiourea catalyst in 2 ml solvent was added 0.5 mmol of indole and 0.6 mmol of *trans*-4-Cl-β-nitrostyrene.

b Yield of the desired product.

c The enantiomeric excess was determined by High Performance Liquid Chromatography (HPLC).

d No reaction.

e Not determined.

As given in Table 1, the initial reactions performed using the saccharide-based thioureas 2a–2h to produce the desired adduct were sluggish and afforded only a trace amount of the products (Table 1, entry 1). However, it was observed that employing thioureas 3a–3h, obtained from the deprotection of the 4,5-*O*-isopropylidene ring of thioureas 2a–2h was capable of promoting the reaction to afford the products in good to excellent yields at room temperature, albeit with a near-racemic or racemic mixture (Table 1, entry 2–9). This result demonstrated that the presence of free hydroxy groups at the saccharide scaffold made a significant contribution to the progress of this reaction. Amongst the variants of bifunctional compounds investigated (Table 1, entries 2–9), thioureas 3a and 3b bearing 3,5-bis(trifluoromethyl)phenyl moiety afforded the best result: 87% yield and 9% enantioselectivity for the former, while achieving 88% chemical yield and 4% ee for the latter. The outcomes obtained prompted us to select and explore the catalytic activity of the thiourea catalysts 3a and 3b at different reaction conditions. Subsequently, as shown in Table 1, entries 10–15, an additional survey on the influence of different solvents was conducted. A slight improvement in the enantioselectivity was detected without much affecting the yield of the reaction when DCM was used as a solvent (Table 1, entries 10 and 11). However, we observed a decrease in the percentage of yield and ee when acetonitrile and ethanol were employed. And the reaction did not proceed at all under neat condition or when water was employed as a solvent (Table 1, entries 15 and 16). This can be explained by the nature of thiourea-based organocatalysts and their interaction with protic solvents such as water and ethanol. These solvents can disrupt the catalyst's function by competing with the substrate molecule for H-bonding sites of the chiral catalyst.^47^ This competition hinders the catalyst's ability to activate the electrophile, thus impeding the desired reaction.

We further optimized the reaction temperature to enhance the enantiomeric excess of the alkylated adducts. Remarkably, the reaction temperature was found to play a crucial role in determining the stereochemical result of the product. As indicated in Table 1, entry 17, the product 4a was obtained in 67% ee at a reaction temperature of −10 °C. When the reaction temperature was further reduced to −20 °C, significant improvement in the enantioselectivity of the reaction was observed, achieving 96% ee and 83% yield of the adduct (Table 1, entry 18). On the contrary, its diastereomeric congener 3b fell short of our expectations, yielding the corresponding product in excellent yield but with poor enantioselectivity (Table 1, entry 19). Additional investigation of toluene as a reaction medium at lower temperatures gave poor enantiomeric excess values (Table 1, entries 20 and 21). Hence, DCM was selected as the optimum solvent for the reaction. As shown in Table 1, entries 22 and 23, the catalyst's ability to maintain the stereocontrolled reaction condition diminished with the reduction of the catalyst loading, resulting in a decrease in the enantioselectivity of the product.

After establishing the optimized reaction condition (Table 1, entry 18), we next examined the asymmetric Friedel–Crafts alkylation of several indoles to nitroalkenes for the synthesis of optically active indolyl compounds and the results obtained are presented in Table 2. The reaction of indole with a range of aromatic nitrostyrenes bearing electron-neutral, electron-deficient or electron-withdrawing groups was first investigated (Table 2, entries 1–7). The substituted nitroalkenes reacted efficiently to afford the desired adducts in good yield (up to 95%) and excellent enantioselectivity (82–97% ee). The results demonstrated that both the yield and enantioselectivity were not much influenced by the location and electronic nature of the substituents at the aromatic nucleus. When 2-chloro-substituted nitrostyrene was employed as an acceptor, a slightly lowered enantioselectivity was obtained (Table 2, entry 4). In continuation, a variety of electron-rich indoles were also investigated (Table 2, entries 8–17). The reaction of nitrostyrenes with 2-methyl and 1-methyl *N*-substituted indoles was able to afford the alkylated products 4h–4m in moderate to practically enantiopure form, albeit with a lowered reaction yield (Table 2, entries 8–13). There was a significant improvement in the enantiomeric excess when the substituent was placed at the *ortho*- and *para*-positions of the aromatic ring (Table 2, entries 9 and 10). However, the product obtained from the substrate 1-methyl indole showed decreased enantioselectivity (Table 2, entries 11–13). Additionally, other heteroaromatic electron-rich indoles (Table 2, entries 14 and 15) were also amenable to this asymmetric reaction and gave rise to the corresponding products 4m and 4o. Finally, we have also investigated the alkylation reactions of 5-nitro substituted indoles, and surprisingly, the catalyst was found to be inactive and no traces of formation of the corresponding adduct were observed (Table 2, entries 16 and 17). It is believed that the presence of a strong electron-withdrawing nitro group significantly reduces the nucleophilicity of the indole moiety, which may have inhibited the progress of the reaction.

**Table 2:** Enantioselective asymmetric reaction of indoles with aromatic nitrostyrene^a^

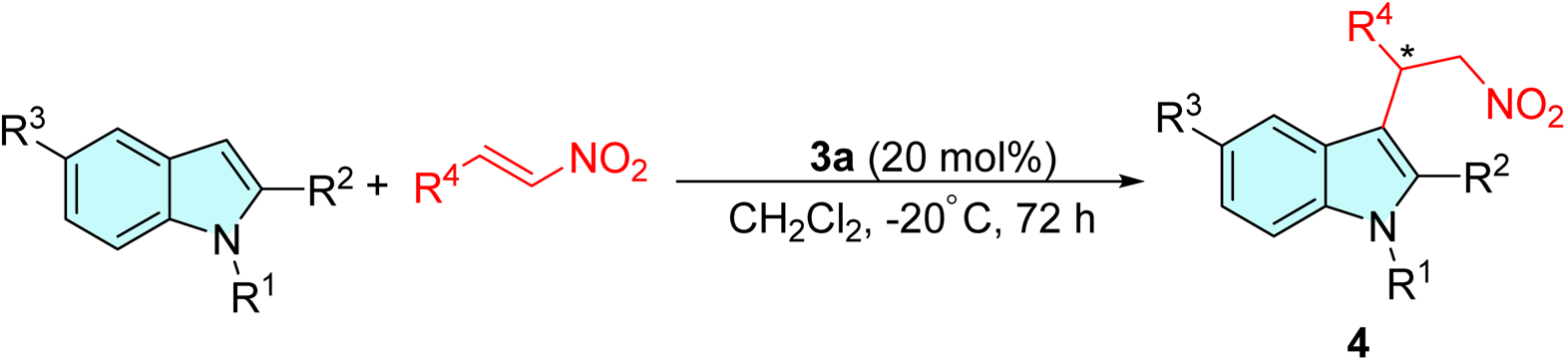								
Entry	*R* ^1^	*R* ^2^	*R* ^3^	*R* ^4^	Time (h)	Product	Yield^b^ (%)	ee^c^ (%)
1	H	H	H	4-ClPh	144	4a	83	97
2	H	H	H	4-BrPh	130	4b	75	91
3	H	H	H	4-MePh	152	4c	64	95
4	H	H	H	2-ClPh	128	4d	74	82
5	H	H	H	2-OHPh	120	4e	94	96
6	H	H	H	2-OMePh	128	4f	95	91
7	H	H	H	Ph	144	4g	78	90
8	H	Me	H	Ph	136	4h	52	84
9	H	Me	H	4-BrPh	128	4i	57	>99
10	H	Me	H	2-ClPh	120	4j	55	>99
11	Me	H	H	Ph	144	4k	46	66
12	Me	H	H	4-BrPh	168	4l	61	82
13	Me	H	H	4-MePh	152	4m	65	95
14	Bn	H	H	Ph	162	4n	52	75
15	H	H	Me	Ph	144	4o	46	82
16	H	H	NO_2_	Ph	168	4p	Nr^d^	Nd^e^
17	Me	H	NO_2_	Ph	168	4q	<3^f^	Nd^e^

a To a solution of 20 mol% of catalyst 3a in 2 ml DCM was added 0.5 mmol of β-nitrostyrenes and 0.6 mmol of indoles.

b Isolated yield.

c The ee values are determined by HPLC using chiral column. The absolute configuration of the major isomer of the corresponding Friedel–Crafts adducts was assigned as *R* by comparison with literature.

d No reaction.

e Not determined.

f Progress of the reaction determined using thin layer chromatography (TLC).

**Fig. 2:**
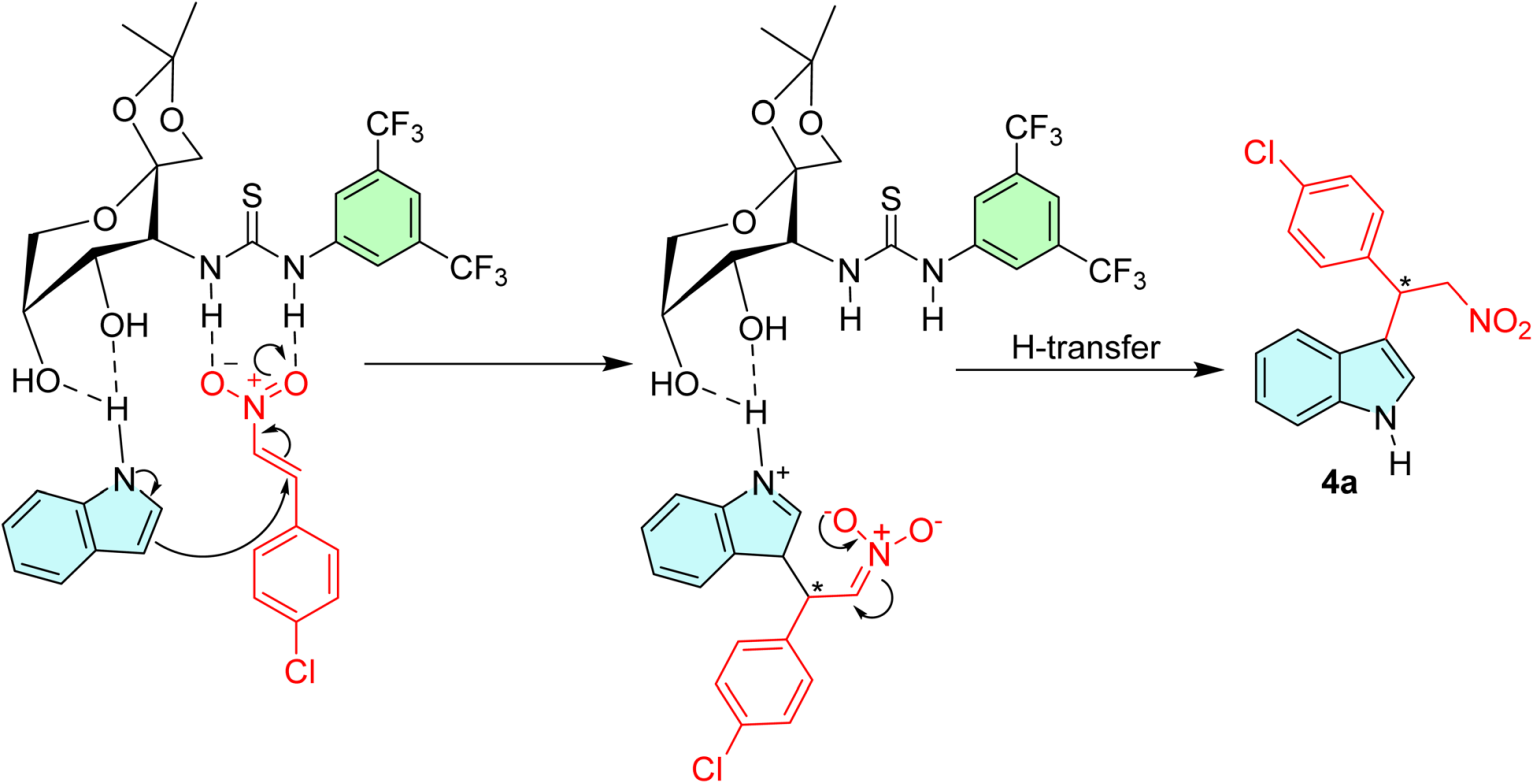
Proposed reaction mechanism.

Based on the observed experimental results and existing literature reports,^7,39^ we assumed that the catalyst 3a operates in a bifunctional manner when interacting with the substrate molecules. As shown in Fig. 2, a plausible reaction mechanism for the alkylation of indole with *trans*-4-Cl-β-nitrostyrene was proposed. The presence of a 3,5-bis(trifluoromethyl)phenyl skeleton has significantly enhanced the double-H bonding interaction between the hydrogen atoms of the thioureas and the nitro group. This interaction stabilizes the position of the β-nitrostyrenes and increases their electrophilic nature. In the meantime, the two hydroxy groups attached to the saccharide scaffold activate the indole moiety *via* H-bond formation. Subsequently, this activation then initiates an attack on the electron-deficient nitrostyrene to generate the corresponding adducts. However, it has been observed from Table 2, entries 11–14, that the reaction proceeded effortlessly with *N*-substituted indoles as well, without substantial variations in yields or enantioselectivity. This observation suggests an alternative mechanistic pathway, distinct from the typical N–H hydrogen bond activation. Herein, the hydroxy groups on the saccharide framework was presumed to engage with the indole moiety through the OH⋯π (indole) interaction, facilitating the subsequent nucleophilic attack on the nitrostyrenes.^48^

Further investigation of the synthetic potential of this sugar-based thiourea catalytic system was successfully conducted on electron-rich pyrrole as demonstrated in Table 3. The enantioselective alkylation of pyrrole with β-nitrostyrenes was able to obtain the corresponding alkylated adducts 5a–5d with moderate yield and good enantioselectivities.

**Table 3:** Enantioselective asymmetric reaction of pyrroles to nitrostyrene^a^

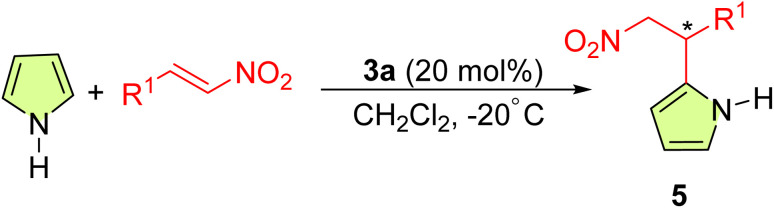					
Entry	*R* ^1^	Time (h)	Product	Yield^b^ (%)	ee^c^ (%)
1	Ph	168	5a	41	87
3	2-ClPh	96	5b	52	80
3	4-ClPh	156	5c	45	82
4	4-MePh	168	5d	44	93

a To a solution of 20 mol% of catalyst 3a in 2 ml DCM was added 0.5 mmol of β-nitrostyrenes, 0.75 mmol of pyrrole.

b Isolated yield.

c The ee values are determined by HPLC using chiral column.

## 3. Conclusion

Our study presents a successful application of bifunctional thiourea organocatalysts having d-fructose as the core chiral scaffold for the asymmetric Friedel–Crafts reaction. In the current finding, thiourea 3a worked effectively to promote an enantioselective addition of indoles and pyrrole to β-nitrostyrenes. The examination of both reaction mediums and temperature has highlighted the fundamental importance of their existence for enhancing the stereochemical results. Notably, the reaction proceeded efficiently without the need for any additional additives. Under the optimized reaction environment, the catalyst was able to afford the corresponding alkylated product with excellent yield (up to 95%) and enantioselectivity (>99%).

## 4. Experimental

### 4.1. General

Chemicals were purchased from Sigma Aldrich and HiMedia and used as received unless otherwise stated. Bruker Avance II, 400 and 500 Mhz was used for recording ^1^H NMR and ^13^C NMR spectra. CDCl_3_ was used as a solvent and chemical shifts are calculated in *δ* with tetramethylsilane (Me_4_Si) as reference. The enantiomeric excess of the products was analyzed with Waters 1525 binary pump and Waters UV detector 2489 using Chiralpak OD-H or AD-H column with *n*-hexane and iso-propanol as an eluent. Optical rotations were determined using a Rudolph Research Analytical Polarimeter with dichloromethane and methanol as the solvent. Measurements are reported as follows: [*α*]^25^_D_ (concentration in mg per 10 ml, solvent).

### 4.2. Preparation of carbohydrates derived thioureas 2a–2h (ref. 46)

A solution of 1 mmol of the corresponding isothiocyanate in 2 ml of dry dichloromethane (DCM) was mixed with 1 mmol (0.258 mg) of sugar amines 1 (prepared according to literature^42^) at 0 °C. Then, the reaction mixture was permitted to reach room temperature and was stirred for another 48 h. The mixture was then extracted using ethyl acetate and washed with water (2×). The organic layer was dried over Na_2_SO_4_, filtered, evaporated and the pure product was obtained by column chromatography using 20% ethyl acetate in hexane as an eluent.

### 4.3. Synthesis of carbohydrates derived thioureas 3a–3h (ref. 46)

To a 100 ml round bottom flask charged with 5 mol% of phosphotungstic acid (144 mg) and 1 mmol of the corresponding thiourea compound (2a–2h), 5 ml of CH_3_CN : H_2_O (9 : 1 ratio) mixture was added.^44^ The reaction mixture was allowed to be stirred at room temperature for 6 h. After the reaction was completed, the solvents were evaporated under reduced pressure, and the mixture was diluted with ethyl acetate (EtOAc) and washed with water (3×). The organic layer was separated and dried using anhydrous Na_2_SO_4_. After filtration, the organic layer was concentrated to give the crude product which was further purified by column chromatography using 40% ethyl acetate in hexane as an eluent and silica gel (60–120 mesh) as a stationary phase to obtain the pure product. The partial deisopropylidenation of 2a–2h to afford 3a–3h could also be performed according to the procedure reported by Shi and co-workers.^49^ The thiourea derivative (2a–2h, 1 mmol) was dissolved in 5 ml of CH_3_CN : H_2_O (9 : 1) mixture, and 10 mol% (22 mg) of 2,3-dichloro-5,6-dicyano-1,4-benzoquinone (DDQ) was added. The reaction was completed after stirring the mixture at room temperature for 8 h. The solvent evaporated, and the residue obtained was dissolved in EtOAc, washed with water (2×) and dried over anhydrous Na_2_SO_4_. The crude obtained was purified by column chromatography using 40% EtOAc in hexane as an eluent.

#### 4.3.1. 1,2;4,5-Di-*O*-isopropylidene-3-[3,5-bis(trifluoromethyl)phenylthioureido]-3-deoxy-β-d-fructopyranose (2a)

Yield%: 96% as white solid; mp: 157–159 °C. [*α*]^25^_D_ −143.50° (*c* 0.001, CHCl_3_). ^1^H NMR (400 MHz, CDCl_3_): *δ* 8.78 (s, 1H), 8.046 (s, 1H), 7.95 (s, 1H), 7.67 (s, 1H), 6.36 (s, 1H), 4.32–3.97 (m, 7H), 1.63–1.36 (m, 12H) ppm. ^13^C{^1^H} NMR (100 MHz, CDCl_3_): *δ* 182.3, 139.0, 132.7, 132.4, 124.5, 124.2, 123.3, 121.4, 119.3, 112.2, 111.3, 110.1, 105.0, 77.9, 75.2, 60.1, 58.1, 29.6, 29.5, 28.1, 27.9 ppm. IR (KBr): 3321.49, 2989.56, 2936.91, 1529.73, 1468.40, 1381.53, 1278.74, 1222.07, 1177.62, 1136.09, 1079.50, 969.78, 885.92, 762.27 cm^−1^. ESI-MS (*m*/*z*): 531.5 (M^+^). Elemental analysis for C_21_H_24_F_6_N_2_O_5_S: C 47.55, H 4.56, F 21.49, N 5.28, O 15.08, S 6.02: found C 47.35, H 4.86, F 21.22, N 5.68, O 14.68, S 6.20.

#### 4.3.2. 1,2;4,5-Di-*O*-isopropylidene-3-[3,5-bis(trifluoromethyl)phenylthioureido]-3-deoxy-α-d-fructopyranose (2b)

Yield: 80% as white solid; mp: 150–152 °C. [*α*]^25^_D_ −114.63° (*c* 0.001, CHCl_3_). ^1^H NMR (400 MHz, CDCl_3_): *δ* 8.63 (s, 1H), 7.95 (s, 2H), 7.68 (s, 1H), 6.70 (d, ^3^*J* = 8.8 Hz, 1H), 4.64–3.93 (m, 7H), 1.72 (s, 3H), 1.51 (s, 3H), 1.42 (s, 3H), 1.33 (s, 3H) ppm. ^13^C{^1^H} NMR (100 MHz, CDCl_3_): *δ* 180.6, 138.7, 132.3, 124.5, 124.5, 123.3, 121.7, 119.1, 111.5, 104.9, 72.9, 72.3, 62.2, 55.7, 26.6, 26.1, 25.9, 24.9 ppm. IR (KBr): 3333.10, 2933.83, 1535.59, 1466.80, 1382.22, 1278.33, 1135.27, 1065.27, 878.77, 757.44 cm^−1^. ESI-MS (*m*/*z*): 531.5 (M^+^). Elemental analysis for C_21_H_24_F_6_N_2_O_5_S: C 47.33, H 4.48, F 22.09, N 5.23, O 15.02, S 5.84: found C 47.45, H 4.20, F 21.98, N 5.02, O 15.34, S 5.99.

#### 4.3.3. 1,2;4,5-Di-*O*-isopropylidene-3-(phenylthioureido)-3-deoxy-β-d-fructopyranose (2c)

Yield: 91% as white solid; mp: 162–165 °C. [*α*]^25^_D_ −176.83° (*c* 0.001, CHCl_3_). ^1^H NMR (500 MHz, CDCl_3_): *δ* 8.50 (s, 1H), 7.40–7.26 (m, 5H), 6.00 (s, 1H), 4.15–3.93 (m, 7H), 1.66 (s, 3H), 1.38 (s, 3H), 1.32 (s, 3H), 1.06 (s, 3H) ppm. ^13^C{^1^H} NMR (125 MHz, CDCl_3_): *δ* 181.6, 135.6, 130.1, 127.5, 125.8, 111.0, 109.7, 105.0, 75.4, 72.5, 72.1, 60.3, 56.6, 27.8, 26.4, 25.9, 25.4 ppm. IR (KBr): 3378, 3291, 3204, 2932, 2291, 1510, 1358, 1208, 1062, 873, 747 cm^−1^. ESI-MS (*m*/*z*): 395.1 (M^+^). Elemental analysis for C_19_H_26_N_2_O_5_S: calculated C57.85, H 6.64, N 7.10, O 20.28, S 8.13; found C 57.66, H 6.46, N 7.35, O 20.42, S 8.11.

#### 4.3.4. 1,2;4,5-Di-*O*-isopropylidene-3-(phenylthioureido)-3-deoxy-α-d-fructopyranose (2d)

Yield: 81% as white solid; mp: 92–95 °C. [*α*]^25^_D_ −142.00° (*c* 0.002, CHCl_3_). ^1^H NMR (400 MHz, CDCl_3_): *δ* 8.46 (s, 1H), 7.45–7.28 (m, 5H), 6.57 (d, 1H, ^3^*J* = 9.2 Hz), 4.61–3.86 (m, 7H), 1.46 (s, 3H), 1.40 (s, 3H), 1.31 (s, 3H), 1.26 (s, 3H) ppm. ^13^C{^1^H} NMR (100 MHz, CDCl_3_): *δ* 180.6, 135.9, 130.0, 129.1, 126.9, 124.3, 111.0, 109.3, 104.5, 72.8, 72.1, 71.7, 61.9, 55.5, 26.5, 25.8, 25.6, 24.7 ppm. IR (KBr): 3370.75, 2987.64, 2934.76, 1534.88, 1379.70, 1310.62, 1212.07, 1105.99, 1064.75, 872.31, 755.08 cm^−1^. ESI-MS (*m*/*z*): 395.5 (M^+^). Elemental analysis for C_19_H_26_N_2_O_5_S: calculated C 57.72, H 6.32, N 7.21, O 19.89, S 8.85; found C 57.52, H 6.52, N 7.13, O 20.10, S 8.72.

#### 4.3.5. 1,2:4,5-Di-*O*-isopropylidene-3-(4-chlorophenylthioureido)-3-deoxy-β-d-fructopyranose (2e)

Yield: 87% as white solid; mp: 165–167 °C. [*α*]^25^_D_ −146.83° (*c* 0.002, CHCl_3_). ^1^H NMR (400 MHz, CDCl_3_): *δ* 7.77 (s, 1H), 7.40–7.24 (m, 4H), 5.87 (s, 1H), 4.19–3.97 (m, 7H), 1.69 (s, 3H), 1.58 (s, 3H), 1.43 (s, 3H), 1.37 (s, 3H) ppm. ^13^C{^1^H} NMR (100 MHz, CDCl_3_): *δ* 182.3, 136.0, 132.7, 131.8, 130.6, 128.4, 127.9, 125.8, 110.2, 109.4, 105.6, 72.8, 72.5, 60.6, 57.3, 29.9, 28.1, 26.6, 25.6 ppm. IR (KBr): 3367.43, 2933.39, 1530.50, 1378.17, 1224.43, 1081.58, 882.02, 758.40, 521.71 cm^−1^. ESI-MS (*m*/*z*): 429.5 (M^+^). Elemental analysis for C_19_H_25_ClN_2_O_5_S: calculated C 53.20, H 5.88, Cl 8.26, N 6.53, O 18.65, S 7.47; found C 53.32, H 5.64, Cl 8.33, N 6.32, O 18.53, S 7.84.

#### 4.3.6. 1,2:4,5-Di-*O*-isopropylidene-3-(4-chlorophenylthioureido)-3-deoxy-α-d-fructopyranose (2f)

Yield: 76% as white solid; mp: 169–171 °C. [*α*]^25^_D_ −106.50° (*c* 0.001, CHCl_3_). ^1^H NMR (400 MHz, CDCl_3_): *δ* 8.89 (s, 1H), 7.38–7.28 (m, 4H), 6.58 (d, 1H, ^3^*J* = 9.2 Hz), 4.60–3.88 (m, 7H), 1.46–1.24 (m, 12H) ppm. ^13^C{^1^H} NMR (100 MHz, CDCl_3_): δ180.6, 135.0, 132.0, 129.8, 128.9, 128.1, 125.5, 111.0, 109.3, 104.5, 76.8, 72.7, 72.1, 61.8, 60.3, 55.3, 26.4, 25.8, 25.5, 24.7 ppm. IR (KBr): 3362.19, 2932.37, 1530.82, 1379.35, 1225.11, 1081.54, 882.21, 756.84 cm^−1^. ESI-MS (*m*/*z*): 429.4 (M^+^). Elemental analysis for C_19_H_25_ClN_2_O_5_S: calculated C 53.32, H 5.61, Cl 8.11, N 6.83, O 18.28, S 7.84; found C 53.46, H 5.45, Cl 8.15, N 6.51, O 18.33, S 8.1.

#### 4.3.7. 1,2:4,5-Di-*O*-isopropylidene-3-(3-nitrophenylthioureido)-3-deoxy-β-d-fructopyranose (2g)

Yield: 81% as yellow solid; mp: 93–95 °C. [*α*]^25^_D_ −209.50° (*c* 0.001, CHCl_3_). ^1^H NMR (400 MHz, DMSO-d_6_): *δ* 10.12 (s, 1H), 8.79 (s, 1H), 7.98–7.60 (m, 4H), 4.31–3.81 (m, 7H), 1.51 (s, 3H), 1.48 (s, 3H), 1.44 (s, 3H), 1.41 (s, 3H) ppm. ^13^C{^1^H} NMR (100 MHz, DMSO-d_6_): *δ* 182.5, 148.1, 132.0, 130.3, 129.0, 117.1, 111.5, 109.7, 75.9, 74.7, 60.5, 60.4, 60.3, 27.1, 26.8, 26.6, 26.4 ppm. IR (KBr): 3367.63, 2928.16, 1529.95, 1381.3, 1221.20, 1079.57, 770.4 cm^−1^. ESI-MS (*m*/*z*): 440.5 (M^+^). Elemental analysis for C_19_H_25_N_3_O_7_S: calculated C 51.93, H 5.73, N 9.56, O 25.48, S 7.29; found C 51.88, H 5.56, N 9.88, O 25.76, S 6.92.

#### 4.3.8. 1,2:4,5-Di-*O*-isopropylidene-3-(3-nitrophenylthioureido)-3-deoxy-α-d-fructopyranose (2h)

Yield: 86% as yellow solid; mp: 102–104 °C. [*α*]^25^_D_ −116.50° (*c* 0.002, CHCl_3_). ^1^H NMR (400 MHz, CDCl_3_): *δ* 8.27 (s, 1H), 8.05 (d, 1H, *J* = 2 Hz), 7.85 (s, 1H), 7.55 (dd, 1H, ^2^*J* = 8.4, 8 Hz), 6.69 (d, 2H,^3^*J* = 8.8 Hz), 4.65–3.92 (m, 7H), 1.50 (s, 3H), 1.43 (s, 3H), 1.32 (s, 3H), 1.26 (s, 3H) ppm. ^13^C{^1^H} NMR (100 MHz, CDCl_3_): *δ* 180.0, 148.9, 139.0, 130.2, 129.6, 120.7, 118.5, 111.4, 110.1, 104.9, 72.9, 72.4, 62.2, 60.6, 55.7, 26.7, 26.1, 25.9, 25.0 ppm. IR (KBr): 3306.03, 2987.98, 2933.68, 1530.63, 1347.44, 1212.06, 1105.57, 1064.44, 1024.77, 867.8, 751.03 cm^−1^. ESI-MS (*m*/*z*): 440.5 (M^+^). Elemental analysis for C_19_H_25_N_3_O_7_S: calculated C 51.73, H 5.46, N 9.86, O 25.24, S 7.70; found C 51.46, H 5.38, N 9.99, O 25.68, S 7.49.

#### 4.3.9. 1,2-*O*-Isopropylidene-3-[3,5-bis(trifluoromethyl)phenylthioureido]-3-deoxy-β-d-fructopyranose (3a)

Yield: 77% as white solid; mp: 162–164 °C. [*α*]^25^_D_ −38.33° (*c* 0.001, CHCl_3_). ^1^H NMR (400 MHz, DMSO-d_6_): *δ* 10.33 (s, 1H), 8.37 (s, 2H), 7.75 (s, 1H), 7.68 (d, 1H), 4.92–3.31 (m, 9H), 1.39 (s, 3H), 1.38 (s, 3H) ppm. ^13^C{^1^H} NMR (100 MHz, DMSO-d_6_): *δ* 181.6, 141.7, 130.6, 130.3, 130.1, 129.9, 129.6, 127.3, 124.5, 121.8, 121.6, 119.0, 116.2, 110.3, 106.0, 71.4, 69.2, 67.8, 64.7, 54.3, 26.3, 26.2 ppm. IR (KBr): 3310, 3092, 2349, 1548, 1465, 1374, 1267, 1130, 970, 878, 795, 688 cm^−1^. ESI-MS (*m*/*z*): 491.4 (M^+^). Elemental analysis for C_18_H_20_F_6_N_2_O_5_S: calculated C 44.18, H 4.07, F 22.84, N 5.39, O 16.51, S 6.99; found C 44.36, H 4.32, F 22.66, N 5.66, O 16.26, S 6.73.

#### 4.3.10. 1,2-*O*-Isopropylidene-3-[3,5-bis(trifluoromethyl)phenylthioureido]-3-deoxy-α-d-fructopyranose (3b)

Yield: 78% as white solid; mp: 167–169 °C. [*α*]^25^_D_ −89.67° (*c* 0.001, CHCl_3_). ^1^H NMR (400 MHz, CDCl_3_): *δ* 8.26 (s, 1H), 7.85 (s, 2H), 7.70 (s, 1H), 7.48 (d, 1H, ^3^*J* = 8.4 Hz), 5.12 (s, 1H), 4.30–3.81 (m, 7H), 2.04 (s, 1H), 1.48 (s, 3H), 1.44 (s, 3H) ppm. ^13^C{^1^H} NMR (100 MHz, CDCl_3_): *δ* 182.0, 138.8, 136.2, 132.9, 131.5, 125.0, 124.4, 124.1, 119.7, 113.4, 105.1, 73.8, 68.2, 67.0, 64.1, 59.5, 27.0, 26.4 ppm. IR (KBr): 3316, 3097, 3000, 2907, 2374, 1543, 1461, 1374, 1261, 1120, 1057, 951, 868, 791 cm^−1^. ESI-MS (*m*/*z*): 491.1 (M^+^). Elemental analysis for C_18_H_20_F_6_N_2_O_5_S: calculated C 44.08, H 4.11, F 23.24, N 5.71, O 16.31, S 6.54; found C 44.13, H 4.04, F 23.38, N 5.89, O 16.28, S 6.27.

#### 4.3.11. 1,2-*O*-Isopropylidene-3-(phenylthioureido)-3-deoxy-β-d-fructopyranose (3c)

Yield: 84% as white solid; mp: 91–93 °C. [*α*]^25^_D_ −21.66° (*c* 0.001, CHCl_3_). ^1^H NMR (400 MHz, CDCl_3_): *δ* 8.53 (s, 1H), 7.45–7.28 (m, 5H), 6.24 (d, 1H, ^3^*J* = 9.6 Hz), 5.10 (d, 1H, *J* = 8 Hz), 4.00–3.77 (m, 8H), 1.37 (s, 3H), 0.94 (s, 3H) ppm. ^13^C{^1^H} NMR (100 MHz, CDCl_3_): *δ* 181.4, 138.8, 135.3, 130.3, 129.0, 127.8, 125.9, 111.2, 105.1, 76.7, 72. 6, 72.3, 68.6, 64.1, 60.4, 56.0, 25.9, 25.4 ppm. IR (KBr): 3264.31, 2923.94, 1642.98, 1536.88, 1380.98, 1245.29, 1058, 1112.34, 1011.77, 832.28, 678.07 cm^−1^. ESI-MS (*m*/*z*): 355.4 (M^+^). Elemental analysis for C_16_H_22_N_2_O_5_S: calculated C 54.22, H 6.26, N 7.90, O 22.57, S 9.05; found C 54.48, H 6.06, N 7.45, O 22.97, S 9.03.

#### 4.3.12. 1,2-*O*-Isopropylidene-3-(phenylthioureido)-3-deoxy-α-d-fructopyranose (3d)

Yield: 74% as white solid; mp: 112–115 °C. [*α*]^25^_D_ −87.50° (*c* 0.001, CHCl_3_). ^1^H NMR (400 MHz, CDCl_3_): *δ* 8.17 (s, 1H), 7.41–7.22 (m, 5H), 5.07 (s 1H), 4.25–3.64 (m, 7H), 2.93 (s, 1H), 2.03 (s, 1H), 1.46 (s, 3H), 1.43 (s, 3H) ppm. ^13^C{^1^H} NMR (100 MHz, CDCl_3_): *δ* 181.7, 135.6, 130.1, 128.5, 127.9, 125.2, 112.8, 106.2, 73.6, 67.4, 67.0, 63.6, 59.2, 26.9, 26.2 ppm. IR (KBr): 3264.31, 2923.94, 1672, 1599.89, 1382.76, 1239.09, 1088, 883.72, 758.07 cm^−1^. ESI-MS (*m*/*z*): 355.4 (M^+^). Elemental analysis for C_16_H_22_N_2_O_5_S: calculated C 54.44, H 5.96, N 7.43, O 22.87, S 9.29; found C 54.62, H 6.25, N 7.25, O 22.54, S 9.34.

#### 4.3.13. 1,2-*O*-Isopropylidene-3-(4-chlorophenylthioureido)-3-deoxy-β-d-fructopyranose (3e)

Yield: 89% as white solid; mp: 101–103 °C. [*α*]^25^_D_ −45.66° (*c* 0.002, CHCl_3_). ^1^H NMR (400 MHz, CDCl_3_): *δ* 7.97 (s, 1H), 7.44–7.22 (m, 4H), 6.09 (d, 1H, ^3^*J* = 9.2 Hz), 5.05 (t, 1H, ^3^*J* = 9.6 Hz), 4.01–3.78 (m, 7H), 2.39 (s, 1H), 1.40 (s, 3H), 0.99 (s, 3H) ppm. ^13^C{^1^H} NMR (100 MHz, CDCl_3_): *δ* 182.4, 134.2, 133.9, 130.8, 127.7, 111.6, 105.1, 73.1, 73.0, 68.7, 64.2, 56.6, 26.2, 25.3 ppm. IR (KBr): 3357.07, 2927.87, 1535.61, 1428.64, 1380.61, 1114.76, 1086.63, 885.91, 801.32, 764.11 cm^−1^. ESI-MS (*m*/*z*): 389.4 (M^+^). Elemental analysis for C_16_H_21_ClN_2_O_5_S: calculated C 49.42, H 5.44, Cl 9.12, N 7.20, O 20.57, S 8.24; found C 49.48, H 5.63, Cl 9.43, N 7.31, O 20.86, S 7.28.

#### 4.3.14. 1,2-*O*-Isopropylidene-3-(4-chlorophenylthioureido)-3-deoxy-α-d-fructopyranose (3f)

Yield: 83% as white solid; mp: 163–165 °C. [*α*]^25^_D_ −73.50° (*c* 0.002, CHCl_3_). ^1^H NMR (400 MHz, CDCl_3_): *δ* 8.50 (s, 1H), 7.42–7.19 (m, 5H), 5.06 (dd, 1H, ^3^*J* = 3.6, 3.6 Hz), 4.25–3.73 (m, 7H), 1.46 (s, 3H), 1.42 (s, 3H) ppm. ^13^C{^1^H} NMR (100 MHz, CDCl_3_): *δ* 181.7, 134.5, 133.2, 130.3, 126.5, 125.7, 115.2, 113.2, 105.0, 73.8, 67.8, 67.1, 64.0, 59.5, 27.0, 26.4 ppm. IR (KBr): 3310.76, 2933.88, 1532.48, 1378.68, 1238.62, 1133.17, 1056.20, 940.01, 862.53, 760.17, 666.90 cm^−1^. ESI-MS (*m*/*z*): 389.4 (M^+^). Elemental analysis for C_16_H_21_ClN_2_O_5_S: calculated C 49.38, H 5.51, Cl 9.09, N 7.14, O 20.87, S 8.00; found C 49.15, H 5.82, Cl 9.18, N 7.21, O 20.96, S 7.67.

#### 4.3.15. 1,2-*O*-Isopropylidene-3-(3-nitrophenylthioureido)-3-deoxy-β-d-fructopyranose (3g)

Yield: 87% as yellow solid; mp: 100–102 °C. [*α*]^25^_D_ −188.40° (*c* 0.001, CHCl_3_). ^1^H NMR (400 MHz, CDCl_3_): *δ* 8.62 (s, 1H), 8.21 (t, 1H, ^3^*J* = 2 Hz), 8.05 (d, 1H, ^3^*J* = 7.6 Hz), 7.74 (s, 1H), 7.54 (dd, 1H, ^3^*J* = 8.4, 8 Hz), 6.47 (d, 1H, ^3^*J* = 9.6 Hz), 5.09 (s, 1H), 4.07–3.82 (m, 7H), 2.39 (s, 1H), 1.42 (s, 3H), 1.25 (s, 3H) ppm. ^13^C{^1^H} NMR (100 MHz, CDCl_3_): *δ* 182.6, 149.0, 131.1, 130.7, 121.6, 120.1, 119.5, 111.7, 105.4, 72.8, 72.2, 68.9, 64.5, 56.3, 26.4, 25.5 ppm. IR (KBr): 3349.80, 2927.97, 1529.70, 1348.26, 1082.21, 882.54 cm^−1^. ESI-MS (*m*/*z*): 398.4 (M^+^). Elemental analysis for C_16_H_21_N_3_O_7_S: calculated C 48.11, H 5.30, N 10.52, O 28.04, S 8.03; found C 48.51, H 5.09, N 10.23, O 28.00, S 8.17.

#### 4.3.16. 1,2-*O*-Isopropylidene-3-(3-nitrophenylthioureido)-3-deoxy-α-d-fructopyranose (3h)

Yield: 79% as yellow solid; mp: 142–144 °C. [*α*]^25^_D_ −151.33° (*c* 0.002, CHCl_3_). ^1^H NMR (400 MHz, CDCl_3_): *δ* 8.27–7. 43 (m, 5H), 5.13 (s, 1H), 4.40–3.77 (m, 8H), 2.04 (s, 1H), 1.48 (s, 3H), 1.44 (s, 3H) ppm. ^13^C{^1^H} NMR (100 MHz, CDCl_3_): *δ* 181.9, 148.0, 138.2, 130.7, 129.9, 126.9, 121.1, 118.9, 113.2, 105.2, 73.8, 68.1, 66.9, 64.9, 59.8, 26.5, 26.2 ppm. IR (KBr): 3334.98, 2928.06, 1528.68, 1349.50, 1242.72, 1058.03, 863.24, 758.81 cm^−1^. ESI-MS (*m*/*z*): 398.4 (M^+^). Elemental analysis for C_16_H_21_N_3_O_7_S: calculated C 48.23, H 5.41, N 10.22, O 28.12, S 8.02; found C 48.32, H 5.29, N 10.43, O 27.92, S 8.03.

### 4.4. Typical procedure for enantioselective Friedel–Crafts alkylation reaction

To a stirred solution of β-nitrostyrene (0.5 mmol) in dry dichloromethane (DCM) (2 ml), was added a saccharide-based thiourea organocatalyst 3a (20 mol%) and indole (0.6 mmol). The reaction mixture was then stirred at −20 °C for the appropriate reaction time and concentrated under vacuum. The residue was then subjected to purification by column chromatography using silica gel (60–120 mesh) with a hexane : EtOAc mixture as an eluent to obtain the desired product. The enantiomeric excess of the product was determined by HPLC analysis on a chiral column using a mixture of *n*-hexane and isopropanol as the mobile phase.

#### 4.4.1. 3-(2-Nitro-1-4-chlorophenyl-ethyl)-1*H*-indole (4a)

Yield: 83% as white solid, mp: 140–142 °C; 97% ee, [*α*]^25^_D_ = −11.1 (*c* = 0.05, CH_2_Cl_2_), Chiralpak AD-H, hexane/i-PrOH = 90/10, flow rate 1.0 ml min^−1^, *t*_R_ = 35.6 min (major), *t*_R_ = 43.4 min (minor). ^1^H NMR (400 MHz, CDCl_3_): *δ* 8.10 (s, 1H), 7.39–6.97 (m, 9H), 5.16–4.85 (m, 3H) ppm. ^13^C{^1^H} NMR (100 MHz, CDCl_3_): *δ* 137.7, 136.4, 133.4, 129.1, 129.1, 128.2, 125.8, 122.8, 121.5, 120.1, 118.8, 113.8, 111.5, 76.7, 40.9 ppm. Elemental analysis for C_16_H_13_ClN_2_O_2_: calculated C 63.80, H 4.68, Cl 11.76, N 9.33, O 10.41; found C 63.78, H 4.64, Cl 12.03, N 9.22, O 10.31.

#### 4.4.2. 3-(2-Nitro-1-4-bromophenyl-ethyl)-1*H*-indole (4b)

Yield: 75% as white solid, mp: 152–154 °C; 91% ee, [*α*]^25^_D_ = −1.1 (*c* = 0.05, CH_2_Cl_2_), Chiralpak AD-H, hexane/i-PrOH = 90/10, flow rate 1.0 ml min^−1^, *t*_R_ = 34.9 min (major), *t*_R_ = 42.6 min (minor). ^1^H NMR (400 MHz, CDCl_3_): *δ* 8.11 (s, 1H), 7.44–6.99 (m, 9H), 5.14 (t, 1H, ^3^*J* = 8 Hz), 5.04 (dd, 1H, ^3^*J* = 8, 4 Hz), 4.89 (dd, 1H, ^3^*J* = 8, 4 Hz) ppm. ^13^C{^1^H} NMR (100 MHz, CDCl_3_): *δ* 138.2, 136.5, 132.0, 129.5, 125.9, 122.9, 121.5, 120.1, 118.8, 113.9, 111.4, 79.2, 41.0 ppm. Elemental analysis for C_16_H_13_BrN_2_O_2_: calculated C 55.67, H 4.08, Br 22.16, N 8.20, O 9.82; found C 55.61, H 4.11, Br 22.23, N 8.09, O 9.93.

#### 4.4.3. 3-(2-Nitro-1-4-methylphenyl-ethyl)-1*H*-indole (4c)

Yield: 64% as white solid, mp: 120–122 °C; 95% ee, [*α*]^25^_D_ = −14.5 (*c* = 0.05, CH_2_Cl_2_), Chiralpak OD-H, hexane/i-PrOH = 75/15, flow rate 0.9 ml min^−1^, *t*_R_ = 48.2 min (major), *t*_R_ = 53.1 min (minor). ^1^H NMR (400 MHz, CDCl_3_): *δ* 8.02 (s, 1H), 7.42–6.96 (m, 9H), 5.13 (t, 1H, ^3^*J* = 8 Hz) 5.01 (dd, 1H, ^3^*J* = 7.6, 7.2 Hz), 4.88 (dd, 1H, ^3^*J* = 8, 4 Hz), 2.29 (s, 3H) ppm. ^13^C{^1^H} NMR (100 MHz, CDCl_3_): *δ* 137.4, 136.7, 136.3, 129.7, 127.8, 126.3, 122.8, 121.7, 120.1, 119.1, 114.8, 111.5, 79.8, 41.4, 21.2 ppm. Elemental analysis for C_17_H_16_N_2_O_2_: calculated C 72.80, H 5.79, N 9.94, O 11.46; found C 72.82, H 5.81, N 9.96, O 11.40.

#### 4.4.4. 3-(2-Nitro-1-2-chlorophenyl-ethyl)-1*H*-indole (4d)

Yield: 74% as solid, mp: 90–92 °C; 82% ee, [*α*]^25^_D_ = −72.5 (*c* = 0.05, CH_2_Cl_2_), Chiralpak AD-H, hexane/i-PrOH = 90/10, flow rate 1.0 ml min^−1^, *t*_R_ = 34.4 min (major), *t*_R_ = 41.3 min (minor). ^1^H NMR (400 MHz, CDCl_3_): *δ* 8.11 (s, 1H), 7.44–7.07 (m, 9H), 5.74 (dd, 1H, ^3^*J* = 8, 7.6 Hz), 5.03–4.93 (m, 2H) ppm. ^13^C{^1^H} NMR (100 MHz, CDCl_3_): *δ* 136.6, 134.0, 129.1, 129.0, 127.4, 126.3, 122.9, 122.1, 120.2, 119.1, 113.5, 111.5, 77.9, 38.1 ppm. Elemental analysis for C_16_H_13_ClN_2_O_2_: calculated C 63.91, H 4.34, Cl 11.83, N 9.20, O 10.71; found C 63.88, H 4.31, Cl 11.20, N 9.15, O 11.42.

#### 4.4.5. 3-(2-Nitro-1-2-hydroxyophenyl-ethyl)-1*H*-indole (4e)

Yield: 94% as yellowish solid, mp: 109–111 °C; 96% ee, [*α*]^25^_D_ = −33.7 (*c* = 0.05, CH_2_Cl_2_), Chiralpak AD-H, hexane/i-PrOH = 90/10, flow rate 1.0 ml min^−1^, *t*_R_ = 35.2 min (major), *t*_R_ = 46.7 min (minor). ^1^H NMR (400 MHz, CDCl_3_): *δ* 8.12 (s, 1H), 7.48–6.76 (m, 9H), 5.49 (dd, 1H, ^3^*J* = 8, 7.6 Hz), 5.13–5.01 (m, 2H), 1.81 (s, 1H) ppm. ^13^C{^1^H} NMR (100 MHz, CDCl_3_): *δ* 153.6, 136.7, 129.3, 128.9, 126.5, 125.7, 122.9, 122.1, 121.5, 120.2, 119.2, 116.4, 113.7, 111.6, 78.1, 36.4 ppm. Elemental analysis for C_16_H_14_N_2_O_3_: calculated C 68.02, H 5.08, N 9.93, O 16.96; found C 68.09, H 4.99, N 9.88, O 17.02.

#### 4.4.6. 3-(2-Nitro-1-2-methoxyphenyl-ethyl)-1*H*-indole (4f)

Yield: 95% as white solid; mp: 90–92 °C, 91% ee, [*α*]^25^_D_ = −37.5 (*c* = 0.06, CH_2_Cl_2_), Chiralpak AD-H, hexane/i-PrOH = 90/10, flow rate 1.0 ml min^−1^, *t*_R_ = 34.4 min (major), *t*_R_ = 38.6 min (minor). ^1^H NMR (400 MHz, CDCl_3_): *δ* 8.06 (s, 1H), 7.47–6.83 (m, 9H), 5.59 (t, 1H, ^3^*J* = 8 Hz) 5.05–4.94 (m, 2H), 3.90 (s, 3H) ppm. ^13^C{^1^H} NMR (100 MHz, CDCl_3_): *δ* 157.1, 136.6, 129.1, 128.8, 127.4, 126.8, 122.7, 122.1, 121.0, 120.0, 119.3, 114.2, 111.4, 111.0, 78.3, 55.7, 35.8 ppm. Elemental analysis for C_17_H_16_N_2_O_2_: calculated C 68.88, H 5.50, N 9.42, O 16.16; found C 68.85, H 5.53, N 9.39, O 16.21.

#### 4.4.7. 3-(2-Nitro-1-phenylethyl)-1*H*-indole (4g)

Yield: 78% as white solid, mp: 114–116 °C; 90% ee, [*α*]^25^_D_ = −18.2 (*c* = 0.055, CH_2_Cl_2_), Chiralpak OD-H, hexane/i-PrOH = 70/30, flow rate 1 ml min^−1^, *t*_R_ = 26.2 min (major), *t*_R_ = 29.7 min (minor). ^1^H NMR (500 MHz, CDCl_3_): *δ* 8.10 (s, 1H), 7.48–6.99 (m, 10H), 5.21 (dd, 1H, ^3^*J* = 8, 4 Hz), 5.07 (dd, 1H, ^3^*J* = 8, 4 Hz), 4.95 (t, 1H, ^3^*J* = 10 Hz) ppm. ^13^C{^1^H} NMR (125 MHz, CDCl_3_): *δ* 139.2, 136.5, 128.9, 127.8, 127.6, 126.1, 122.7, 121.7, 119.9, 118.9, 114.3, 111.4, 79.5, 41.5 ppm. Elemental analysis for C_16_H_14_N_2_O_2_: calculated C 72.15, H 5.31, N 10.50, O 12.02; found C 72.11, H 5.34, N 10.43, O 12.10.

#### 4.4.8. 2-Methyl-3-(2-nitro-1-phenylethyl)-1*H*-indole (4h)

Yield: 52% as white solid, mp: 109–111 °C; 84% ee, [*α*]^25^_D_ = −3.1 (*c* = 0.05, CH_2_Cl_2_), Chiralpak AD-H, hexane/i-PrOH = 90/10, flow rate 1 ml min^−1^, *t*_R_ = 24.7 min (major), *t*_R_ = 28.9 (minor). ^1^H NMR (500 MHz, CDCl_3_): *δ* 8.05 (s, 1H), 7.58–7.21 (m, 9H), 5.43–5.28 (m, 3H), 2.51 (s, 3H) ppm. ^13^C{^1^H} NMR (125 MHz, CDCl_3_): *δ* 139.5, 135.4, 133.0, 128.8, 127.3, 127.1, 126.8, 121.3, 119.7, 118.6, 110.8, 108.7, 78.6, 40.5, 11.9 ppm. Elemental analysis for C_17_H_16_N_2_O_2_: calculated C 72.80, H 5.77, N 10.02, O 11.39; found C 72.77, H 5.81, N 10.05, O 11.35.

#### 4.4.9. 3-(2-Nitro-1-4-bromophenyl-ethyl)-1*H*-methylindole (4i)

Yield: 57% as white solid, mp: 96–98 °C; >99% ee, Chiralpak OD-H, hexane/i-PrOH = 20/70, flow rate 0.9 ml min^−1^, *t*_R_ = 15.5 min (major). ^1^H NMR (400 MHz, CDCl_3_): *δ* 7.90 (s, 1H), 7.42–7.12 (m, 8H), 5.22–5.04 (m, 3H), 2.40 (s, 3H) ppm. ^13^C{^1^H} NMR (100 MHz, CDCl_3_): *δ* 138.7, 135.6, 133.0, 132.0, 129.2, 126.8, 121.7, 121.2, 120.1, 118.6, 110.9, 108.6, 78.5, 40.1, 12.2 ppm. Elemental analysis for C_17_H_15_BrN_2_O_2_: calculated C 56.80, H 4.24, Br 22.28 N 7.78, O 8.88; found C 56.78, H 4.30, Br 21.99 N 7.80, O 9.10.

#### 4.4.10. 3-(2-Nitro-1-2-chlorophenyl-ethyl)-1*H*-methylindole (4j)

Yield: 55% as solid, mp: 119–121 °C; >99% ee, Chiralpak OD-H, hexane/i-PrOH = 20/70, flow rate 0.9 ml min^−1^, *t*_R_ = 10.1 min (major), *t*_R_ = 14.1 (minor). ^1^H NMR (400 MHz, CDCl_3_): *δ* 7.92 (s, 1H), 7.59–7.07 (m, 8H), 5.57–5.53 (m, 1H) 5.21–5.17 (m, 2H), 2.48 (s, 3H) ppm. ^13^C{^1^H} NMR (100 MHz, CDCl_3_): *δ* 136.7, 135.4, 134.1, 133.6, 130.4, 128.7, 127.1, 127.1, 121.4, 119.9, 118.5, 110.8, 107.1, 76.7, 38.5, 12.3 ppm. Elemental analysis for C_17_H_15_ClN_2_O_2_: calculated C 64.85, H 4.79, Cl 11.22, N 8.92, O 10.20; found C 64.91, H 4.74, Cl 11.15, N 8.88, O 10.30.

#### 4.4.11. 1-Methyl-3-(2-nitro-1-phenylethyl)-1*H*-indole (4k)

Yield: 46% as semisolid, mp: 93–95 °C; 66% ee, [*α*]^25^_D_ = +18.8 (*c* = 0.058, CH_2_Cl_2_), Chiralpak OD-H, hexane/i-PrOH = 85/15, flow rate 1 ml min^−1^, *t*_R_ = 73.2 min (major), *t*_R_ = 80.5 min (minor). ^1^H NMR (500 MHz, CDCl_3_): *δ* 7.50–6.89 (m, 10H), 5.23–5.20 (m, 1H), 5.07 (dd, 1H, ^3^*J* = 8, 4 Hz), 4.96 (dd, 1H, ^3^*J* = 8, 4 Hz), 3.75 (s, 3H) ppm. ^13^C{^1^H} NMR (125 MHz, CDCl_3_): *δ* 139.4, 137.3, 128.9, 127.7, 127.5, 126.5, 126.4, 122.2, 119.4, 119.0, 112.8, 109.5, 79.5, 40.5, 32.8 ppm. Elemental analysis for C_17_H_16_N_2_O_2_: calculated C 72.82, H 5.73, N 9.99, O 11.43; found C 72.75, H 5.74, N 9.92, O 11.56.

#### 4.4.12. 3-(1-(4-Bromophenyl)-2-nitroethyl)-1-methyl-1*H*-indole (4l)

Yield: 61% as white solid, mp: 80–82 °C; 82% ee, [*α*]^25^_D_ = −3.8 (*c* = 0.05, CH_2_Cl_2_), Chiralpak AD-H, hexane/i-PrOH = 90/10, flow rate 1 ml min^−1^, *t*_R_ = 9.7 min (major), *t*_R_ = 12.6 min (minor). ^1^H NMR (500 MHz, CDCl_3_): *δ* 7.47–7.10 (m, 8H), 6.87 (s, 1H), 5.16 (t, 1H, ^3^*J* = 10 Hz), 5.04 (dd, 1H, ^3^*J* = 8, 4 Hz), 4.90 (t, 1H, ^3^*J* = 8 Hz), 3.75 (s, 3H) ppm. ^13^C{^1^H} NMR (125 MHz, CDCl_3_): *δ* 138.5, 137.3, 132.0, 129.5, 129.4, 126.3, 126.3, 122.4, 121.4, 119.6, 118.9, 112.2, 109.7, 79.2, 60.4, 41.0, 32.8 ppm. Elemental analysis for C_17_H_15_BrN_2_O_2_: calculated C 56.80, H 4.33, Br 22.12, N 7.85, O 8.88; found C 56.85, H 4.39, Br 22.05, N 7.78, O 8.92.

#### 4.4.13. 1-Methyl-3-(2-nitro-1-(*p*-tolyl)ethyl)-1*H*-indole (4m)

Yield: 65% as solid, mp: 96–98 °C; 95% ee, [*α*]^25^_D_ = −19.5 (*c* = 0.05, CH_2_Cl_2_), Chiralpak AD-H, hexane/i-PrOH = 95/5, flow rate 1 ml min^−1^, *t*_R_ = 15.3 min (major), *t*_R_ = 19.8 min (minor). ^1^H NMR (500 MHz, CDCl_3_): *δ* 7.49–7.08 (m, 8H), 6.87 (s, 1H), 5.16 (t, 1H, *J* = 8 Hz), 5.05 (dd, 1H, ^3^*J* = 8, 4 Hz), 4.93 (dd, 1H, ^3^*J* = 8, 4 Hz), 3.74 (s, 3H), 2.33 (s, 3H) ppm. ^13^C{^1^H} NMR (125 MHz, CDCl_3_): *δ* 137.3, 137.1, 136.3, 130.1, 129.6, 127.6, 126.5, 126.3, 122.2, 119.4, 119.0, 113.0, 109.5, 79.6, 41.2, 32.8, 21.1 ppm. Elemental analysis for C_18_H_18_N_2_O_2_: calculated C 73.45, H 6.10, N 9.48, O 10.95; found C 73.51, H 6.17, N 9.38, O 10.92.

#### 4.4.14. 1-Benzyl-3-(2-nitro-1-phenylethyl)-1*H*-indole (4n)

Yield: 52% as semisolid, mp: 109–111 °C; 75% ee, [*α*]^25^_D_ = +22.5 (*c* = 0.066, CH_2_Cl_2_), Chiralpak AD-H, hexane/i-PrOH = 90/10, flow rate 1 ml min^−1^, *t*_R_ = 11.0 min (major), *t*_R_ = 14.5 min (minor). ^1^H NMR (500 MHz, CDCl_3_): *δ* 7.62–7.00 (m, 15H), 5.30 (s, 2H), 5.22 (dd, 1H, ^3^*J* = 8, 4 Hz), 5.07 (dd, 1H, ^3^*J* = 8, 4 Hz), 4.96 (dd, 1H, ^3^*J* = 8, 4 Hz) ppm. ^13^C{^1^H} NMR (125 MHz, CDCl_3_): *δ* 139.3, 137.2, 136.9, 128.9, 128.8, 127.8, 127.7, 127.5, 126.6, 119.7, 113.5, 79.6, 50.1, 41.6 ppm. Elemental analysis for C_23_H_20_N_2_O_2_: calculated C 77.50, H 5.68, N 7.90, O 8.90; found C 77.53, H 5.62, N 7.96, O 8.88.

#### 4.4.15. 5-Methyl-3-(2-nitro-1-phenylethyl)-1*H*-indole (4o)

Yield: 46% as white solid, mp: 93–95 °C; 82% ee, [*α*]^25^_D_ = +8.5 (*c* = 0.066, CH_2_Cl_2_), Chiralpak OD-H, hexane/i-PrOH = 96/4, flow rate 1 ml min^−1^, *t*_R_ = 35.6 min (major), *t*_R_ = 63.6 min (minor). ^1^H NMR (500 MHz, CDCl_3_): *δ* 7.96 (s, 1H), 7.36–6.93 (m, 9H), 5.19 (t, 1H, ^3^*J* = 8 Hz), 5.06 (dd, 1H, ^3^*J* = 8, 4 Hz), 4.95 (dd, 1H, *J* = 8, 4 Hz), 4.96 (t, 1H, ^3^*J* = 4 Hz), 2.44 (s, 3H) ppm. ^13^C{^1^H} NMR (125 MHz, CDCl_3_): *δ* 139.3, 134.8, 129.3, 128.9, 127.8, 127.5, 126.3, 124.3, 121.8, 118.4, 113.8, 111.1, 79.5, 41.5, 21.5 ppm. Elemental analysis for C_17_H_16_N_2_O_2_: calculated C 72.80, H 5.73, N 9.95, O 11.51; found C 72.77, H 5.79, N 9.90, O 11.53.

#### 4.4.16. 2-(2-Nitro-1-phenylethyl)-1*H*-pyrrole (5a)

Yield: 41% as white crystal, mp: 75–77 °C; 87% ee, [*α*]^25^_D_ = −72.1 (*c* = 0.088, MeOH), Chiralpak OD-H, hexane/i-PrOH = 80/20, flow rate 1 ml min^−1^, *t*_R_ = 25.0 min (major), *t*_R_ = 28.2 min (minor). ^1^H NMR (500 MHz, CDCl_3_): *δ* 7.94 (s, 1H), 7.38–7.23 (m, 5H), 6.68–6.67 (m, 1H), 6.19–6.18 (m, 1H), 6.11–6.09 (m, 1H), 4.97 (dd, 1H, ^3^*J* = 8, 4 Hz), 4.90 (dd, 1H, *J* = 8, 4 Hz), 4.80 (dd, 1H, ^3^*J* = 8, 4 Hz) ppm. ^13^C{^1^H} NMR (125 MHz, CDCl_3_): *δ* 138.1, 129.2, 128.9, 128.1, 127.9, 118.3, 108.6, 105.8, 79.2, 42.9 ppm. Elemental analysis for C_12_H_12_N_2_O_2_: calculated C 66.60, H 5.63, N 12.88, O 14.86; found C 66.64, H 5.59, N 12.83, O 14.92.

#### 4.4.17. 2-(1-(2-Chlorophenyl)-2-nitroethyl)-1*H*-pyrrole (5b)

Yield: 52% as yellowish oil, mp: 99–101 °C; 80% ee, [*α*]^25^_D_ = −35.7 (*c* = 0.04, MeOH), Chiralpak AD-H, hexane/i-PrOH = 90/10, flow rate 1 ml min^−1^, *t*_R_ = 16.5 min (major), *t*_R_ = 27.9 min (minor). ^1^H NMR (500 MHz, CDCl_3_): *δ* 8.11 (s, 1H), 7.25–7.13 (m, 4H), 6.71–6.70 (m, 1H), 6.19–6.14 (m, 2H), 4.95–4.79 (m, 3H) ppm. ^13^C{^1^H} NMR (125 MHz, CDCl_3_): *δ* 137.7, 133.5, 130.2, 129.2, 129.0, 127.8, 127.6, 118.4, 108.7, 106.3, 77.3, 39.3 ppm. Elemental analysis for C_12_H_11_ClN_2_O_2_: calculated C 57.55, H 4.47, Cl 14.10, N 11.18, O 12.68; found C 57.50, H 4.41, Cl 14.19, N 11.23, O 12.65.

#### 4.4.18. 2-(1-(4-Chlorophenyl)-2-nitroethyl)-1*H*-pyrrole (5c)

Yield: 45% as white solid, mp: 97–99 °C; 82% ee, [*α*]^25^_D_ = −42.2 (*c* = 0.08, MeOH), Chiralpak OD-H, hexane/i-PrOH = 80/20, flow rate 1 ml min^−1^, *t*_R_ = 10.2 min (major), *t*_R_ = 13.6 min (minor). ^1^H NMR (500 MHz, CDCl_3_): *δ* 7.89 (s, 1H), 7.49–7.46 (m, 2H), 7.12–7.10 (m, 2H), 6.71–6.70 (m, 1H), 6.18–6.16 (m, 1H), 6.08–6.07 (m, 1H), 4.96 (dd, 1H, ^3^*J* = 8, 4 Hz), 4.86 (dd, 1H, ^3^*J* = 8, 4 Hz), 4.77 (dd, 1H, ^3^*J* = 8, 4 Hz) ppm. ^13^C{^1^H} NMR (125 MHz, CDCl_3_): *δ* 137.1, 132.3, 129.6, 128.2, 122.1, 118.5, 108.7, 106.0, 78.9, 42.4 ppm. Elemental analysis for C_12_H_11_ClN_2_O_2_: calculated C 57.53, H 4.31, Cl 15.02, N 11.01, O 12.12; found C 57.46, H 4.29, Cl 15.09, N 11.12, O 12.03.

#### 4.4.19. 2-(2-Nitro-1-(*p*-tolyl)ethyl)-1*H*-pyrrole (5d)

Yield: 44% as white solid, mp: 94–96 °C; 93% ee, [*α*]^25^_D_ = −55.6 (*c* = 0.055, MeOH), Chiralpak OD-H, hexane/i-PrOH = 80/20, flow rate 1 ml min^−1^, *t*_R_ = 12.5 min (major), *t*_R_ = 15.3 min (minor). ^1^H NMR (500 MHz, CDCl_3_): *δ* 7.85 (s, 1H), 7.26–7.12 (m, 4H), 6.68–6.67 (m, 1H), 6.18–6.08 (m, 2H), 4.96 (dd, 1H, ^3^*J* = 8, 4 Hz), 4.86 (dd, 1H, ^3^*J* = 8, 4 Hz), 4.78 (dd, 1H, ^3^*J* = 8, 4 Hz), 2.35 (s, 3H) ppm. ^13^C{^1^H} NMR (125 MHz, CDCl_3_): *δ* 137.9, 134.9, 129.9, 129.1, 127.8, 118.1, 108.6, 105.6, 79.3, 42.6, 21.0 ppm. Elemental analysis for C_13_H_14_N_2_O_2_: calculated C 67.79, H 6.12, N 12.18, O 13.89; found C 67.82, H 6.07, N 12.23, O 13.87.

## Data availability

The data supporting this article have been included as part of the ESI.†

## Conflicts of interest

There are no conflicts of interest to declare.

## Supplementary Material

RA-015-D5RA00456J-s001

## References

[cit1] Ahmad T., Khan S., Ullah N. (2022). ACS Omega.

[cit2] Mohammadi Ziarani G., Moradi R., Ahmadi T., Lashgari N. (2018). RSC Adv..

[cit3] Ibáñez I., Kaneko M., Kamei Y., Tsutsumi R., Yamanaka M., Akiyama T. (2019). ACS Catal..

[cit4] Fan Y., Kass S. R. (2017). J. Org. Chem..

[cit5] Liu H., Xu J., Du D. M. (2007). Org. Lett..

[cit6] Namboothiri I. N. N. , Bhati M., Ganesh M., Hosamani B., Baiju T. V., Manchery S. and Bera K., Catalytic Asymmetric Friedel–Crafts Reactions of Nitroalkenes, CRC Press, 1st edn, 2020

[cit7] Herrera R. P., Sgarzani V., Bernardi L., Ricci A. (2005). Angew. Chem., Int. Ed..

[cit8] Fleming E. M., McCabe T., Connon S. J. (2006). Tetrahedron Lett..

[cit9] Dündar E., Tanyeli C. (2021). Tetrahedron Lett..

[cit10] Tang H. Y., Zhang Z. B. (2011). Phosphorus, Sulfur, Silicon Relat. Elem..

[cit11] Zhuang W., Hazell R. G., Jørgensen K. A. (2005). Org. Biomol. Chem..

[cit12] Liu Y., Zhou X., Shang D., Liu X., Feng X. (2010). Tetrahedron.

[cit13] Liu H., Lu S. F., Xu J., Du D. M. (2008). Chem.–Asian J..

[cit14] O'Reilly S., Aylward M., Keogh-Hansen C., Fitzpatrick B., McManus H. A., Müller-Bunz H., Guiry P. J. (2015). J. Org. Chem..

[cit15] Venkatanna K., Yeswanth Kumar S., Karthick M., Padmanaban R., Ramaraj Ramanathan C. (2019). Org. Biomol. Chem..

[cit16] Das T., Mohapatra S., Mishra N. P., Nayak S., Raiguru B. P. (2021). ChemistrySelect.

[cit17] García Mancheño O., Waser M. (2023). Eur. J. Org. Chem..

[cit18] Becker C., Hoben C., Kunz H. (2007). Adv. Synth. Catal..

[cit19] Kunz H., Rück K. (1993). Angew. Chem., Int. Ed. Engl..

[cit20] Agarwal J., Peddinti R. K. (2011). J. Org. Chem..

[cit21] Faísca Phillips A. M. (2014). Eur. J. Org. Chem..

[cit22] Benessere V., Del Litto R., De Roma A., Ruffo F. (2010). Coord. Chem. Rev..

[cit23] Stern R., Jedrzejas M. J. (2008). Chem. Rev..

[cit24] Mlynarski J., Gut B. (2012). Chem. Soc. Rev..

[cit25] Le G. T., Abbenante G., Becker B., Grathwohl M., Halliday J., Tometzki G., Zuegg J., Meutermans W. (2003). Drug Discovery Today.

[cit26] Boysen M. M. K. (2007). Chem.–Eur. J..

[cit27] Rončák R., Tvrdoňová M., Gonda J., Elečko J. (2020). Tetrahedron.

[cit28] Jiang X., Zhang Y., Liu X., Zhang G., Lai L., Wu L., Zhang J., Wang R. (2009). J. Org. Chem..

[cit29] Lu A., Gao P., Wu Y., Wang Y., Zhou Z., Tang C. (2009). Org. Biomol. Chem..

[cit30] Gao P., Wang C., Wu Y., Zhou Z., Tang C. (2008). Eur. J. Org. Chem..

[cit31] Azad C. S., Khan A., Narula A. K. (2016). Org. Biomol. Chem..

[cit32] Ágoston K., Fügedi P. (2014). Carbohydr. Res..

[cit33] Puglisi A., Benaglia M., Raimondi L., Lay L., Poletti L. (2011). Org. Biomol. Chem..

[cit34] Hai M., Liu K., Zhang F. G., Le Zhu C., Nie J., Jun-An M. (2010). J. Org. Chem..

[cit35] Reddy B. V. S., Reddy S. M., Swain M. (2013). RSC Adv..

[cit36] Pu X., Li P., Peng F., Li X., Zhang H., Shao Z. (2009). Eur. J. Org. Chem..

[cit37] Gu Q., Guo X. T., Wu X. Y. (2009). Tetrahedron.

[cit38] Adam L., Schefzig L., Pecchioli T., Zimmer R., Reissig H. U. (2018). Synth. Commun..

[cit39] Subba Reddy B. V., Madhusudana Reddy S., Swain M., Dudem S., Kalivendi S. V., Suresh Reddy C. (2014). RSC Adv..

[cit40] Yuan H.-N., Wang S., Nie J., Meng W., Yao Q., Ma J.-A. (2013). Angew. Chem..

[cit41] Turks M., Rolava E., Stepanovs D., Mishnev A., Marković D. (2015). Tetrahedron:Asymmetry.

[cit42] Vanlaldinpuia K., Bora P., Bez G. (2017). J. Chem. Sci..

[cit43] Vanlaldinpuia K., Bora P., Basumatary G., Mohanta R., Bez G. (2017). J. Chem. Sci..

[cit44] Vanlaldinpuia K., Bez G. (2011). Tetrahedron Lett..

[cit45] Lalhmangaihzuala S., Khiangte V., Laldinpuii Z., Nunnemi L., Malsawmsanga J., Lallawmzuali G., Liana T., Lalhriatpuia C., Pachuau Z., Vanlaldinpuia K. (2023). Chemistry.

[cit46] Lalhmangaihzuala S., Laldinpuii Z., Lalthanpuii P. B., Pachuau Z., Vanlaldinpuia K., Lalchhandama K. (2024). J. Appl. Pharm. Sci..

[cit47] Wang C., Zhou Z., Tang C. (2008). Org. Lett..

[cit48] Lu S. F., Du D. M., Xu J. (2006). Org. Lett..

[cit49] Tu Y., Wang Z., Frohn M., He M., Yu H., Tang Y., Shi Y. (1998). J. Org. Chem..

