# Supplementary material for: Saccharide success: exploring the role of d-fructose-based thioureas as organocatalysts for the enantioselective Friedel–Crafts alkylation reaction
Source: RSC Adv. 2025 Mar 18;15(11):8335–45. doi: 10.1039/d5ra00456j (PMC11915211; doi:10.1039/d5ra00456j)
Supplement: RA-015-D5RA00456J-s001 [file RA-015-D5RA00456J-s001.pdf]

Supplementary Information (SI) for RSC Advances.
This journal is © The Royal Society of Chemistry 2025

Saccharide success: Exploring the role of D-fructose-based thioureas as organocatalyst for the enantioselective Friedel-Crafts Alkylation reaction

Samson Lalhmangaihzuala,[a] K. Vanlalngaihawma,[a,b] K. Vanlaldinpuia[a*]

[a] Department of Chemistry, Pachhunga University College, Mizoram University, Aizawl, 796001, Mizoram, India

[b] Department of Chemistry, Mizoram University, Tanhril, Aizawl, 796004, Mizoram, India

*Corresponding author: mapuiakhiangte@gmail.com,

Table of content

Preparation of the catalysts: Page **1-2**.

Copies of ^{1}H and ^{13}C NMR Spectra of D-fructose derived-thioureas **2a-2h** and **3a-3h**: Page **3-18**.

Copies of ^{1}H and ^{13}C NMR Spectra of Friedel-Crafts adducts **4a-4o** and **5a-5d**: Page **19-37**.

Copies of HPLC data of compounds **4a-4o** and **5a-5d**: Page **38-56**.

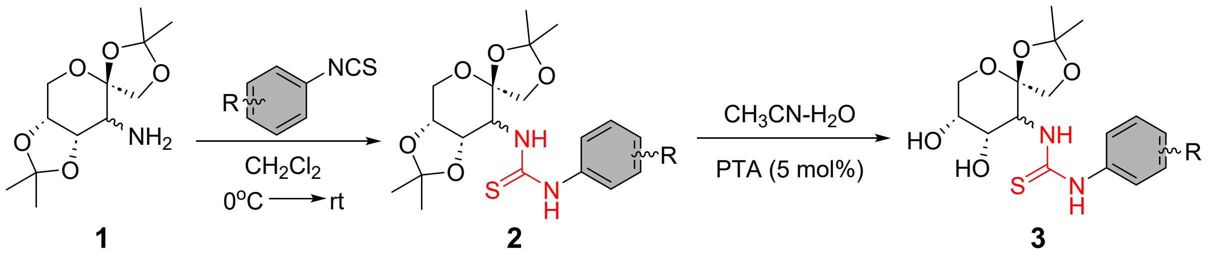

Scheme 1: Preparation of bifunctional saccharide-based thiourea organocatalyst.

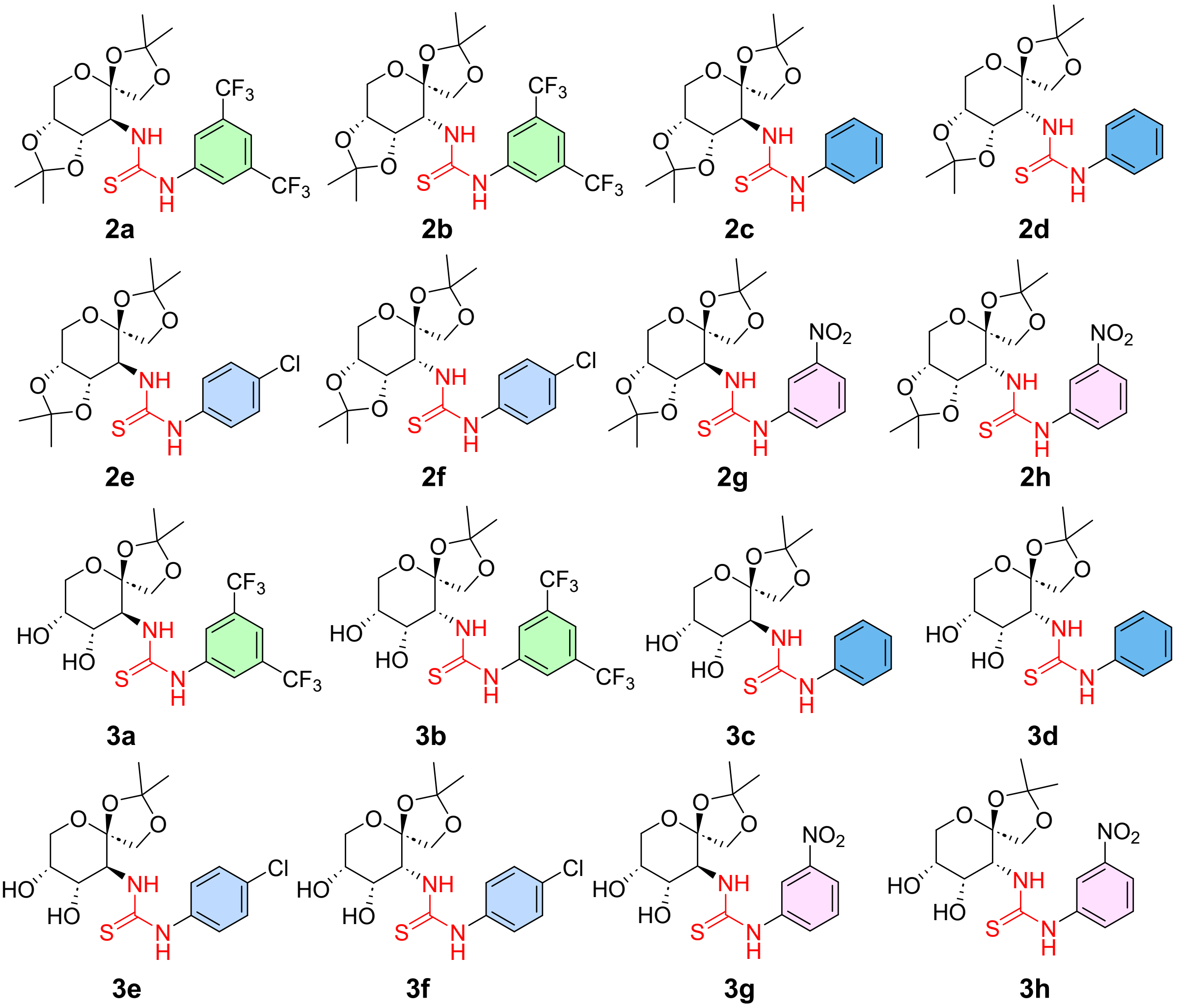

Figure 1: D-fructose-derived thioureas employed for the Friedel-Crafts Alkylation reaction.

1. Preparation of carbohydrates derived thioureas 2a-2h[1]**:** A solution of 1 mmol of the corresponding isothiocyanate in 2 ml of dry DCM was mixed with 1 mmol (0.258 mg) of sugar amines **1** (prepared according to literature [2]) at 0 ˚C. Then, the reaction mixture was permitted to reach room temperature and was stirred for another 48 hrs. The mixture was then extracted using ethyl acetate and washed with water (2x). The organic layer was dried over Na_2SO_4, filtered, evaporated and the pure product was obtained by column chromatography using 20% ethyl acetate in hexane as an eluent.

2. Synthesis of carbohydrates derived catalysts 3a-3h[1]*:* To a 100 mL round bottom flask charged with 5 mol% of phosphotungstic acid (144 mg) and 1 mmol of the corresponding thiourea compound (**2a-h**), 5 mL of CH_3CN:H_2O (9:1 ratio) mixture was added.[3] The reaction mixture was allowed to be stirred at room temperature for 6 hrs. After the reaction was completed, the solvents were evaporated under reduced pressure, and the mixture was diluted with ethyl acetate (EtOAc) and washed with water (3x). The organic layer was separated and dried using anhydrous Na_2SO_4. After filtration, the organic layer was concentrated to give the crude product which was further purified by column chromatography using 40% ethyl acetate in hexane as an eluent and silica gel (60-120 mesh) as a stationary phase to obtain the pure product. The partial deisopropylidenation of **2a-h** to afford ***3a-h*** could also be performed according to the procedure reported by Shi and co-workers.[4] The thiourea derivative (**2a-h**, 1 mmol) was dissolved in 5mL of CH_3CN:H_2O (9:1) mixture, and 10 mol% of DDQ (22 mg) was added. The reaction was completed after stirring the mixture at room temperature for 8 hrs. The solvent was evaporated, and the residue obtained was dissolved in EtOAc, washed with water (2x) and dried over anhydrous Na_2SO_4. The crude product obtained was purified by column chromatography using 40% EtOAc in hexane as an eluent.

Figure S1: ^{1}H NMR and ^{13}C of **2a.**

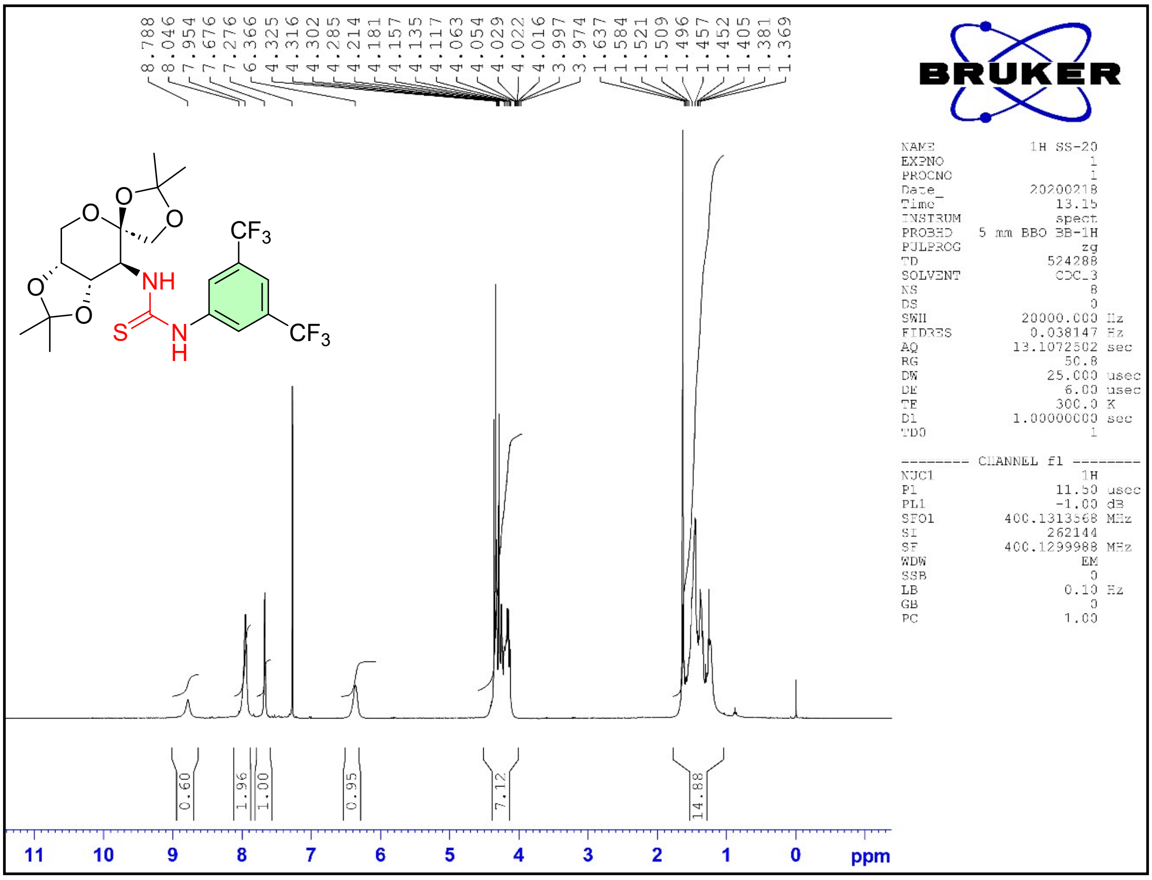

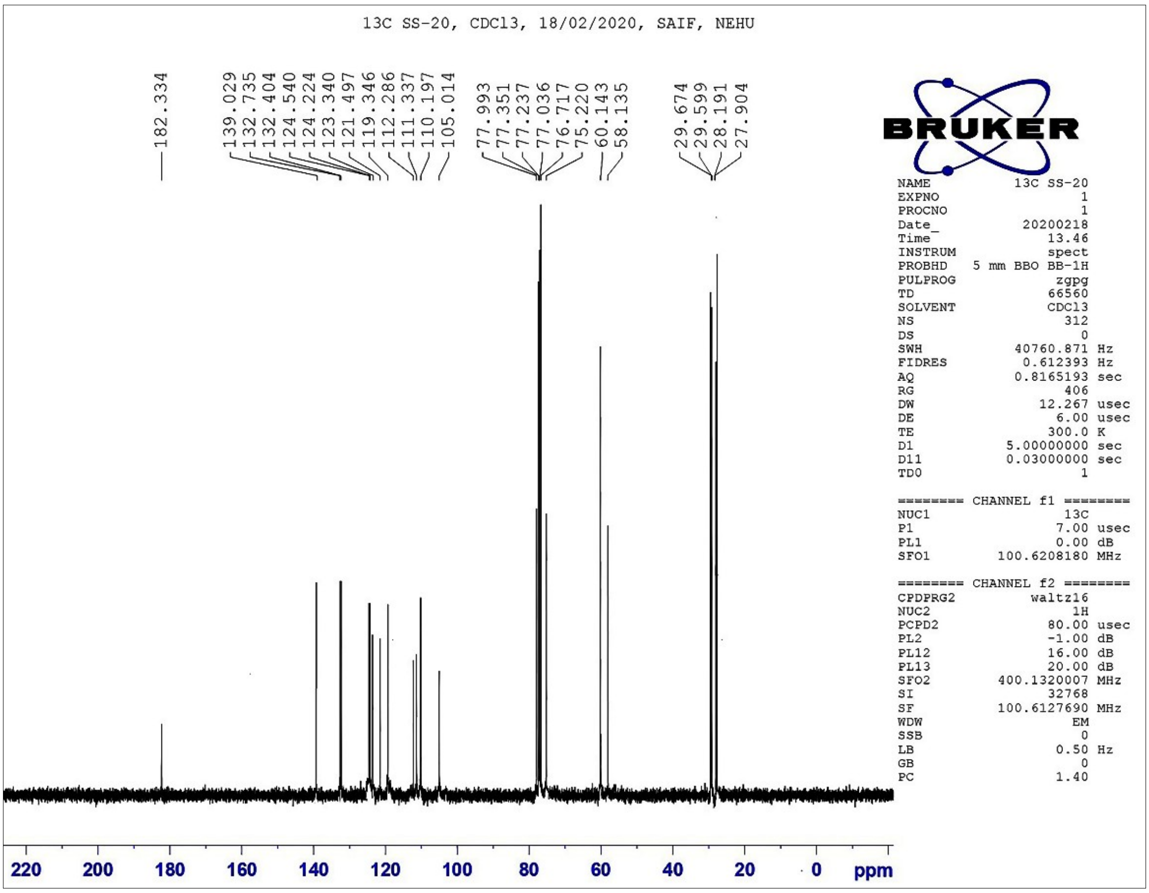

Figure S2: ^{1}H NMR and ^{13}C of **2b.**

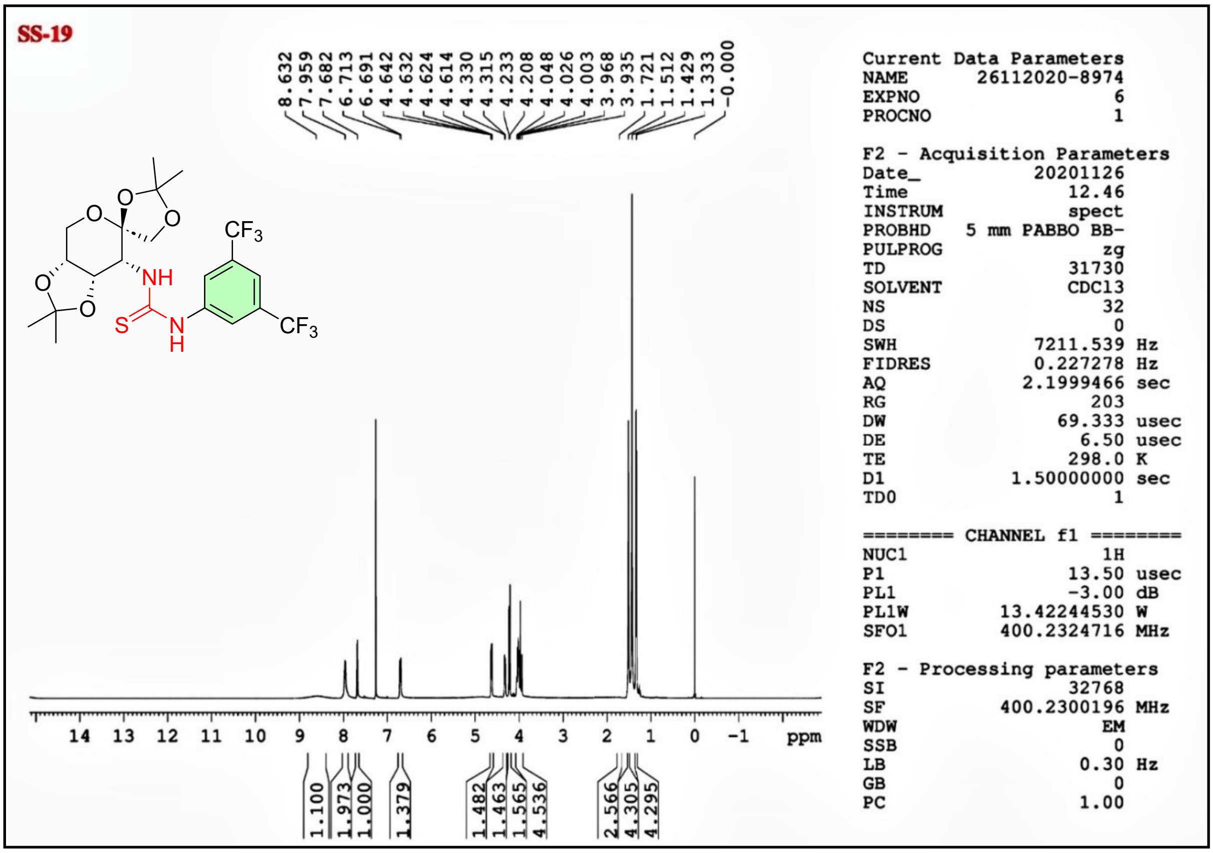

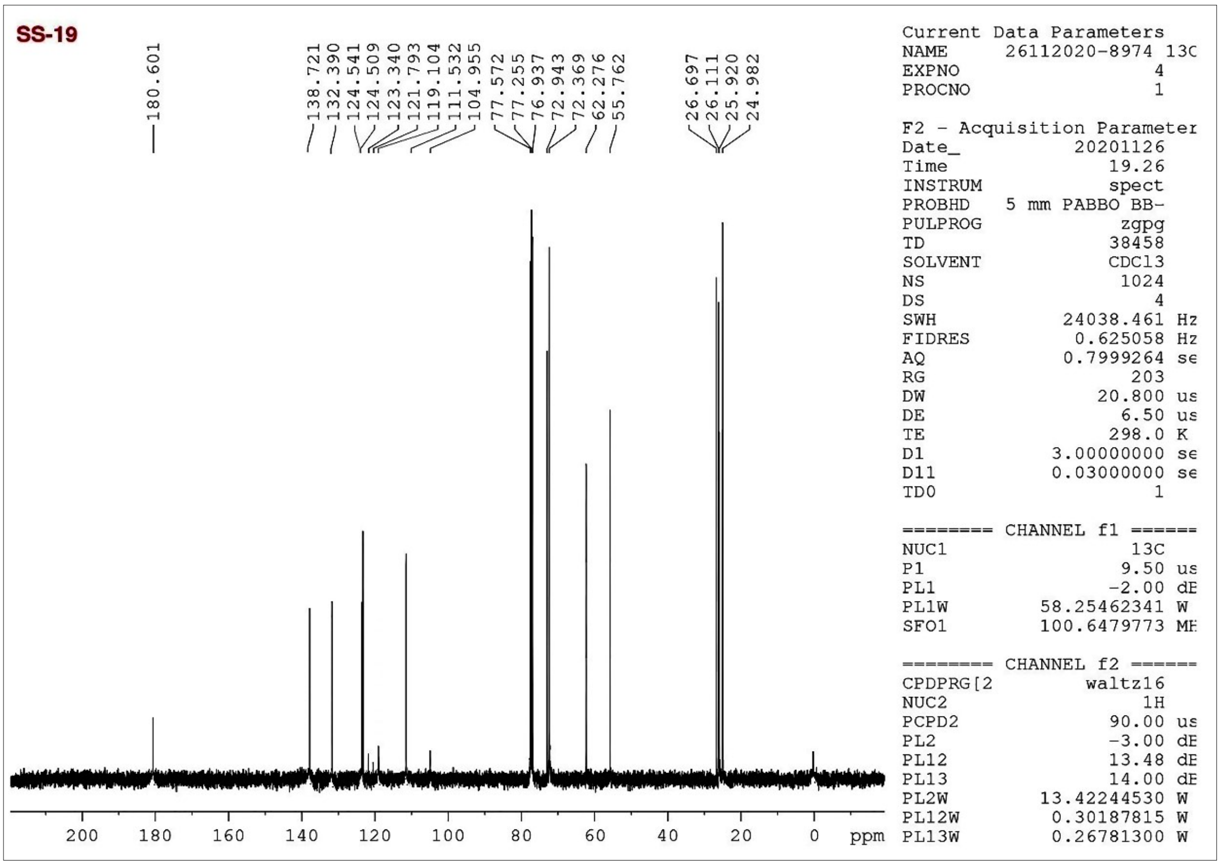

Figure S3: ^{1}H NMR and ^{13}C NMR of **2c.**

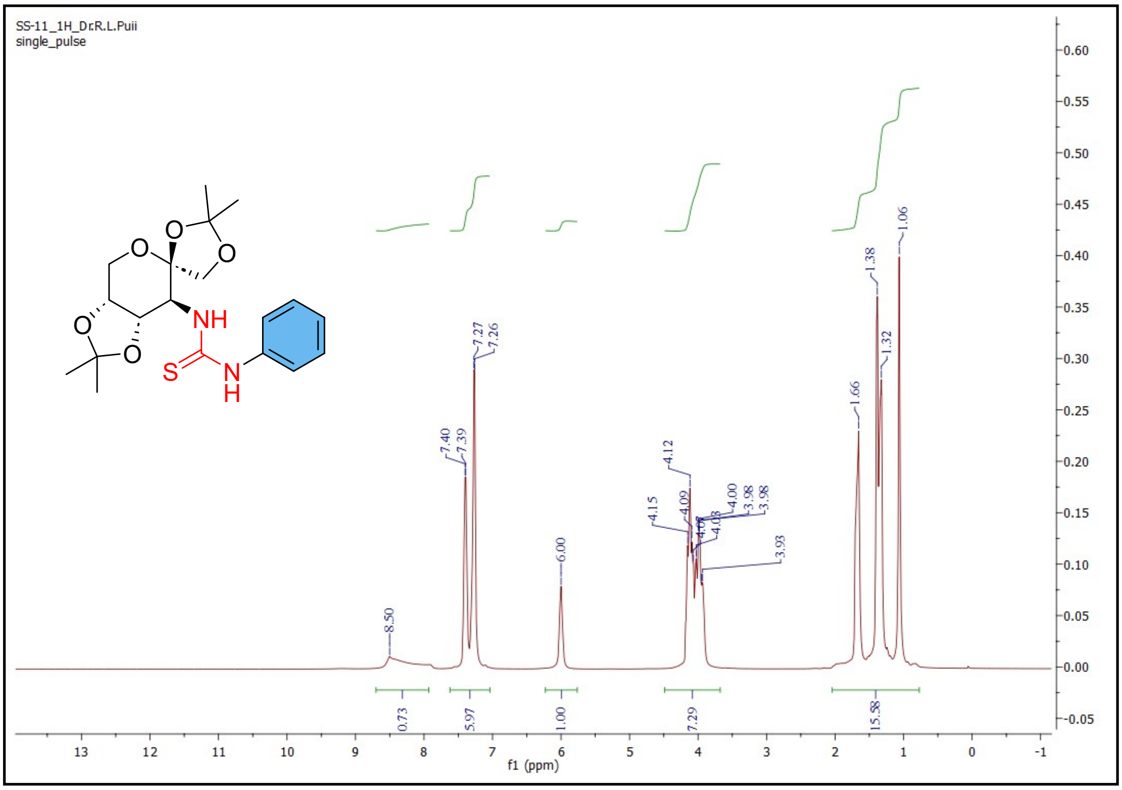

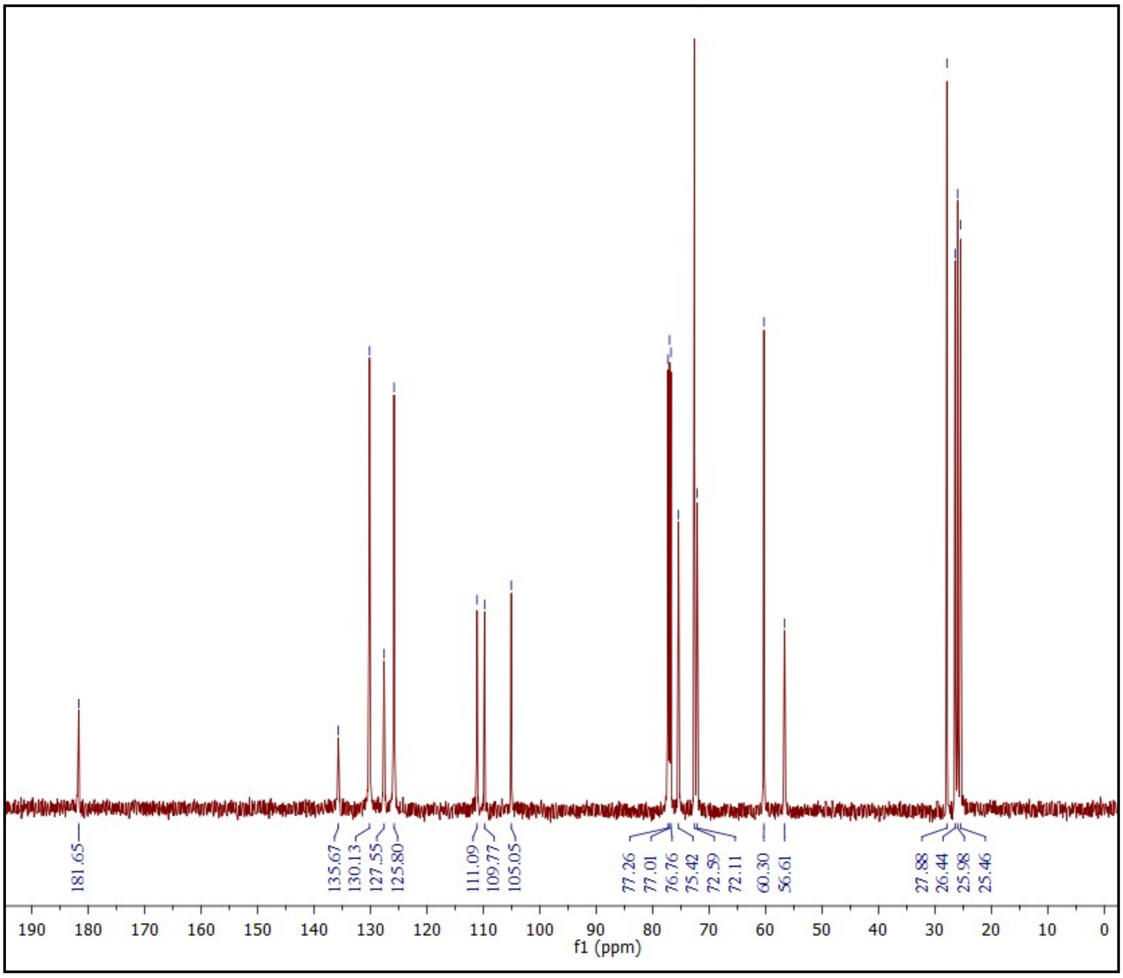

Figure S4: ^{1}H NMR and ^{13}C NMR of **2d**.

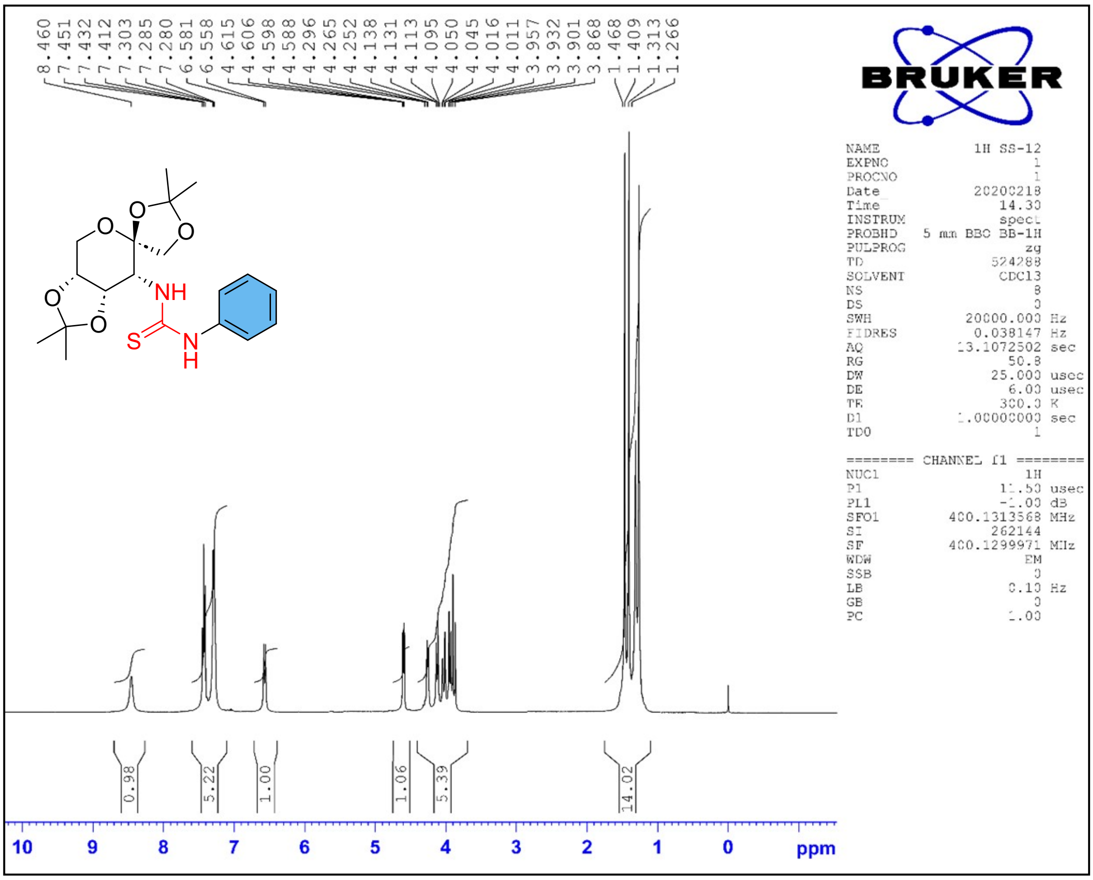

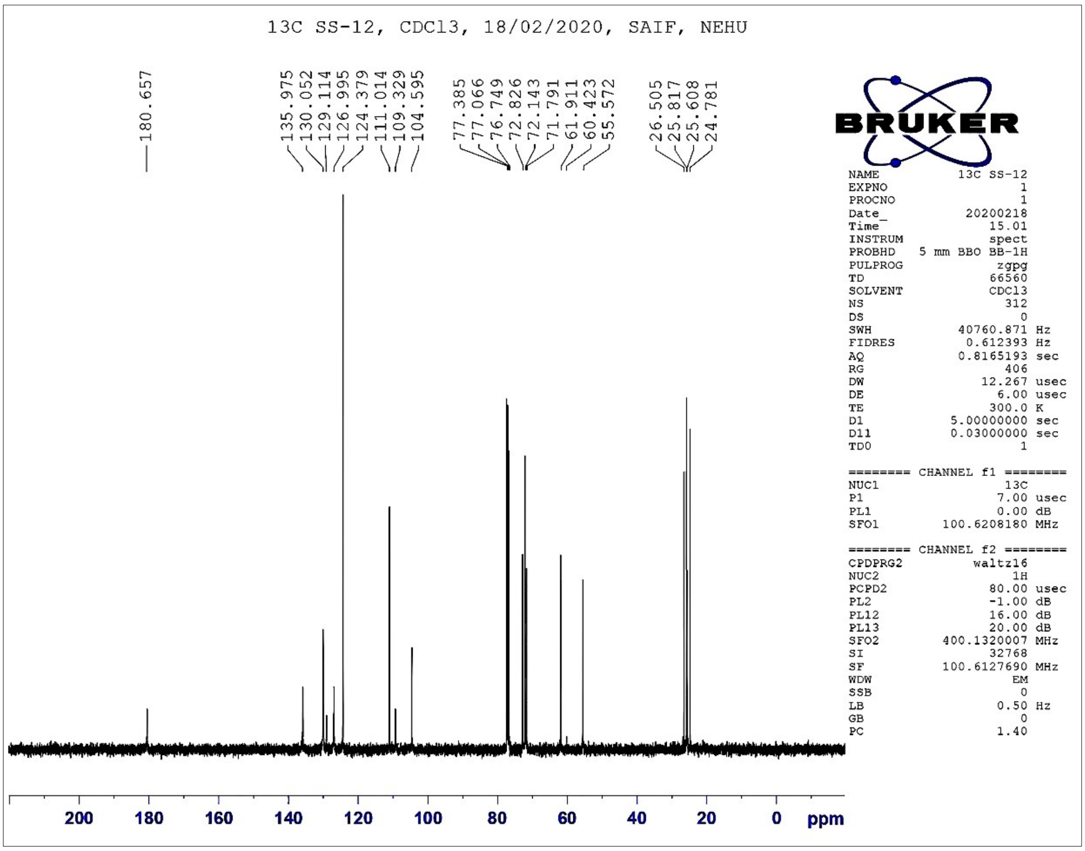

Figure S5: ^{1}H NMR and ^{13}C NMR of **2e**.

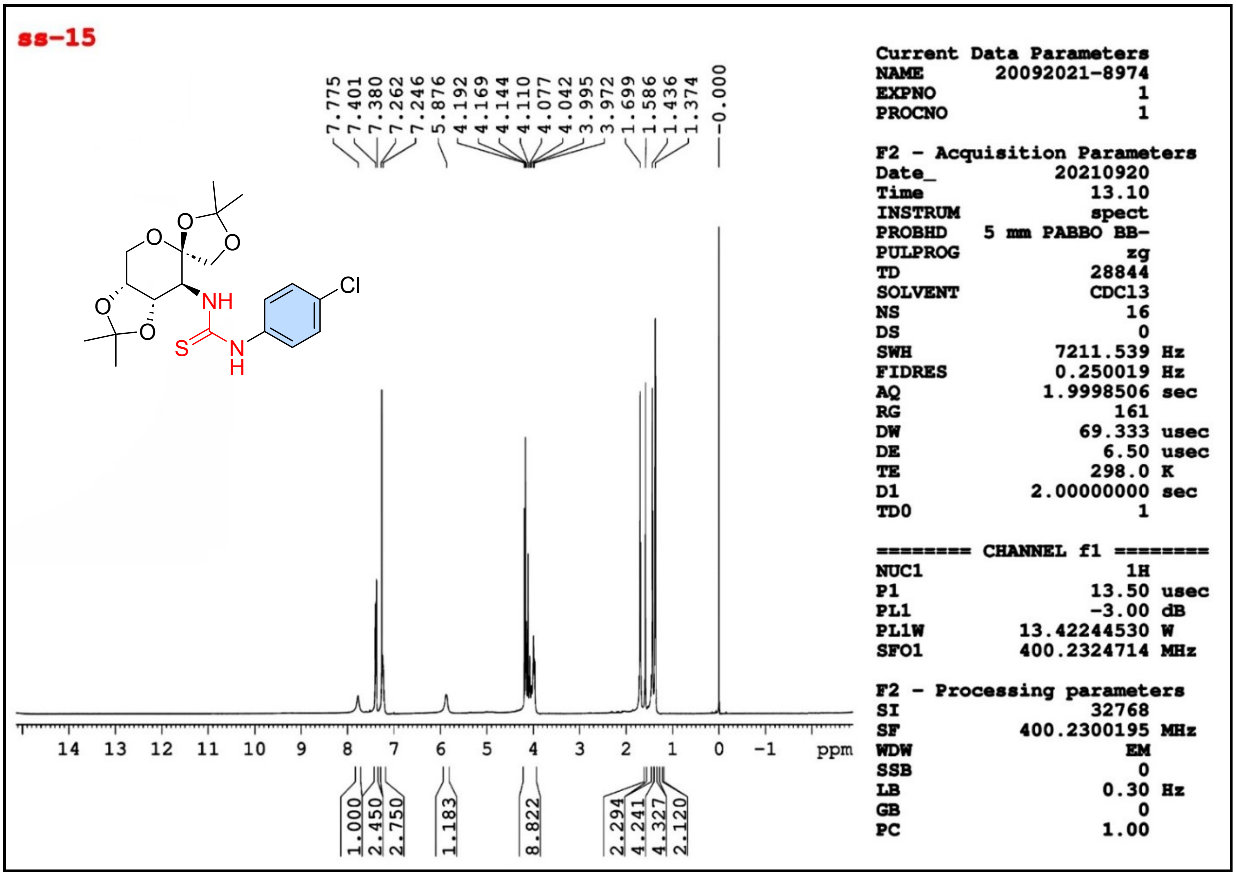

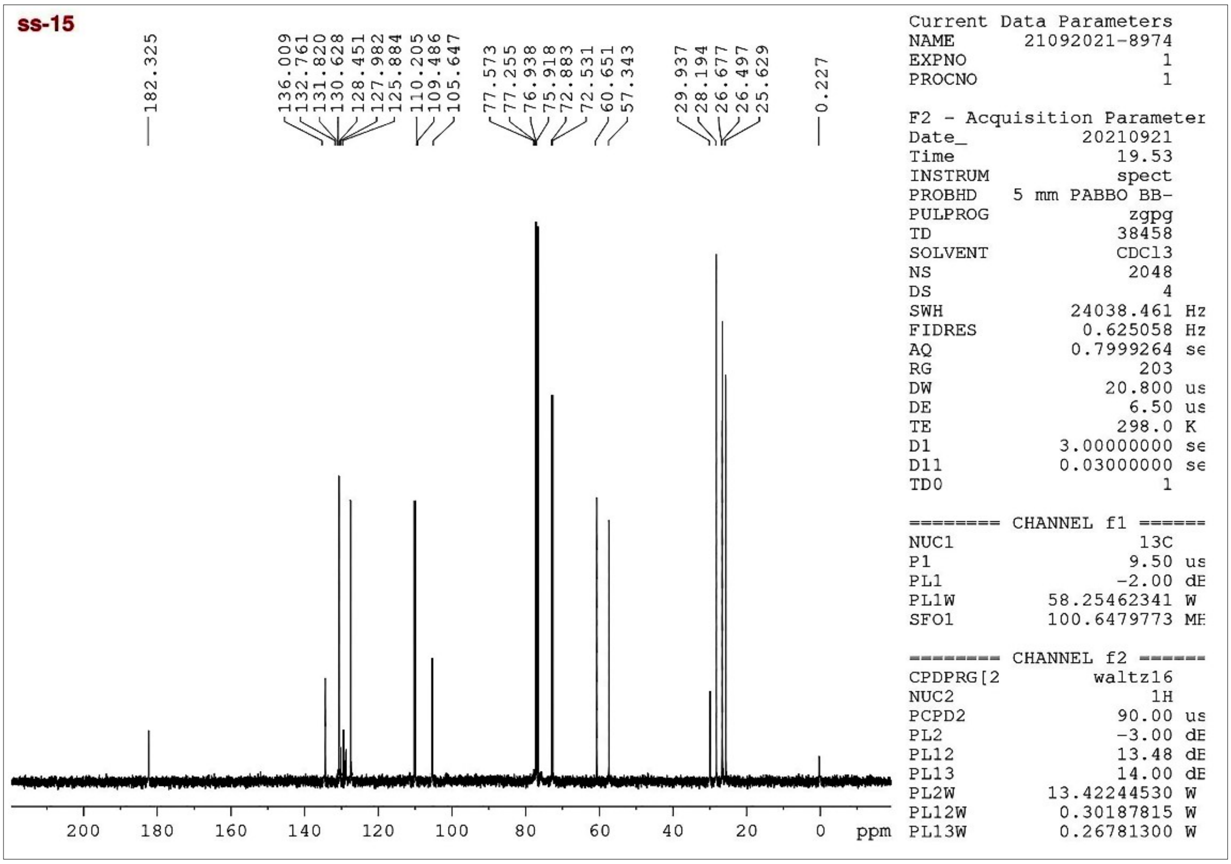

Figure S6: ^{1}H NMR and ^{13}C NMR of **2f.**

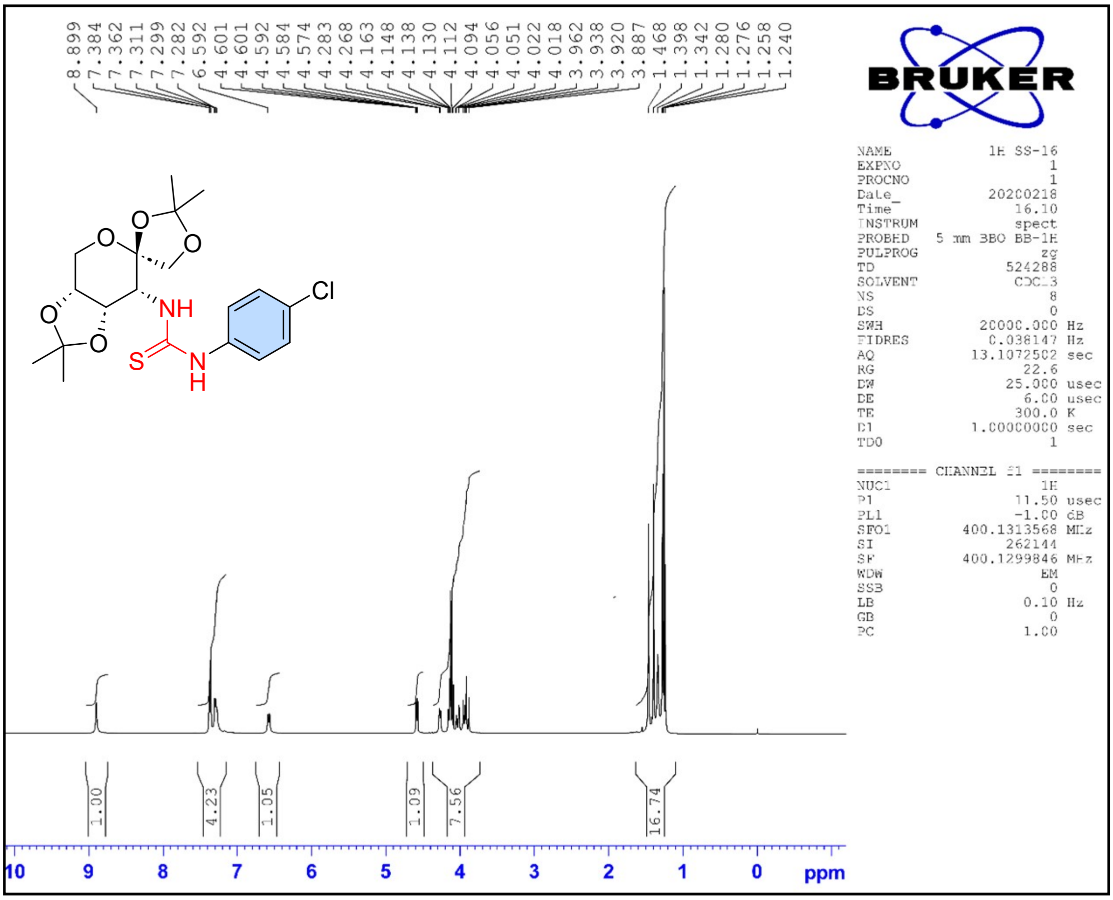

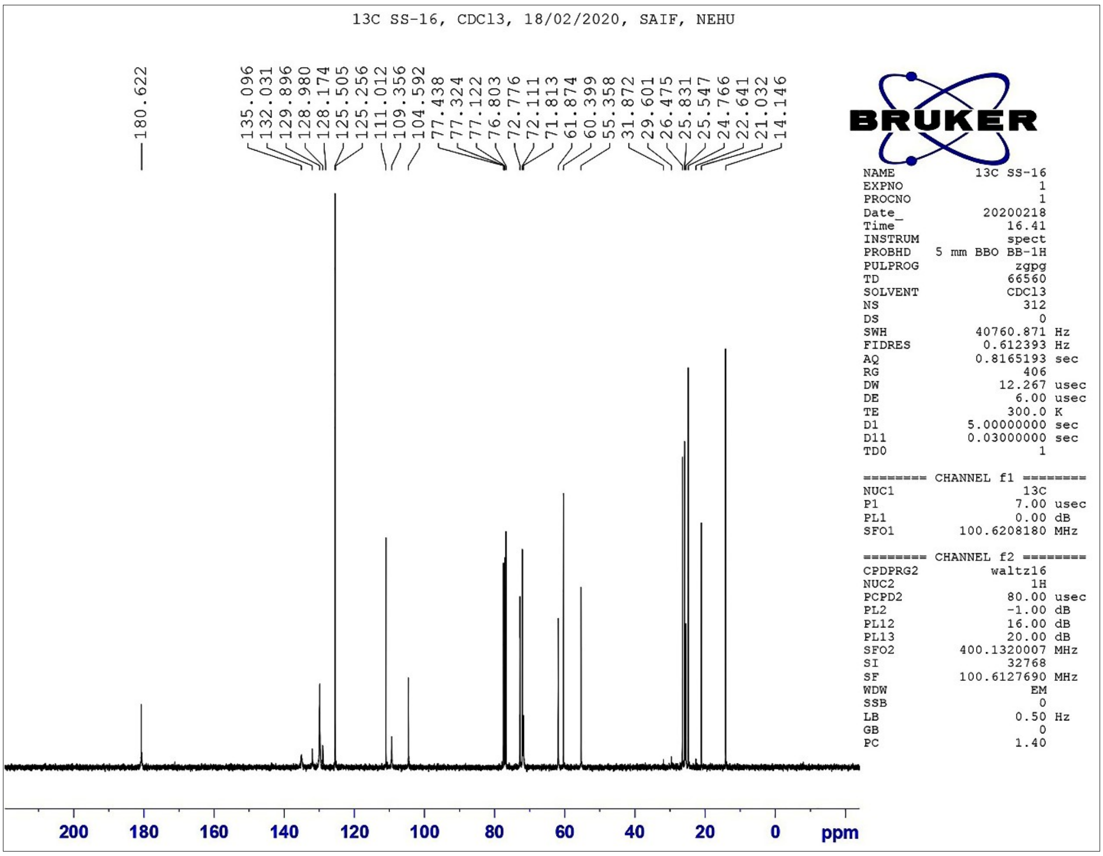

Figure S7: ^{1}H NMR and ^{13}C NMR of **2g**.

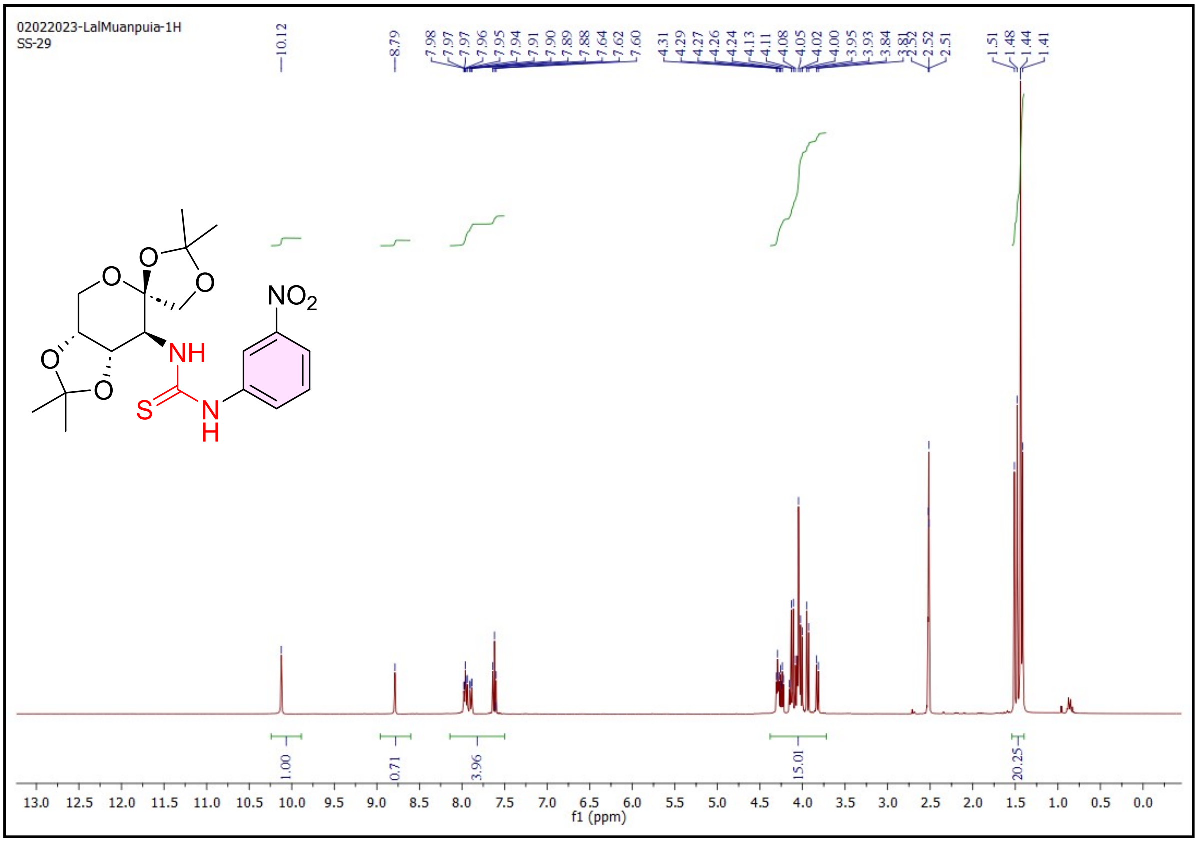

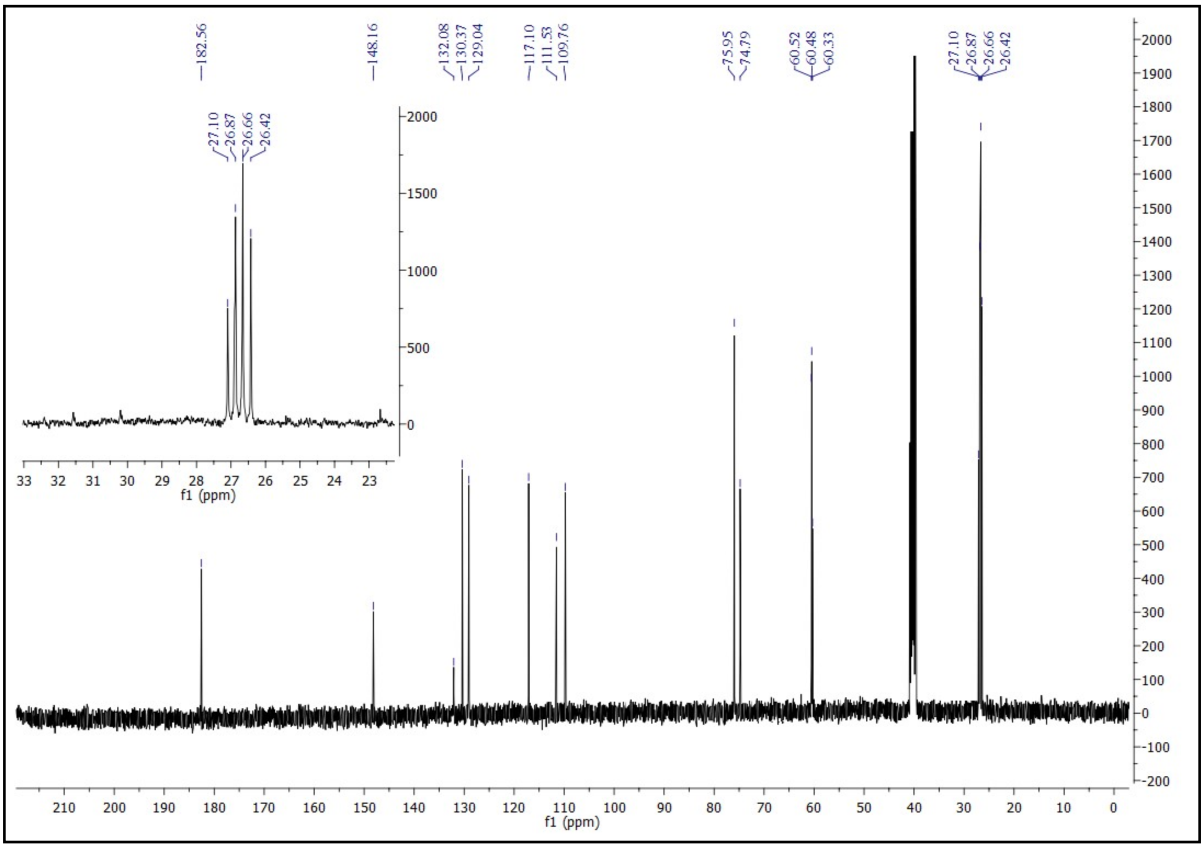

Figure S8: ^{1}H NMR and ^{13}C NMR of **2h**.

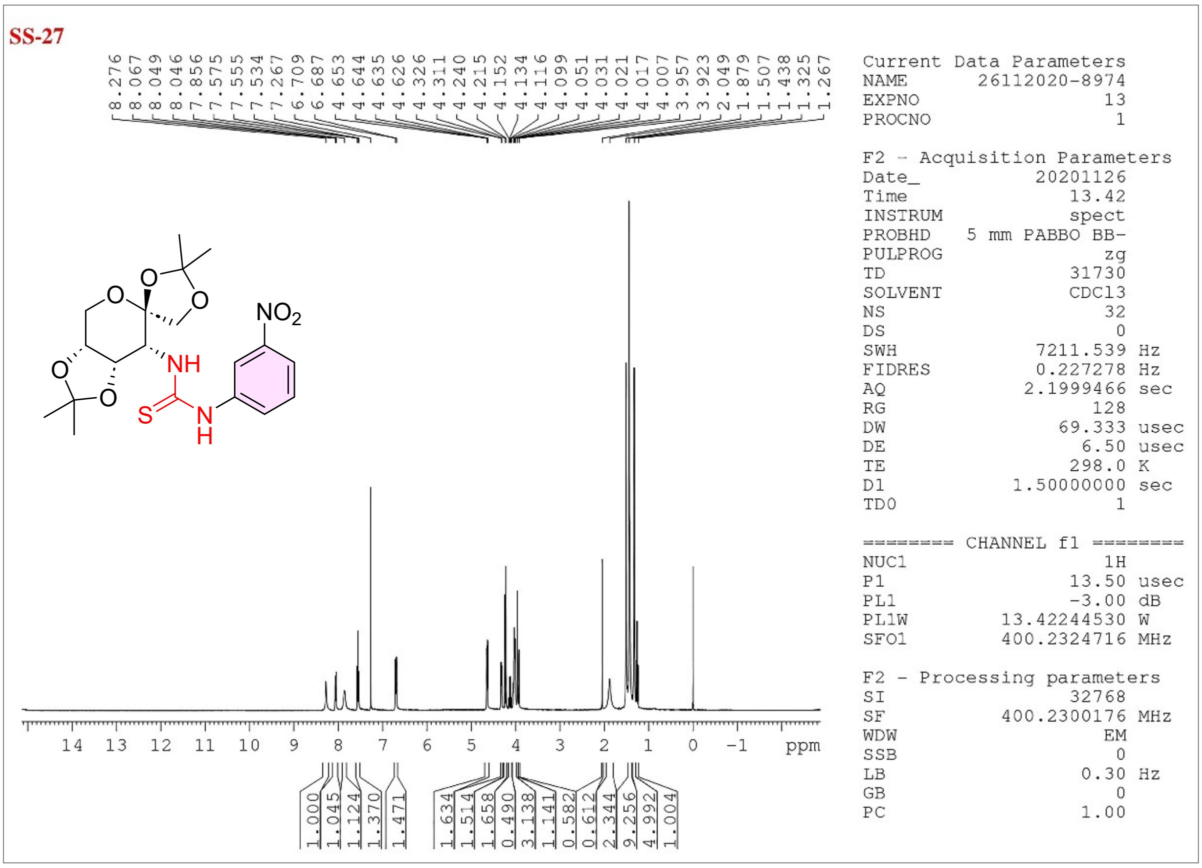

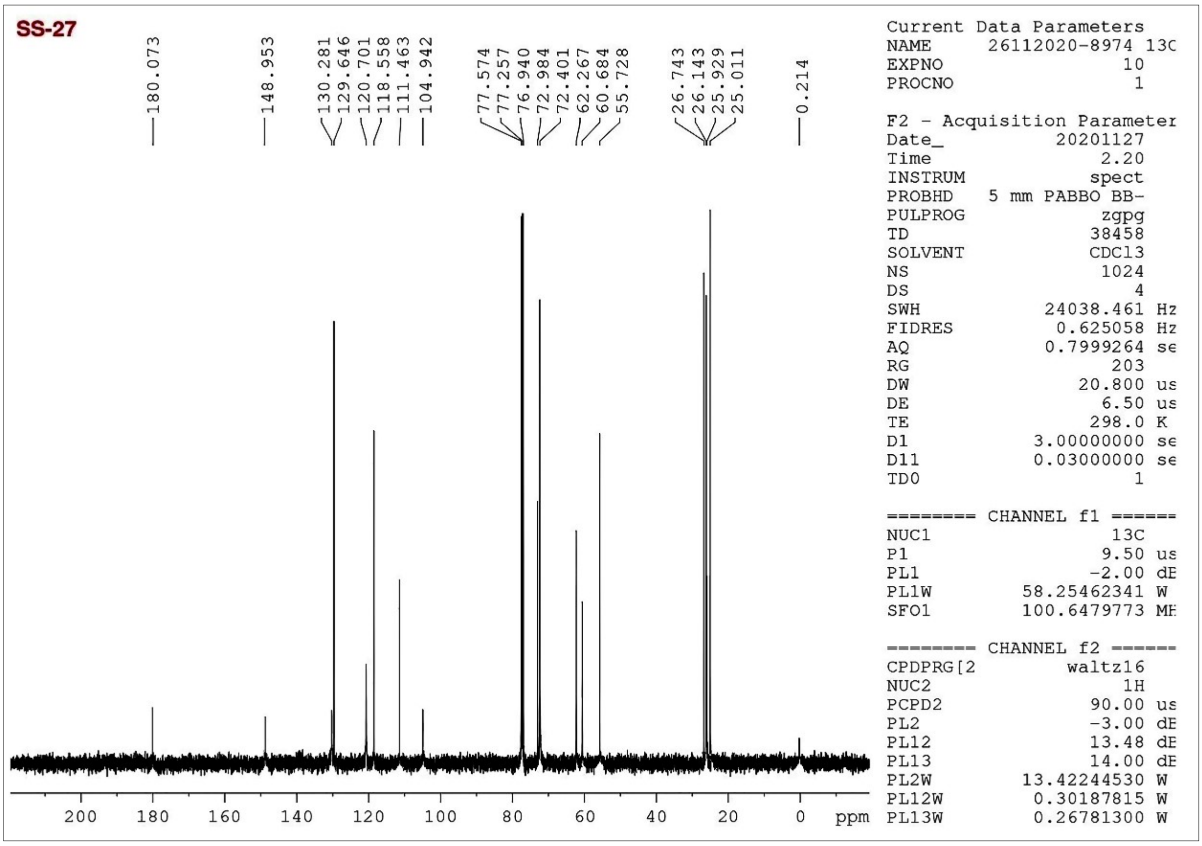

Figure S9: ^{1}H NMR and ^{13}C NMR of **3a**.

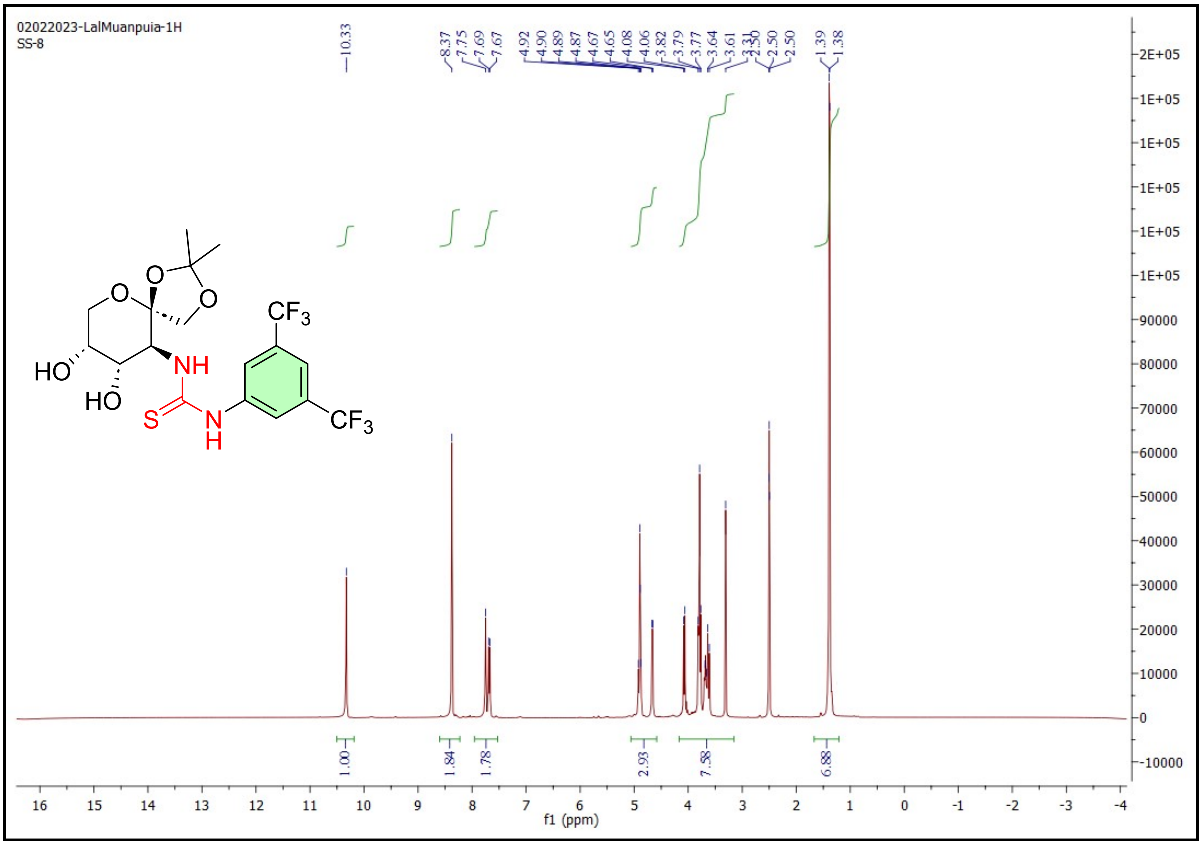

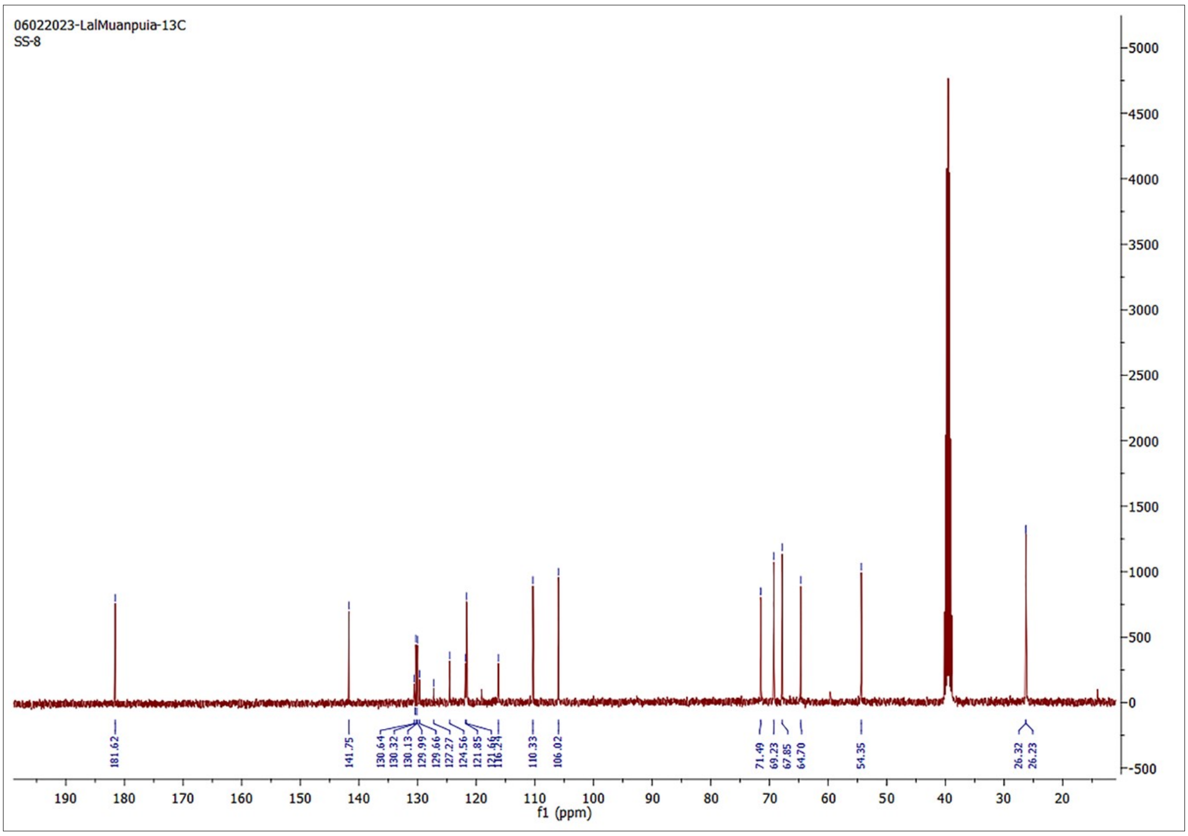

Figure S10: ^{1}H NMR and ^{13}C NMR of **3b**.

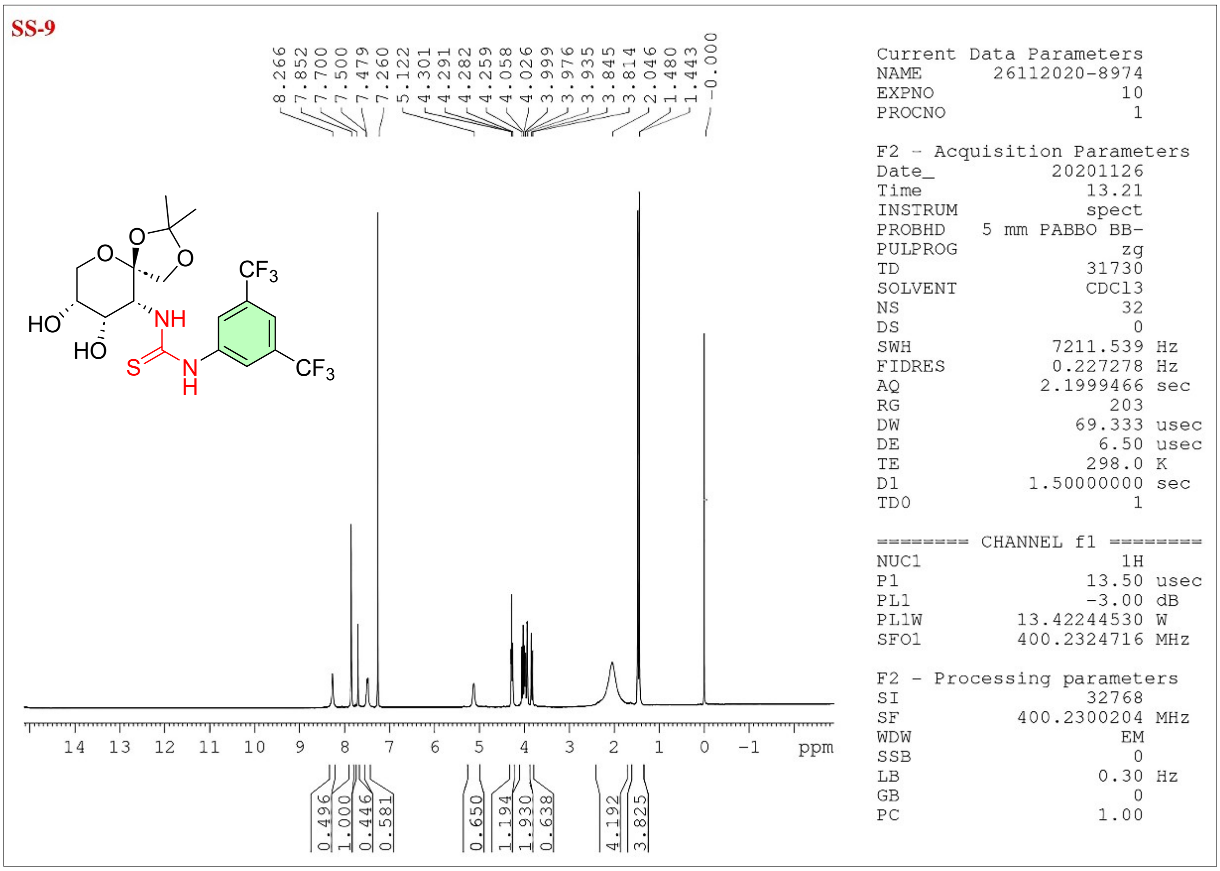

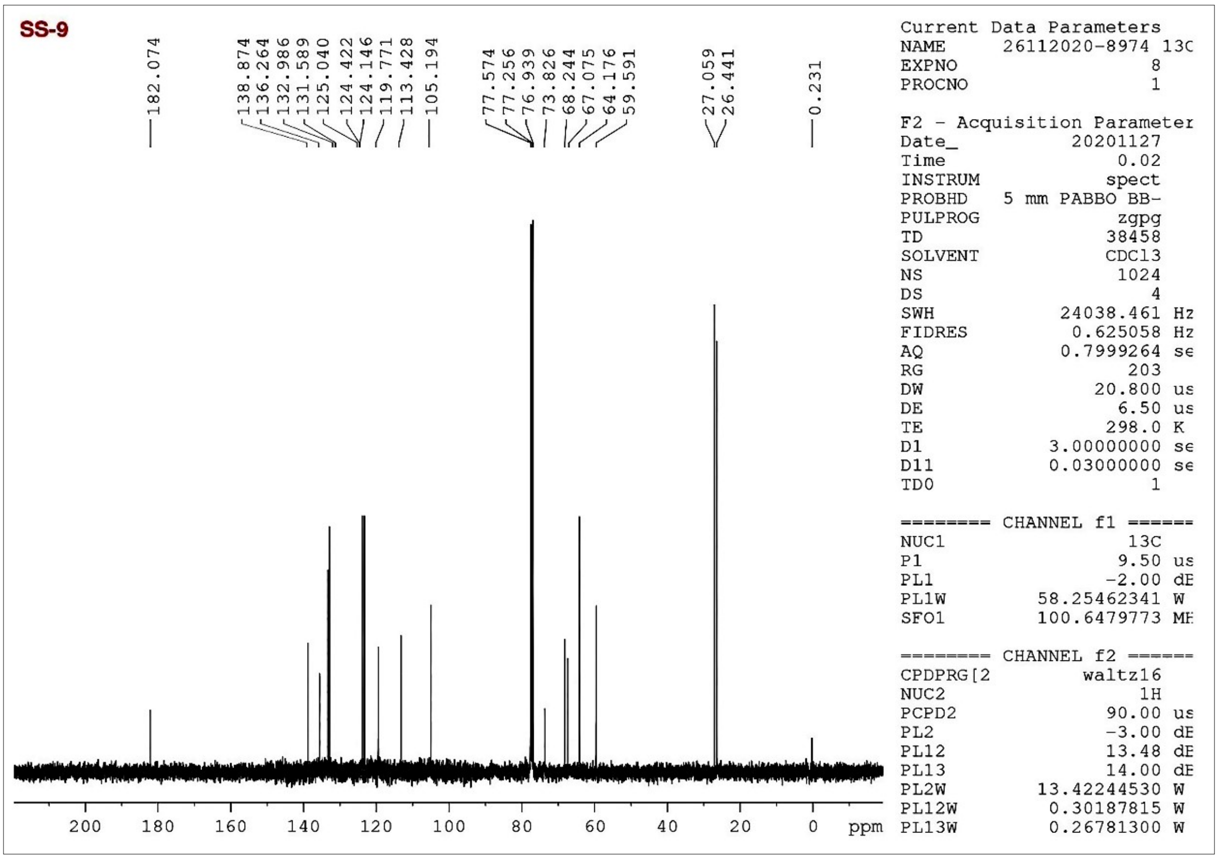

Figure S11: ^{1}H NMR and ^{13}C NMR of **3c.**

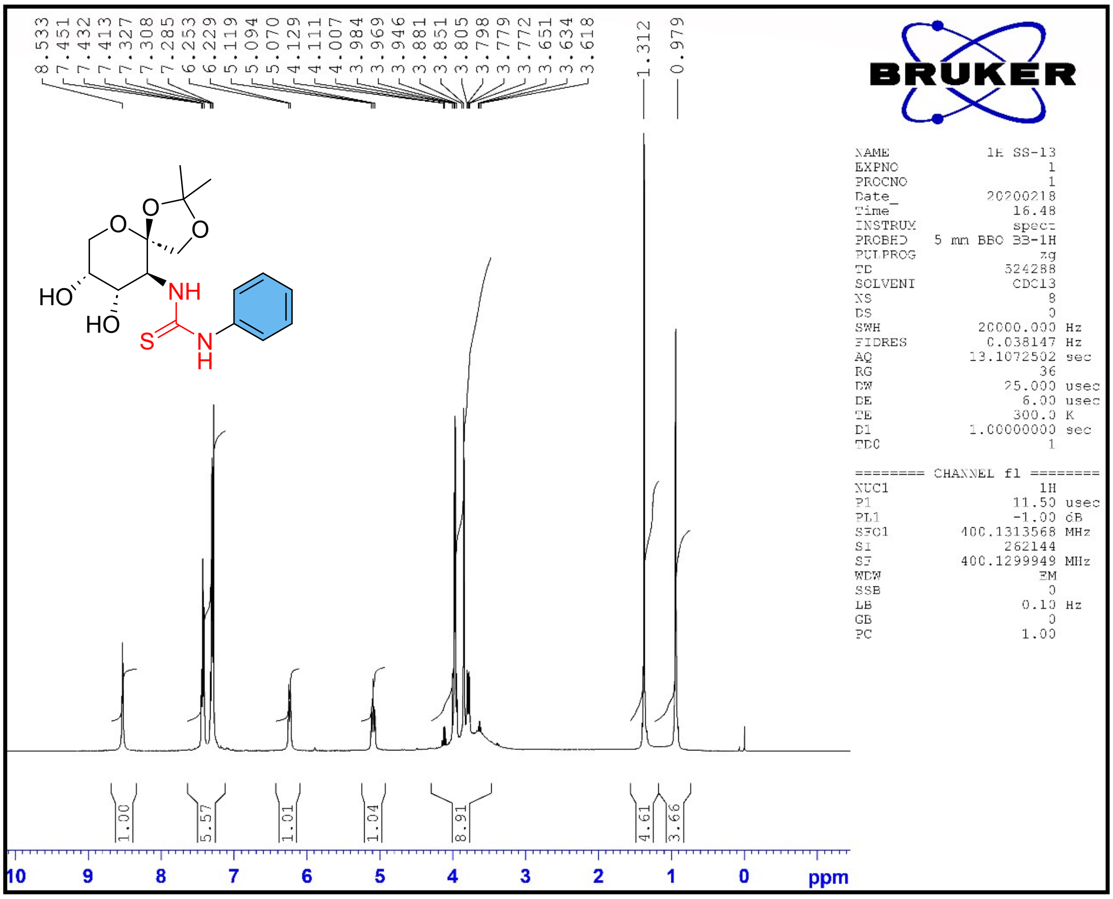

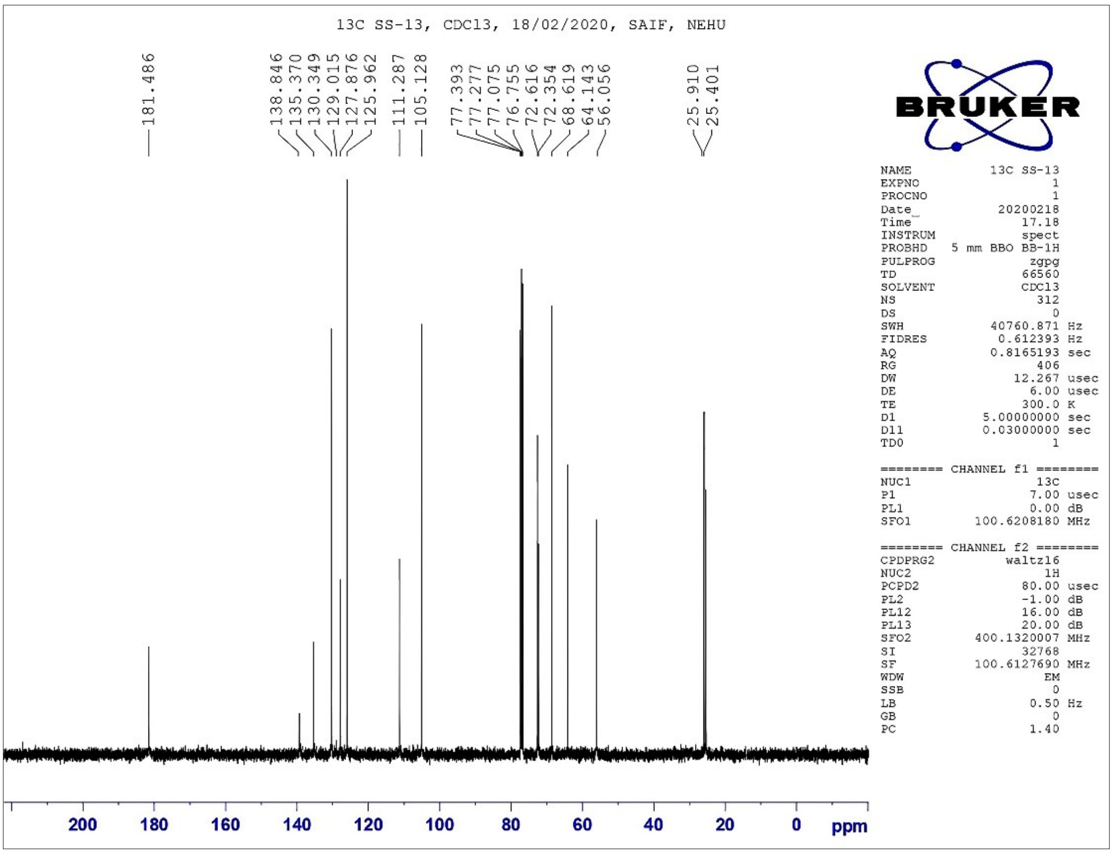

Figure S12: ^{1}H NMR and ^{13}C NMR of **3d.**

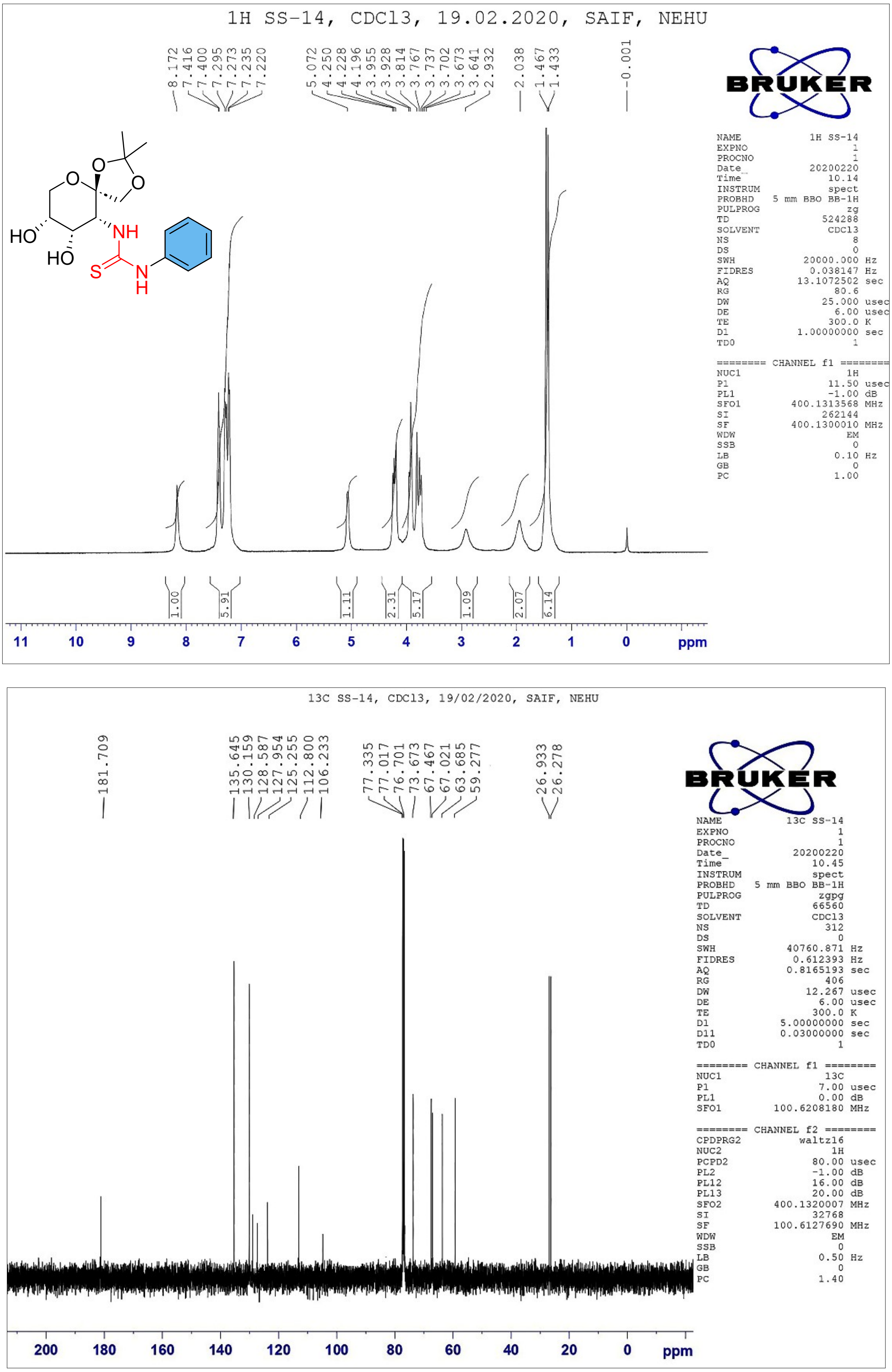

Figure S13: ^{1}H NMR and ^{13}C NMR of **3e.**

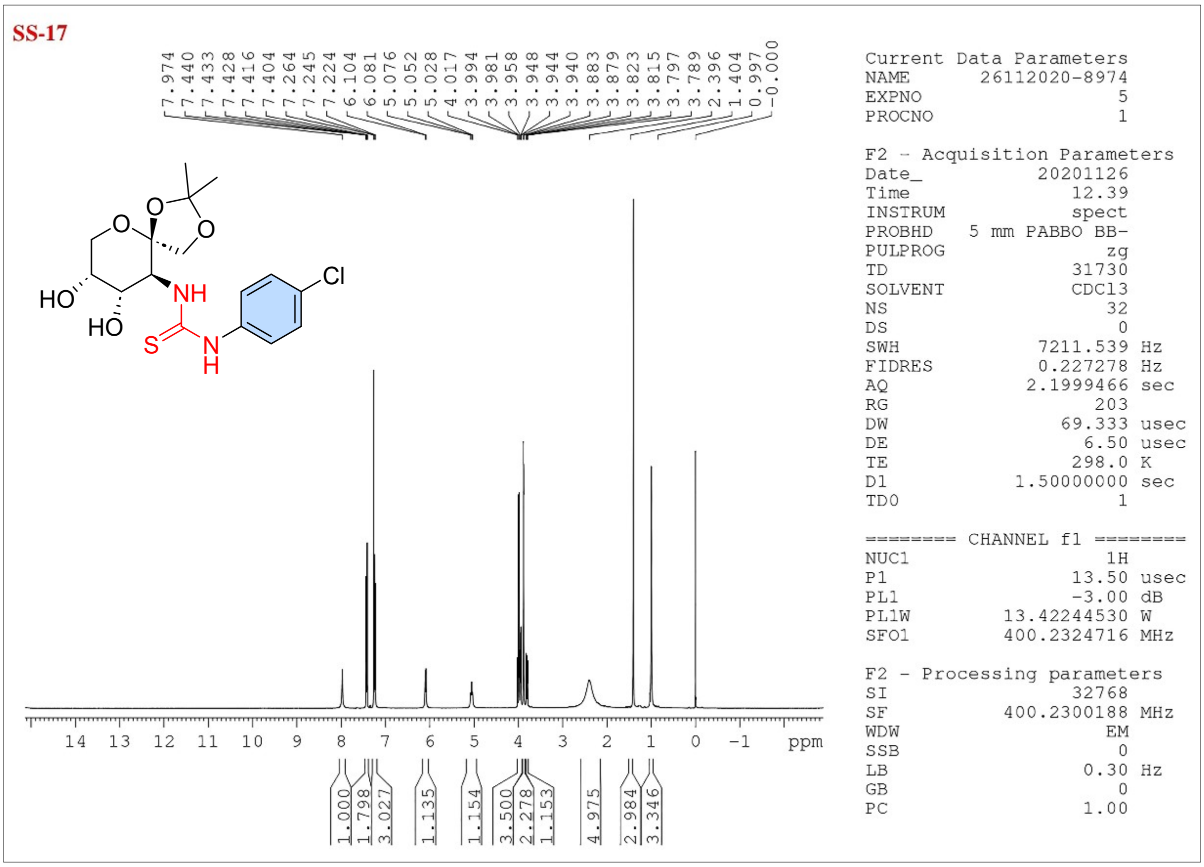

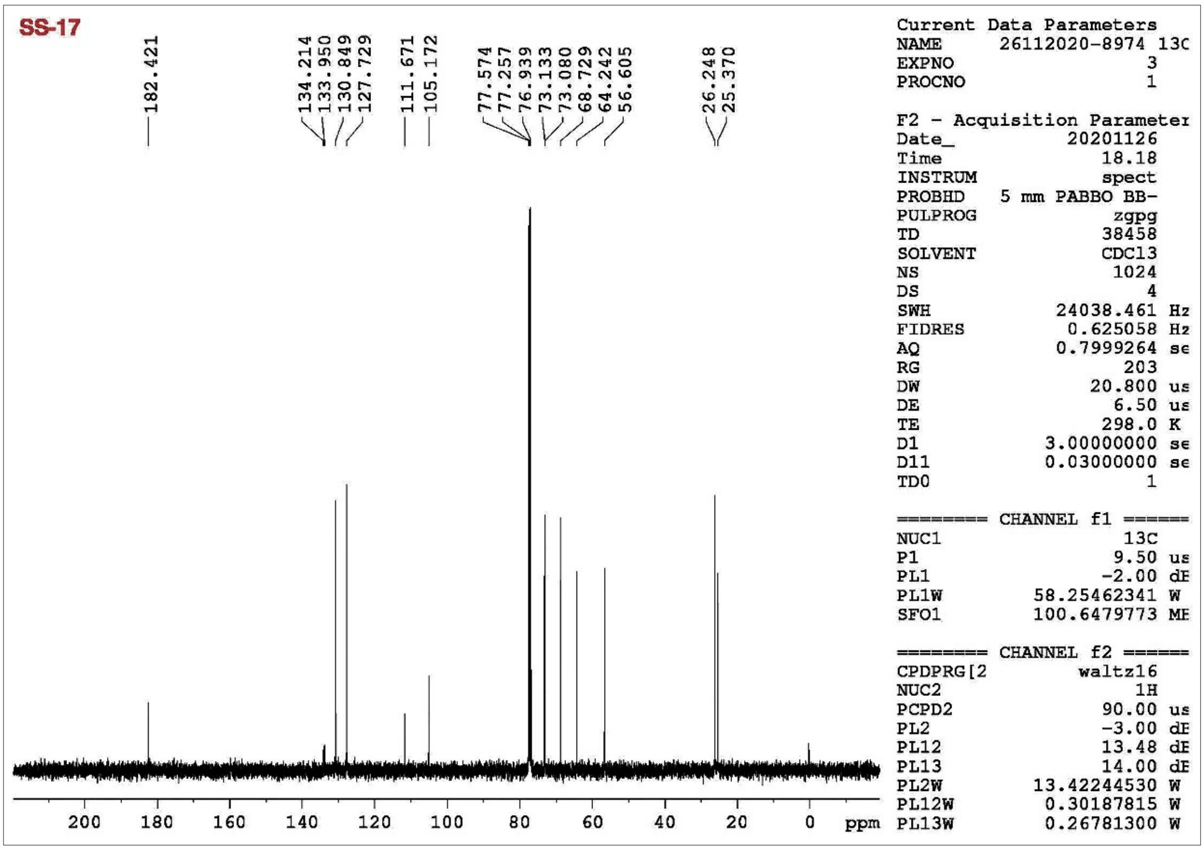

Figure S14: ^{1}H NMR and ^{13}C NMR of **3f.**

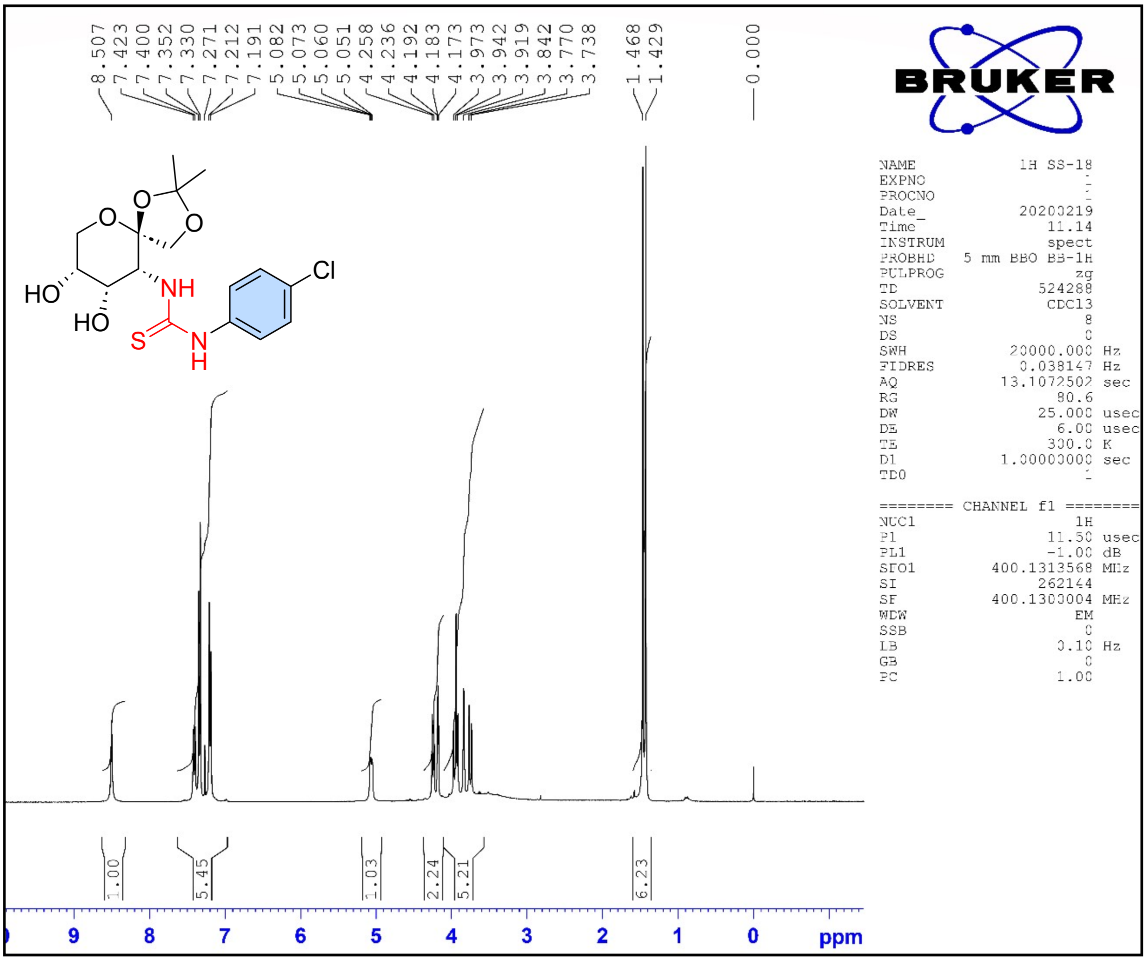

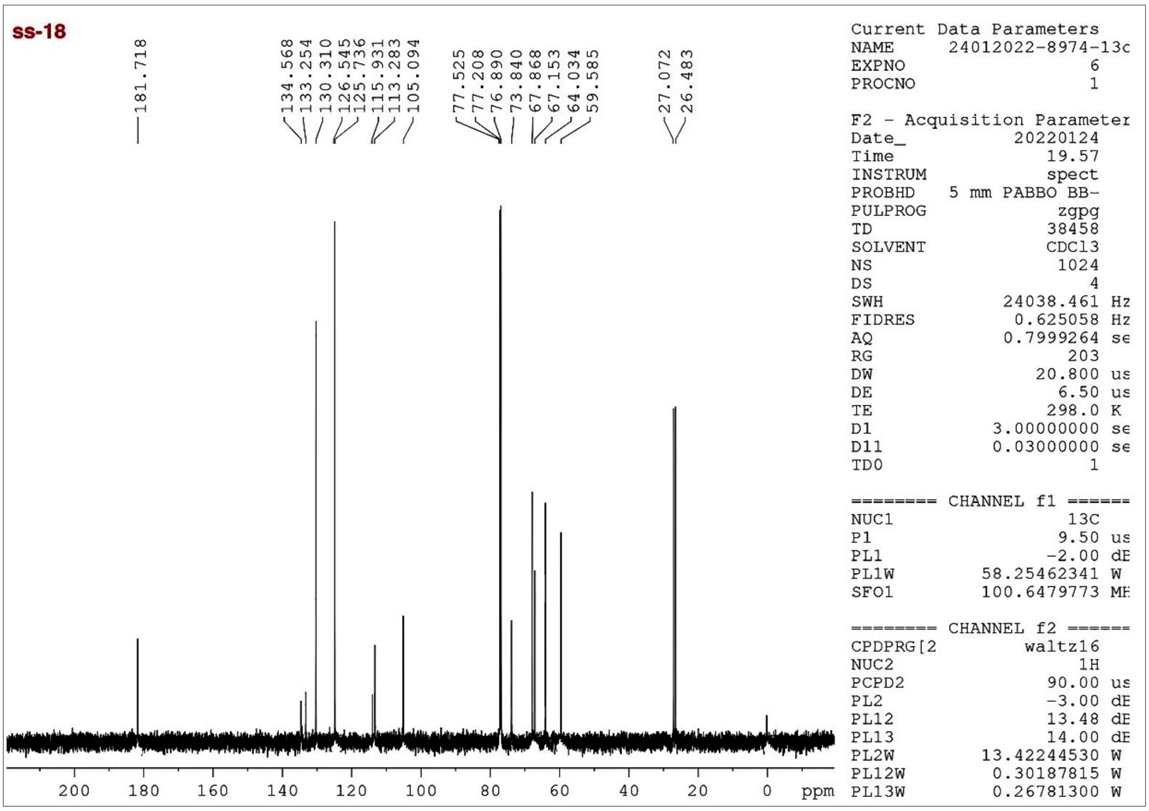

Figure S15: ^{1}H NMR and ^{13}C NMR of **3g.**

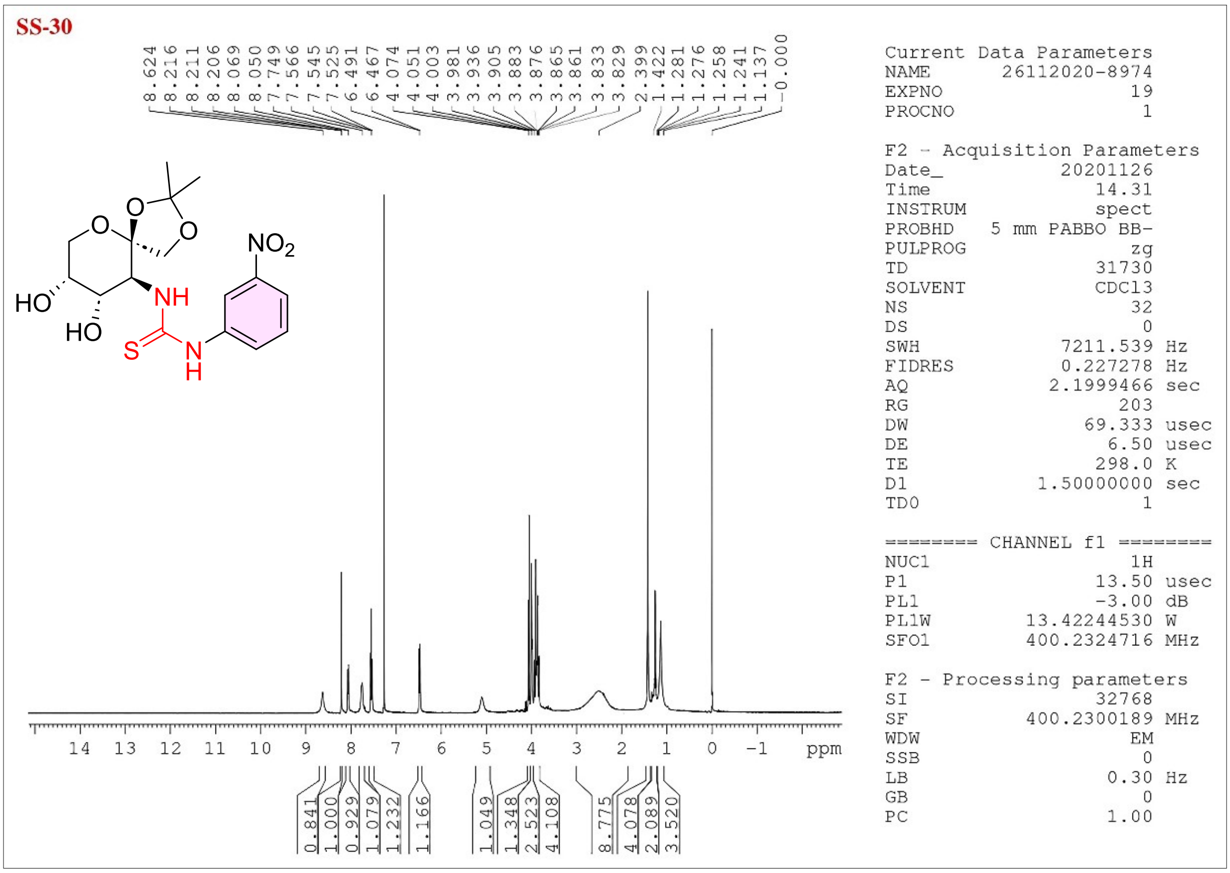

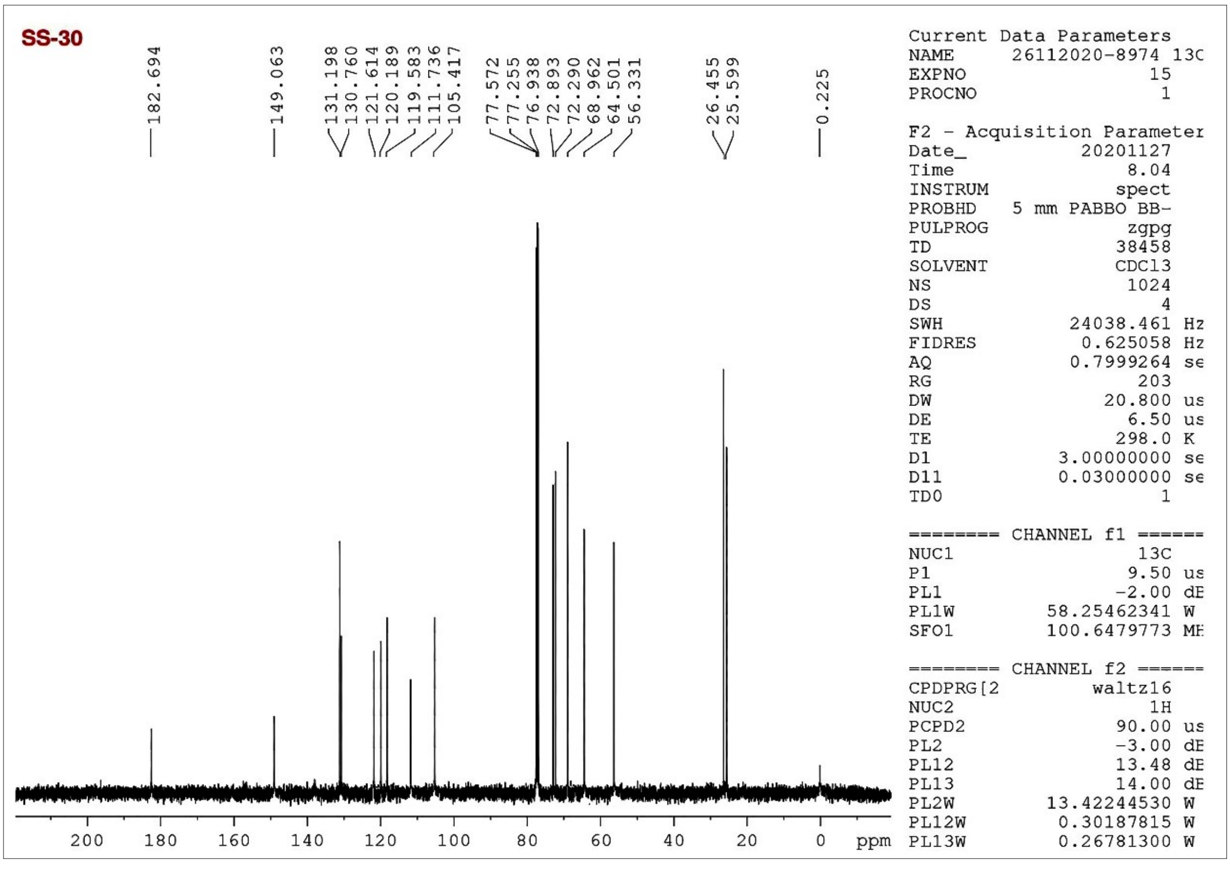

Figure S16: ^{1}H NMR and ^{13}C NMR of **3h.**

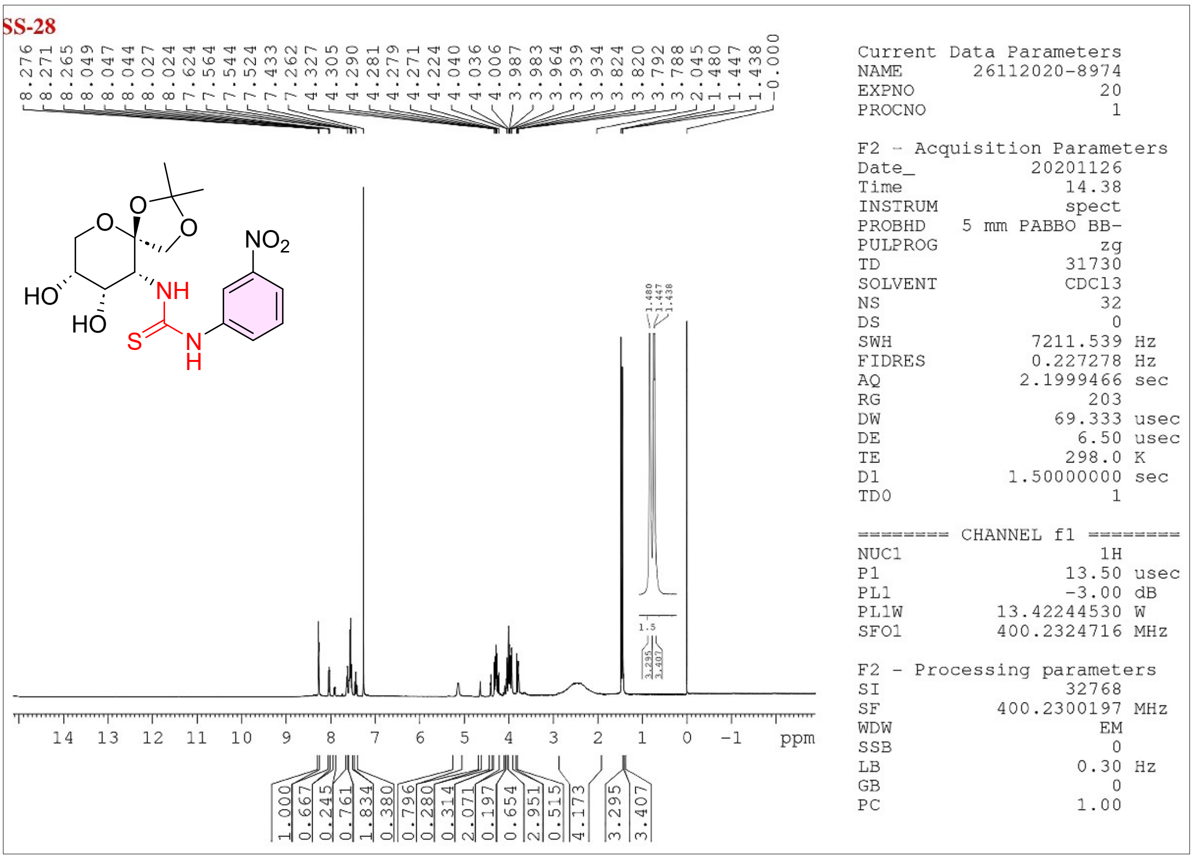

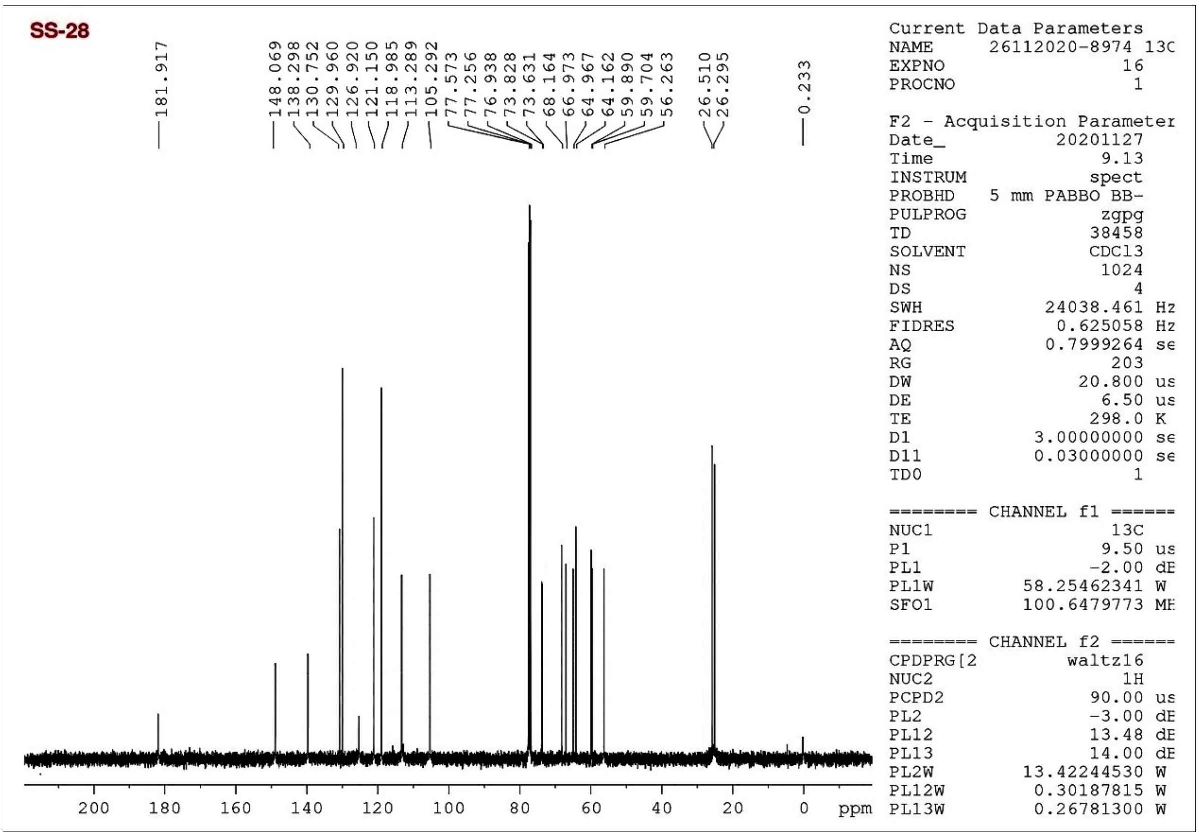

Figure S17: ^{1}H NMR and ^{13}C NMR of 3-(2-Nitro-1-4-chlorophenyl-ethyl)-1H-indole **(4a).**

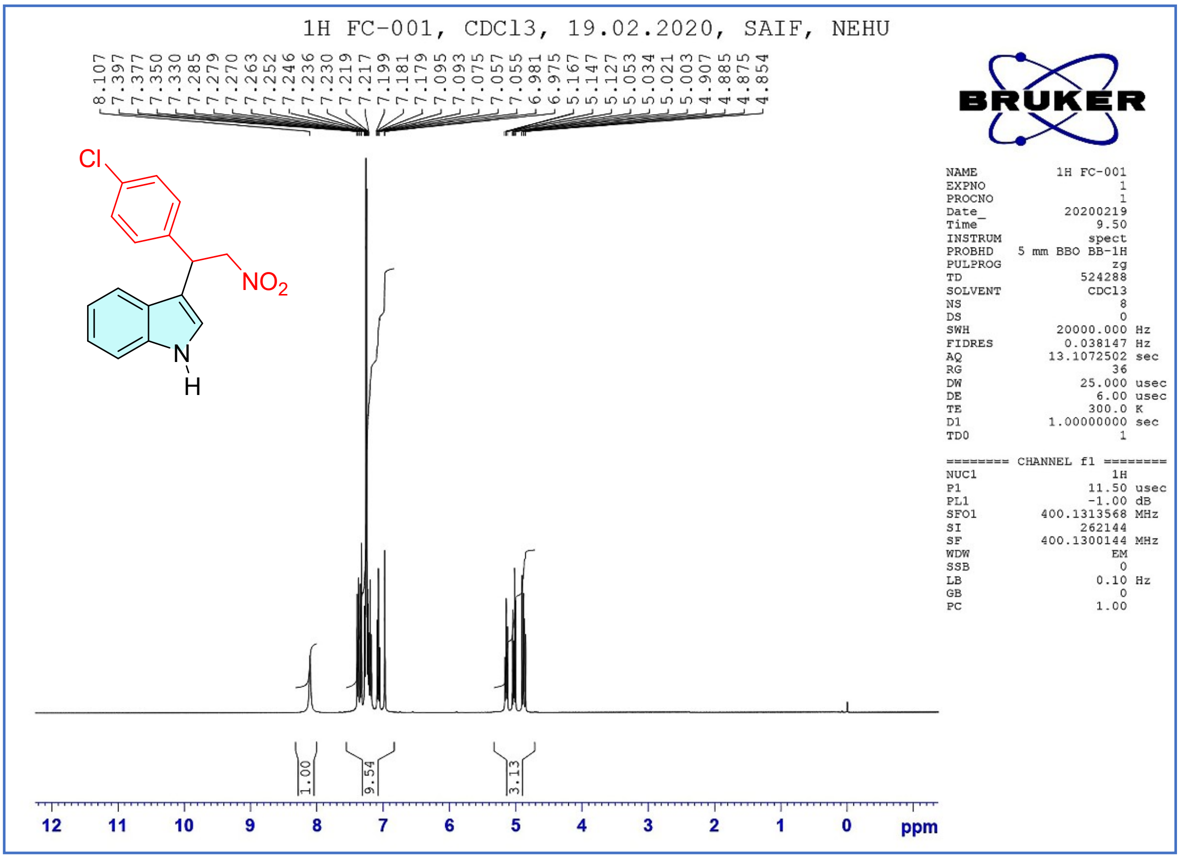

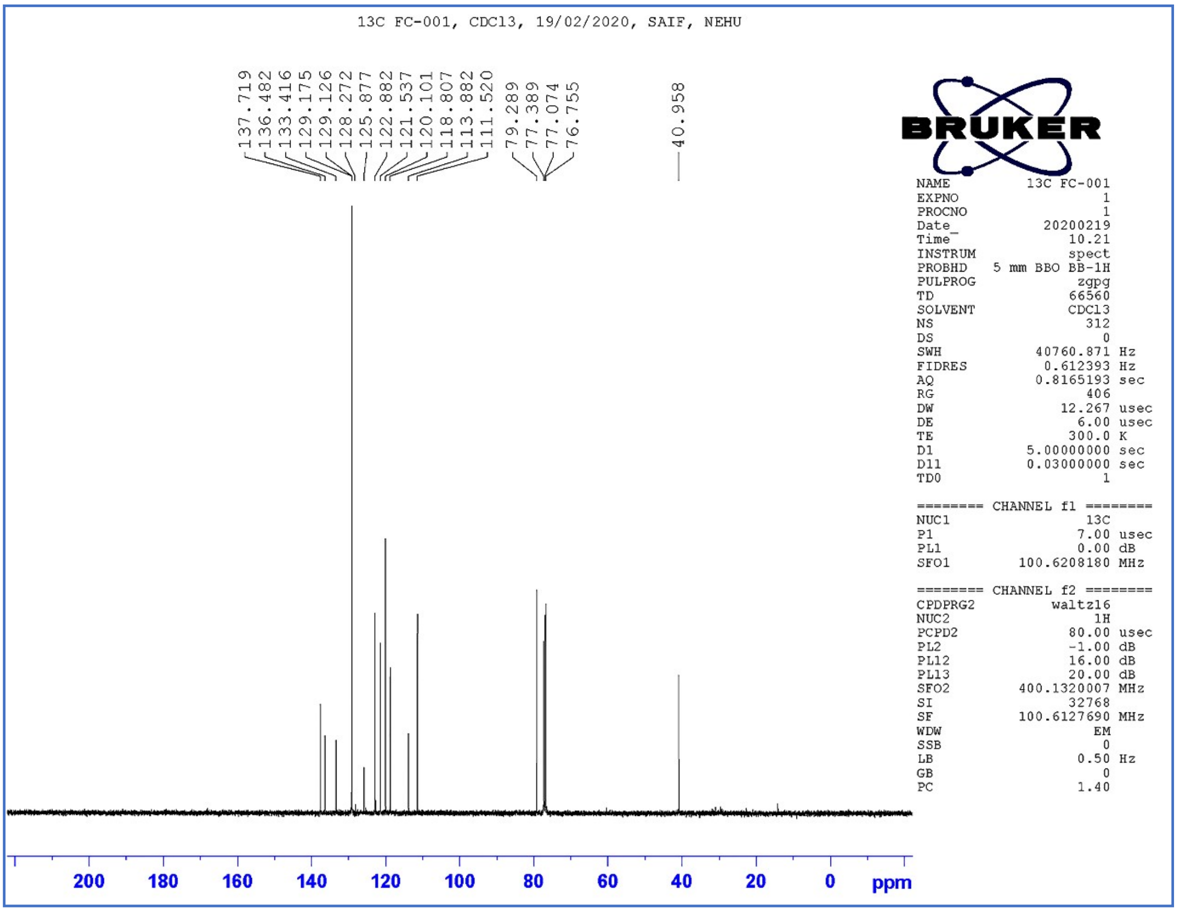

Figure S18: ^{1}H NMR and ^{13}C NMR of 3-(2-Nitro-1-4-bromophenyl-ethyl)-1H-indole **(4b).**

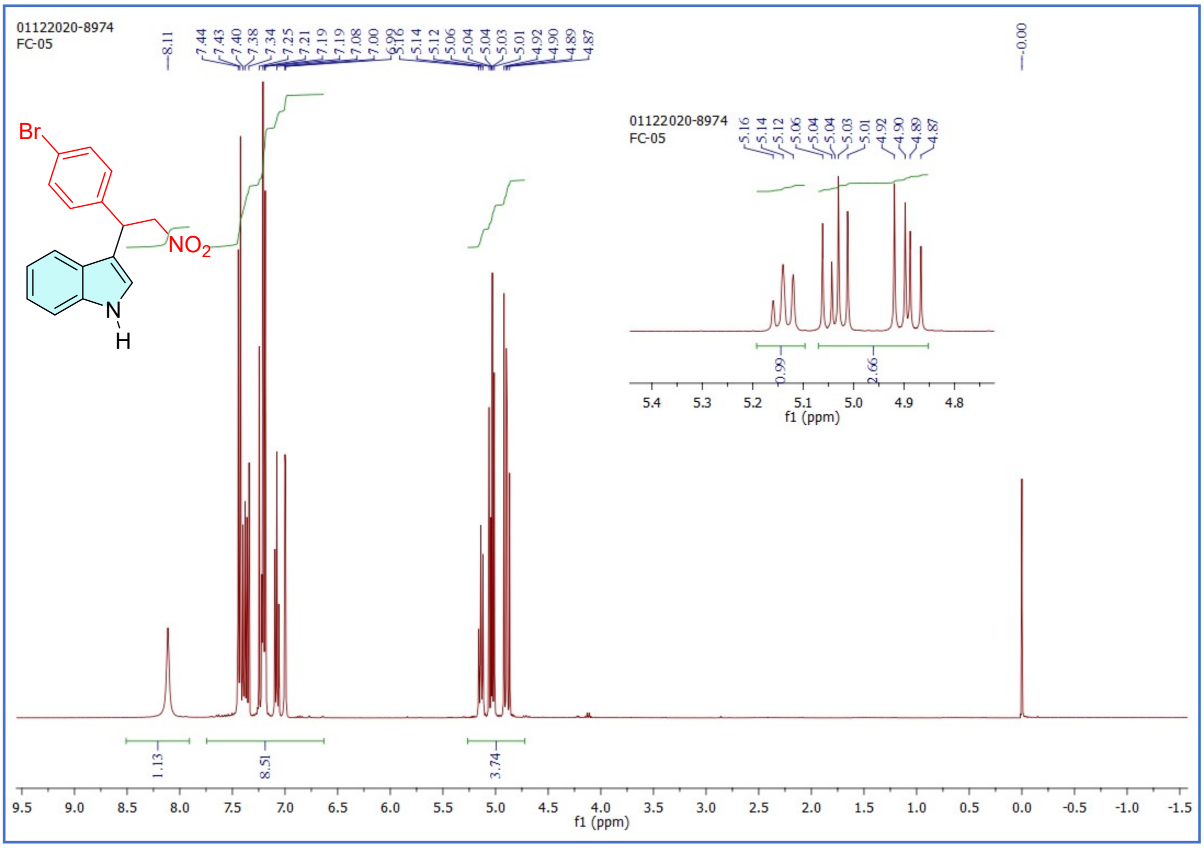

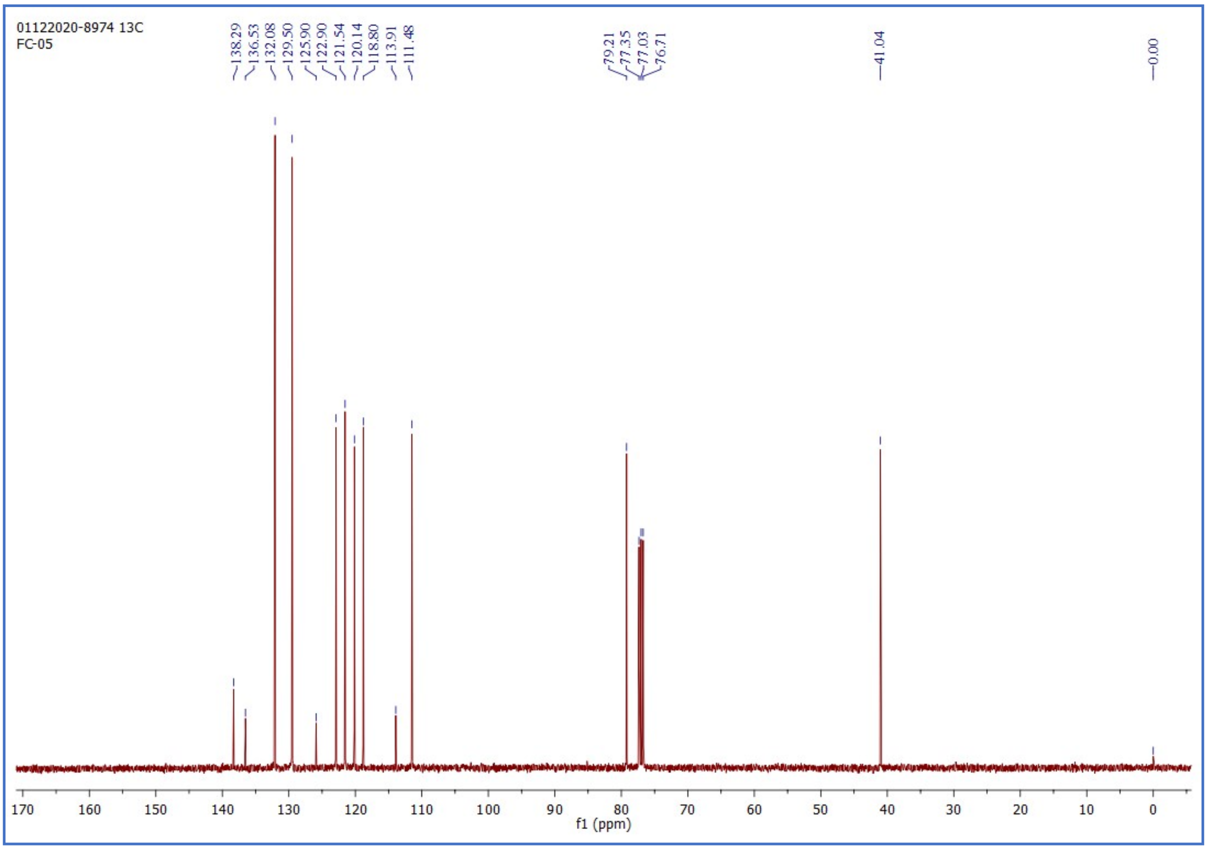

Figure S19: ^{1}H NMR and ^{13}C NMR of 3-(2-Nitro-1-4-methylphenyl-ethyl)-1H-indole **(4c).**

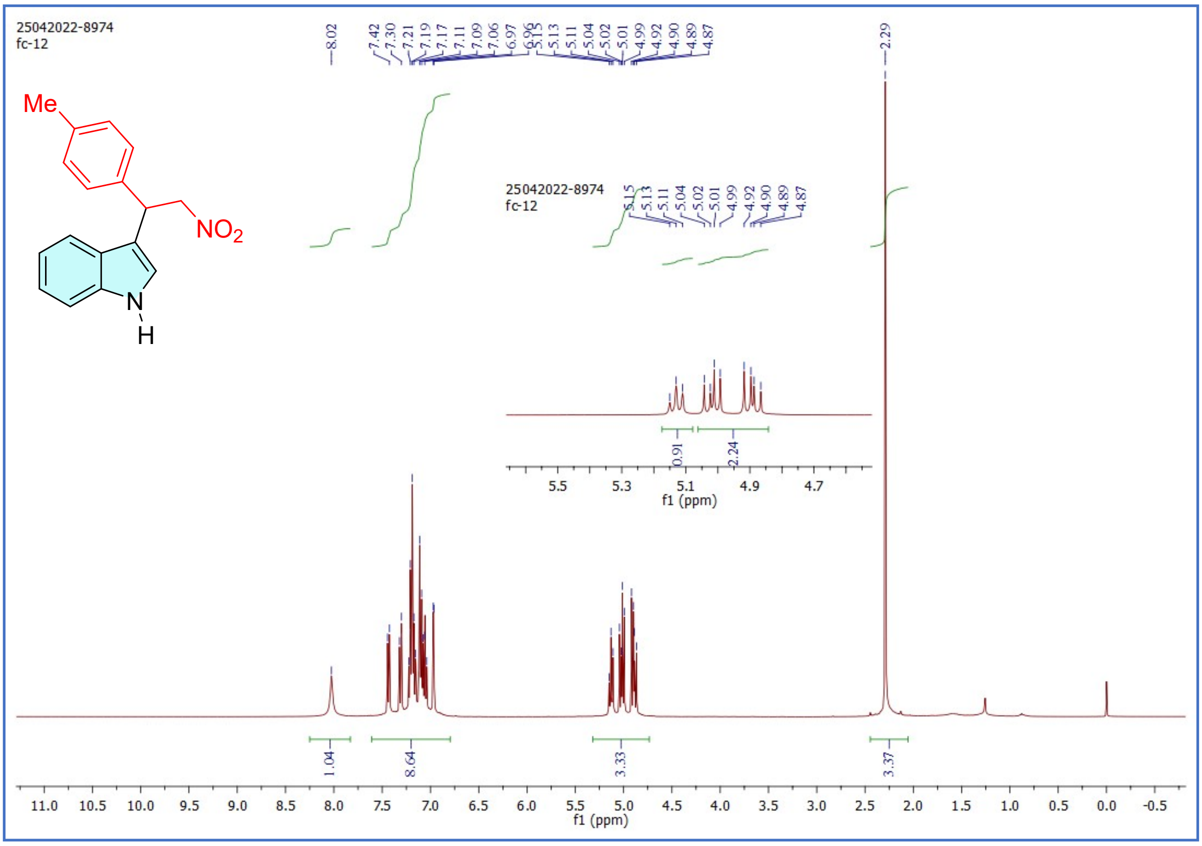

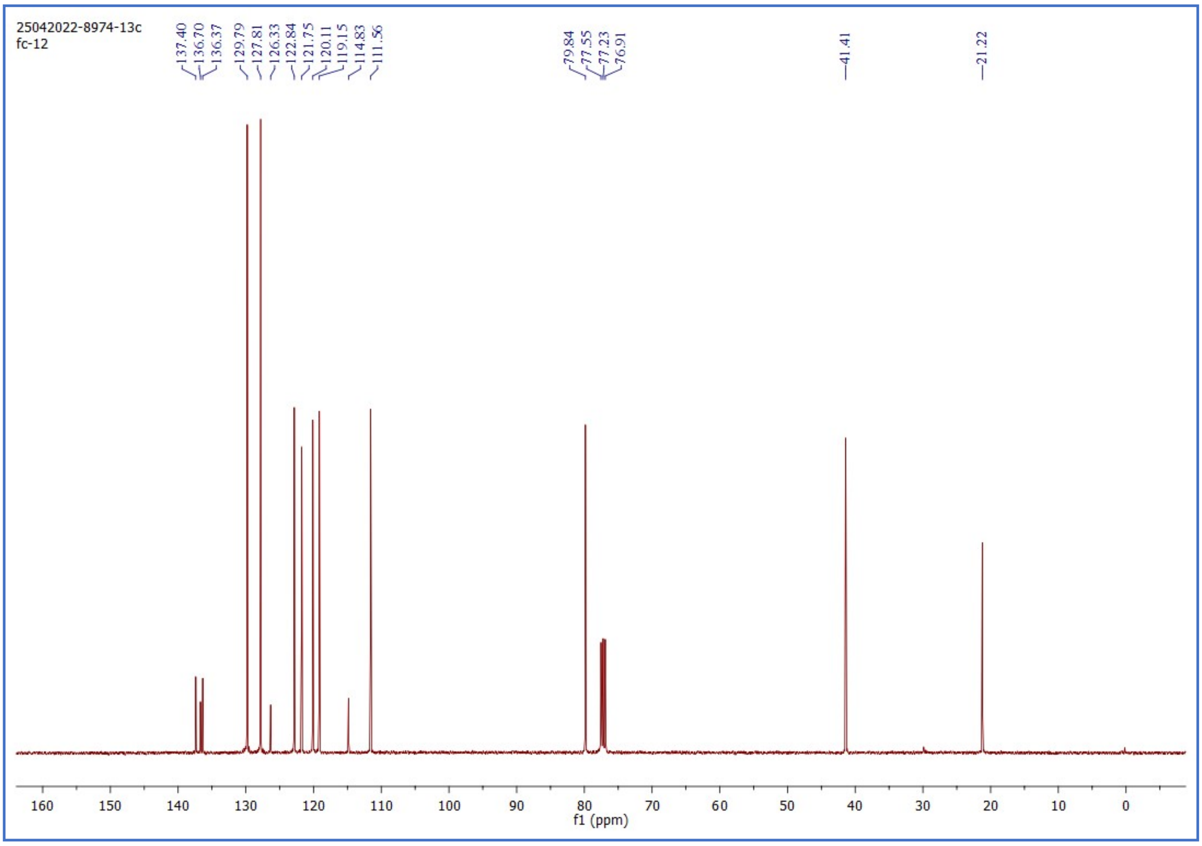

Figure S20: ^{1}H NMR and ^{13}C NMR of 3-(2-Nitro-1-2-chlorophenyl-ethyl)-1H-indole **(4d)**.

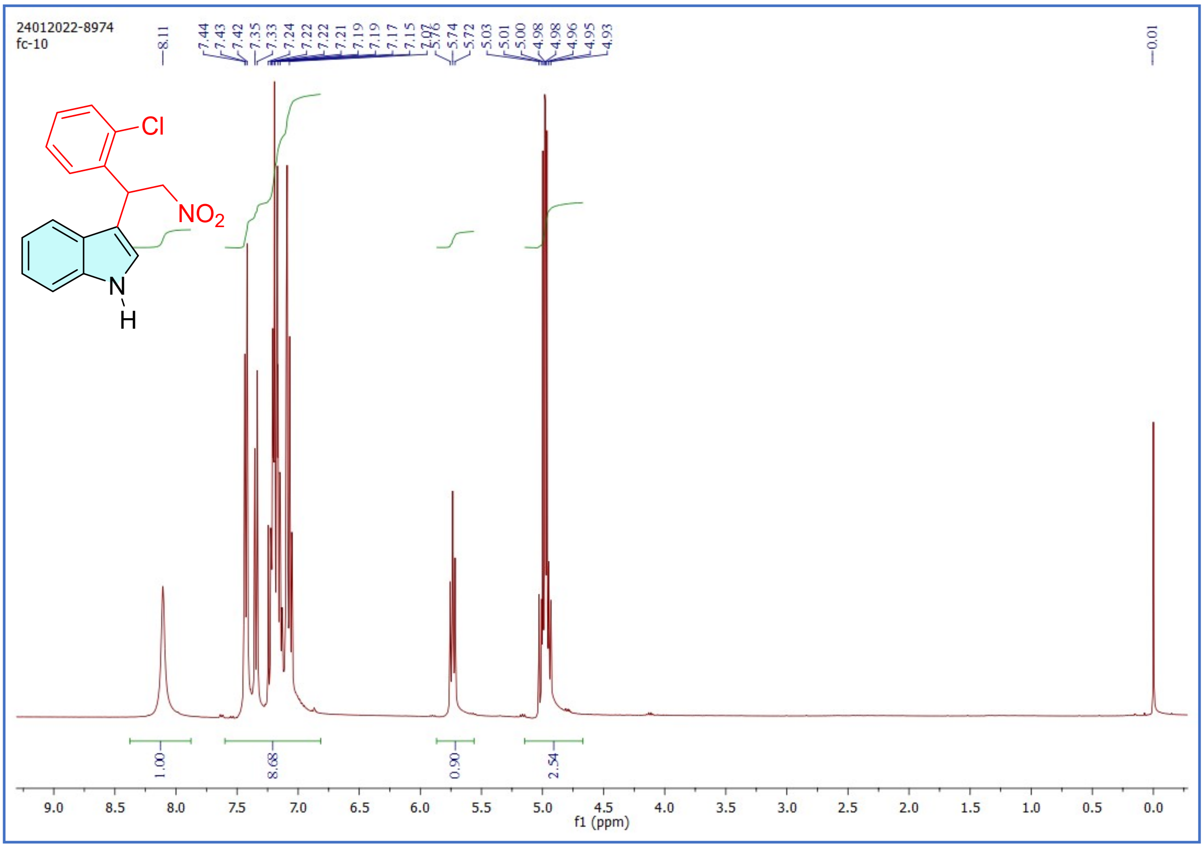

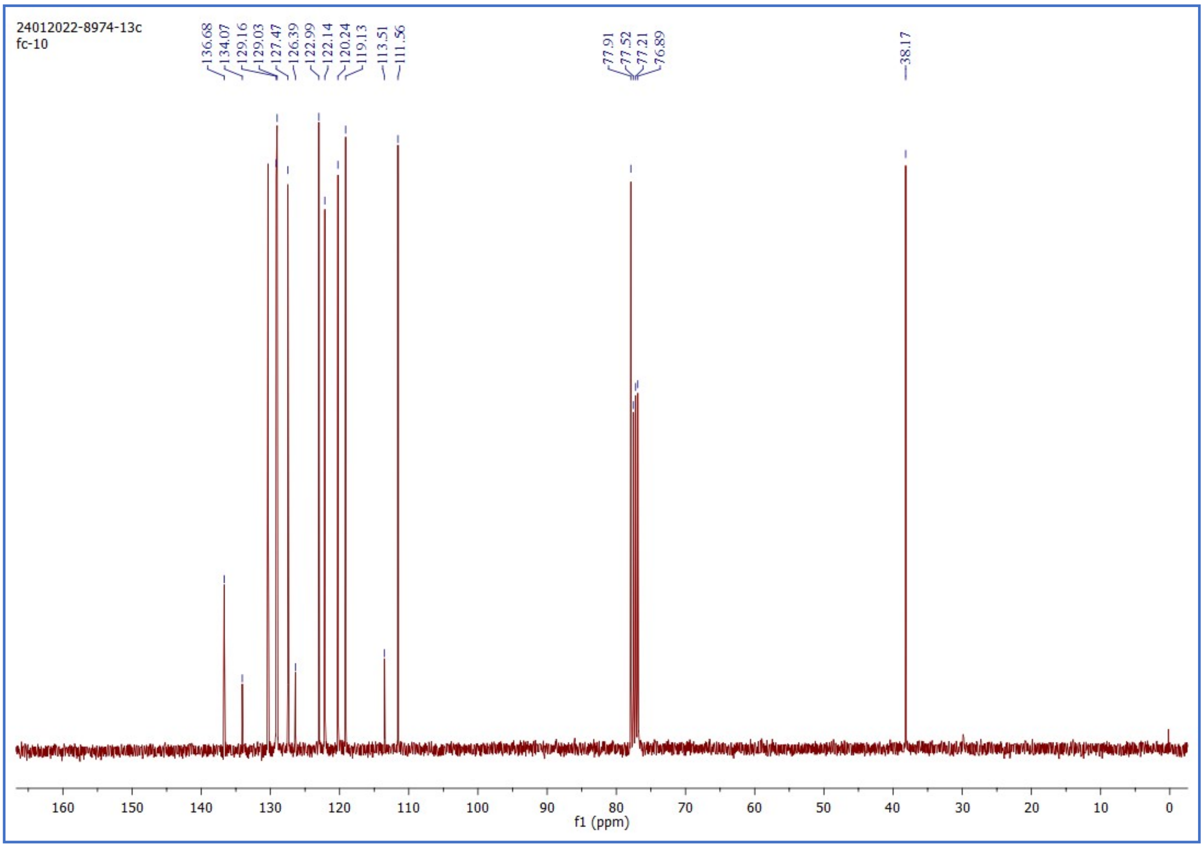

Figure S21: ^{1}H NMR and ^{13}C NMR of 3-(2-Nitro-1-2-hydroxyophenyl-ethyl)-1H-indole **(4e)**.

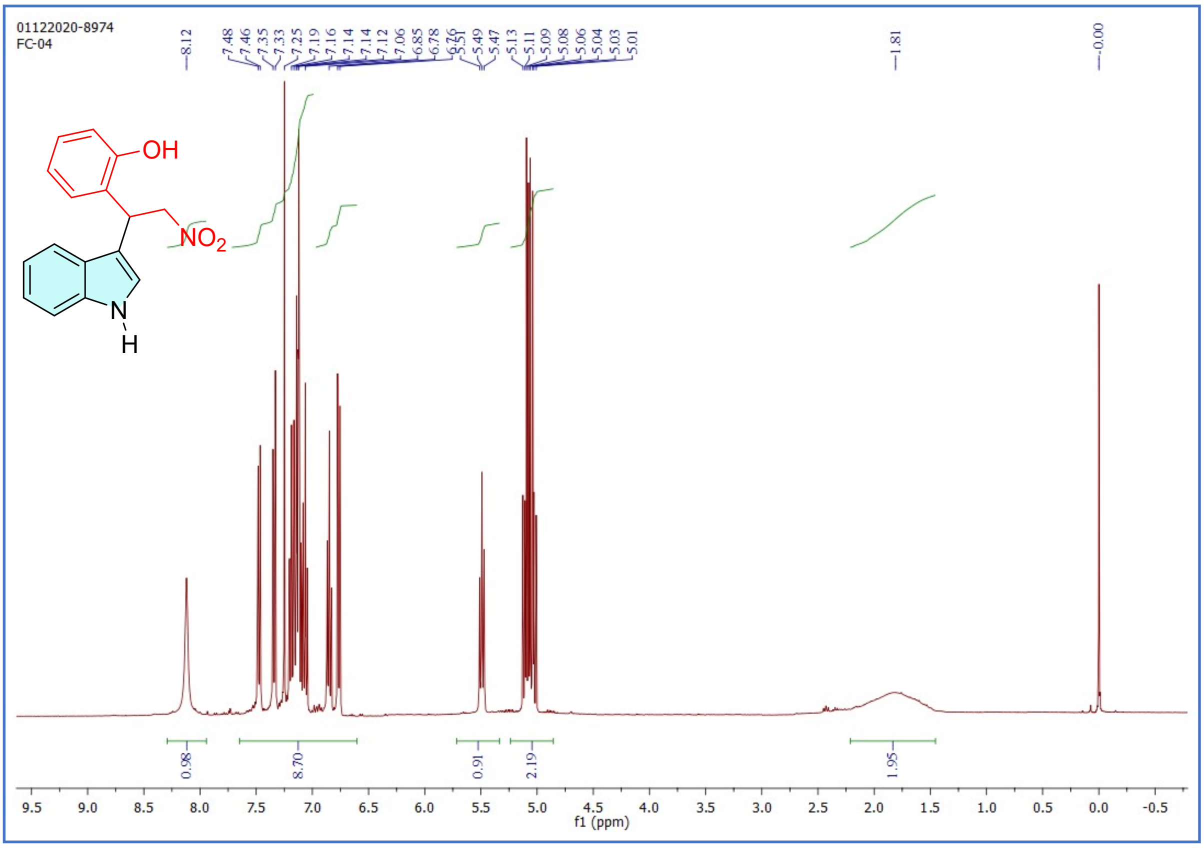

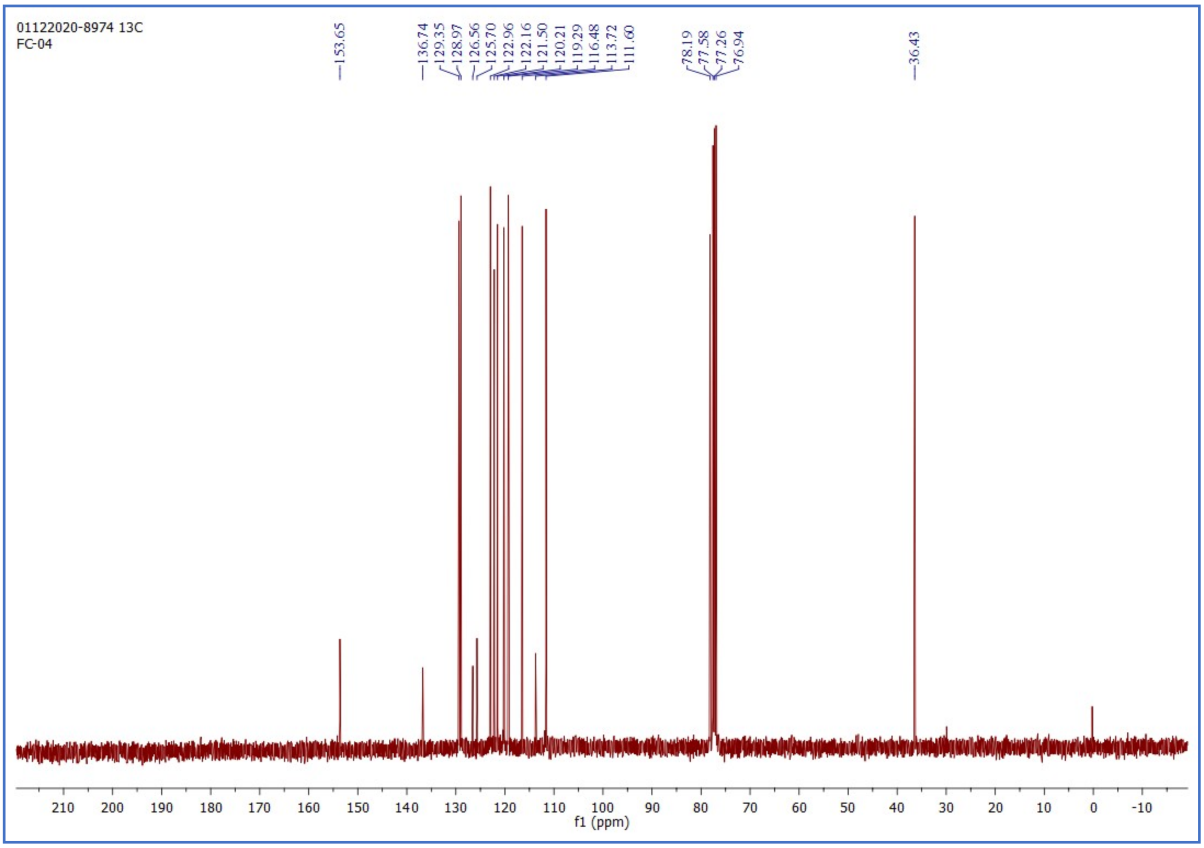

Figure S22: ^{1}H NMR and ^{13}C NMR of 3-(2-Nitro-1-2-methoxyphenyl-ethyl)-1H-indole **(4f)**.

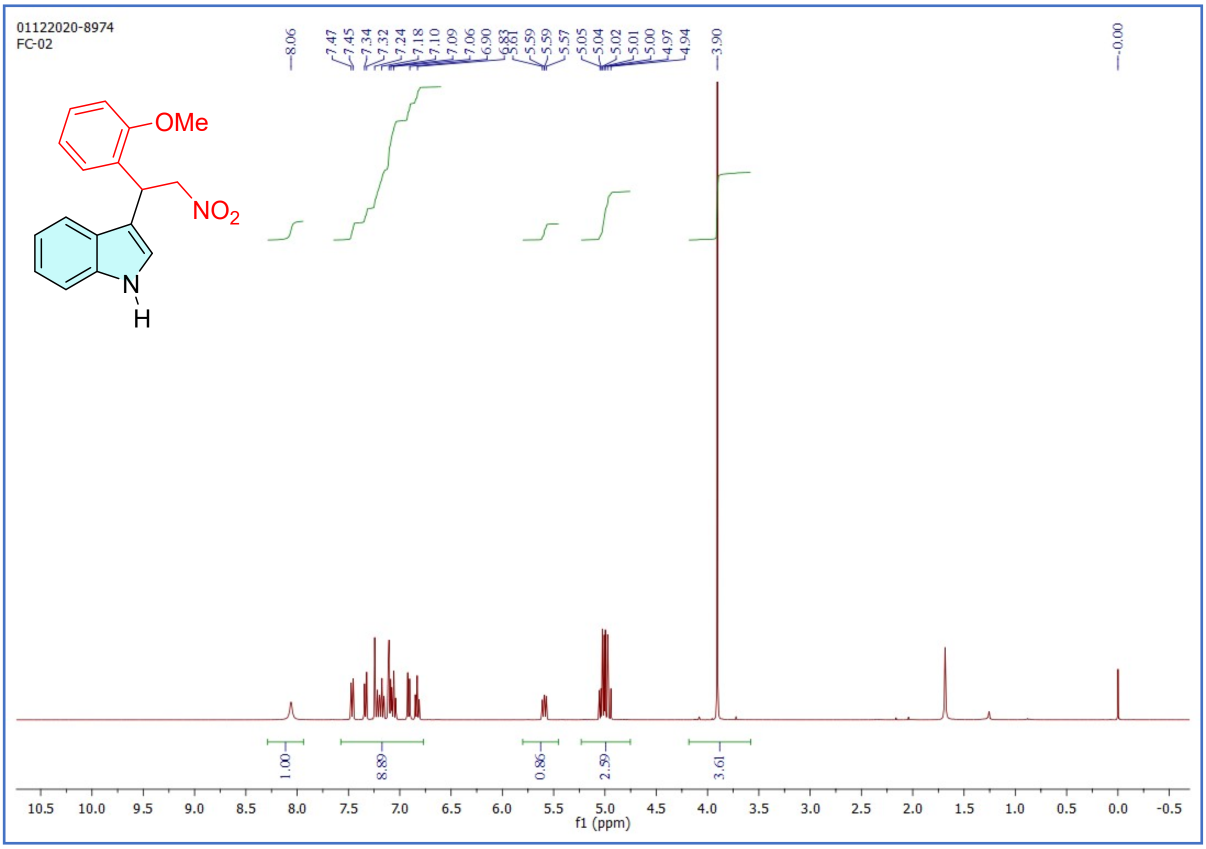

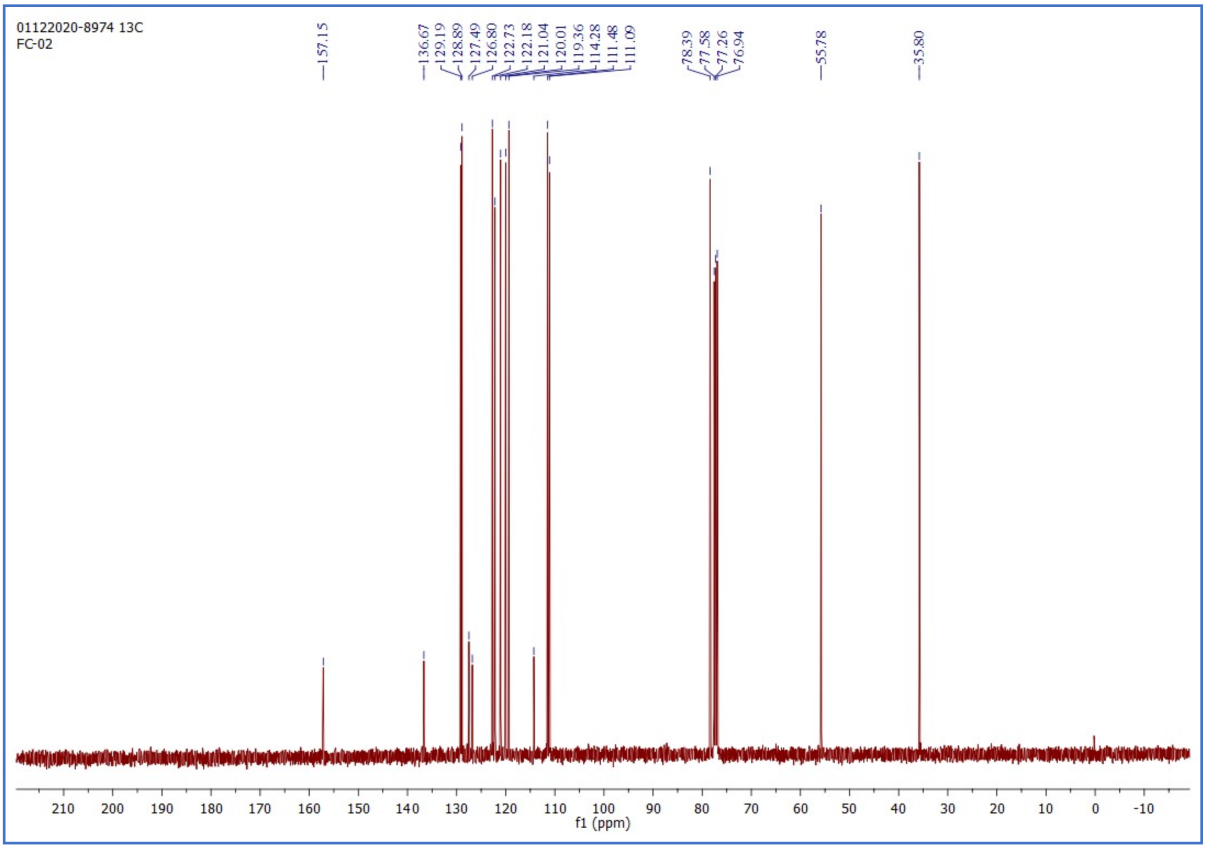

Figure S23: ^{1}H NMR and ^{13}C NMR of 3-(2-nitro-1-phenylethyl)-1H-indole **(4g).**

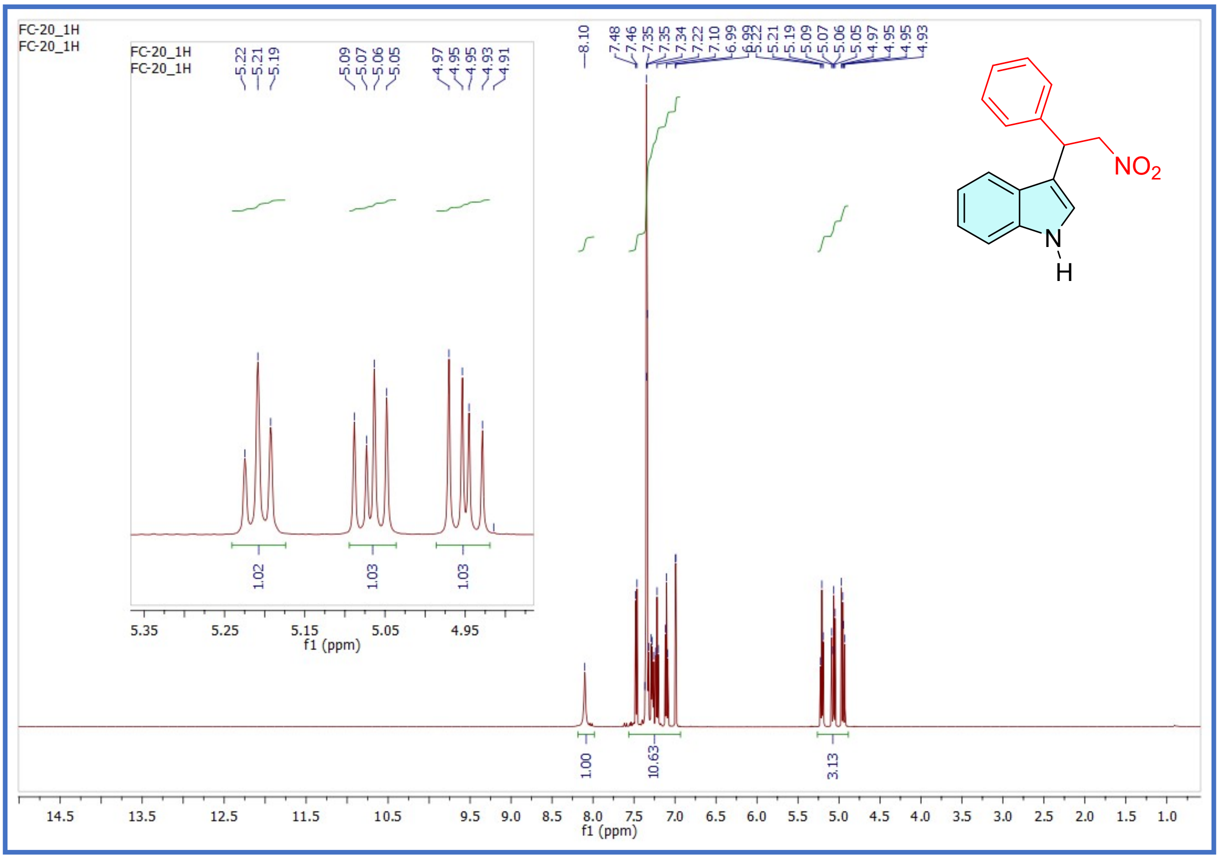

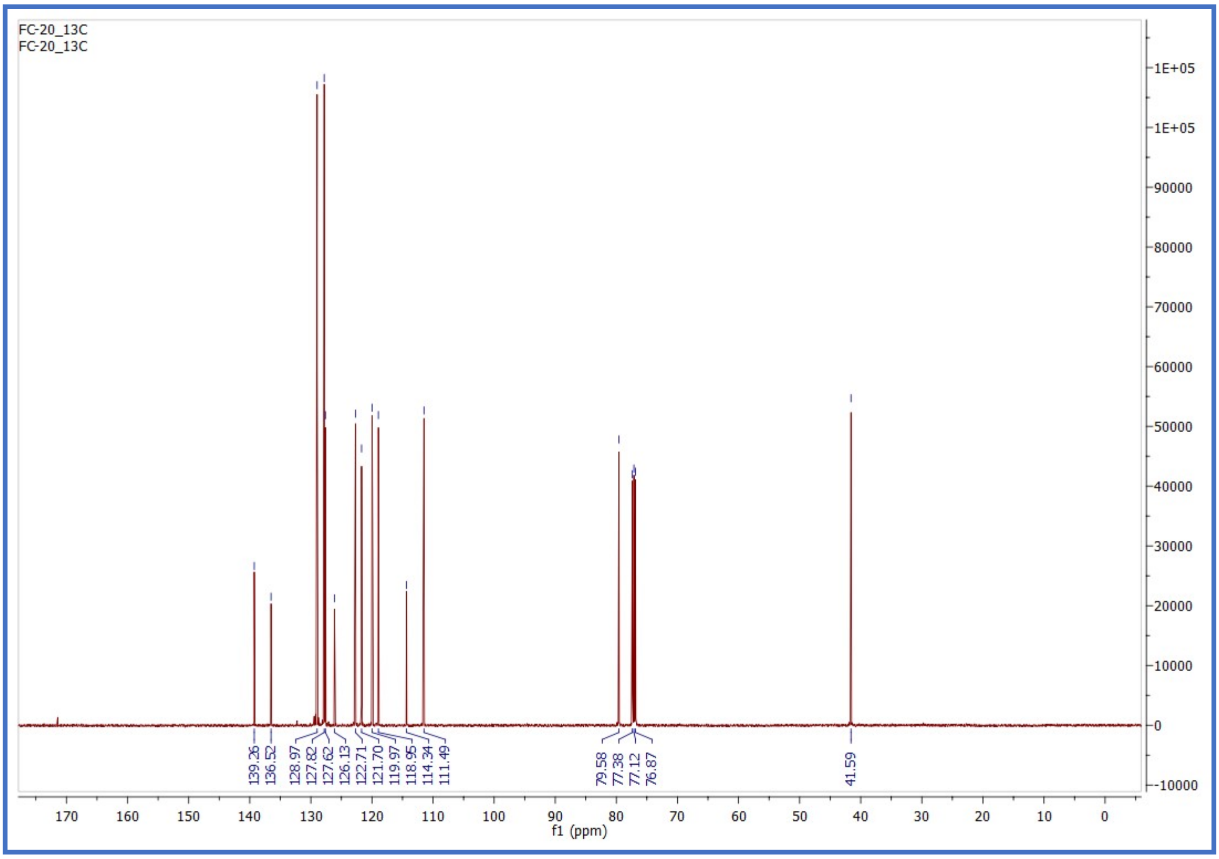

Figure S24: ^{1}H NMR and ^{13}C NMR of 2-methyl-3-(2-nitro-1-phenylethyl)-1H-indole **(4h).**

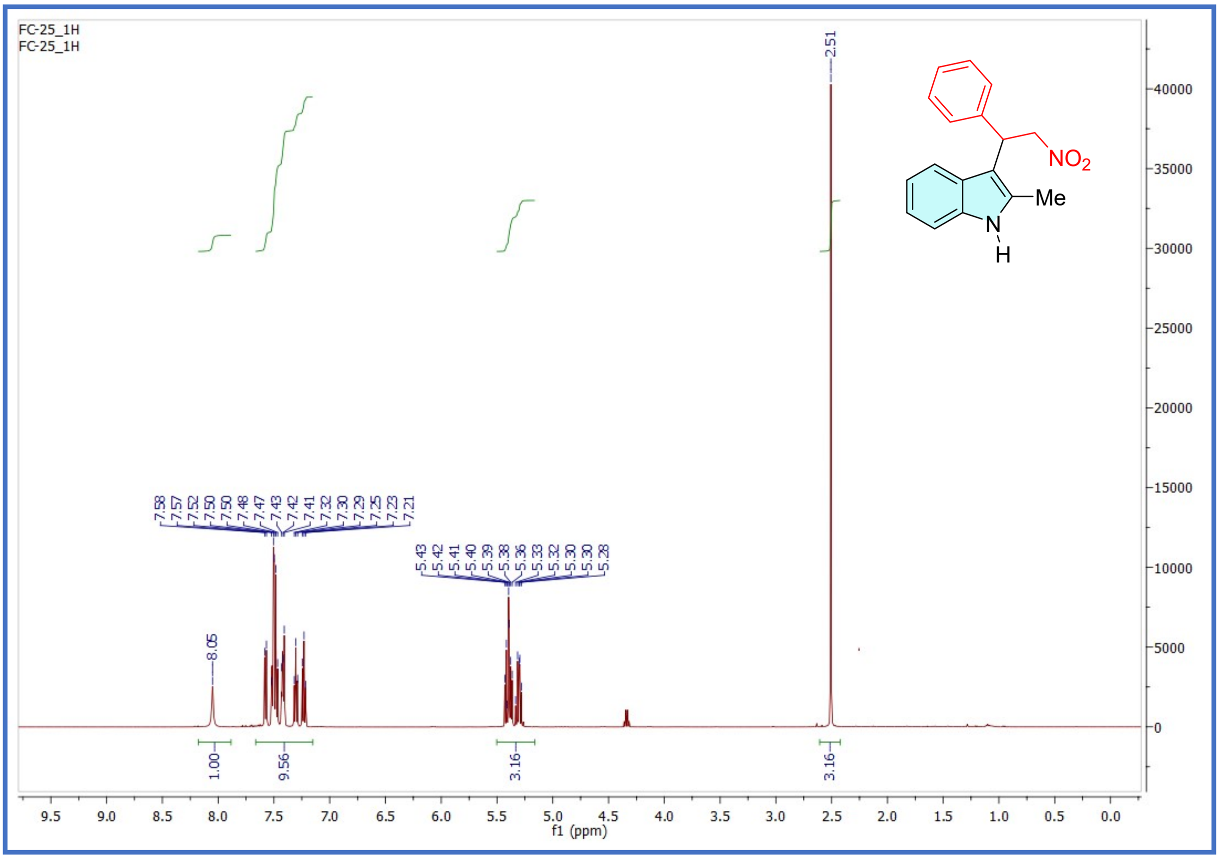

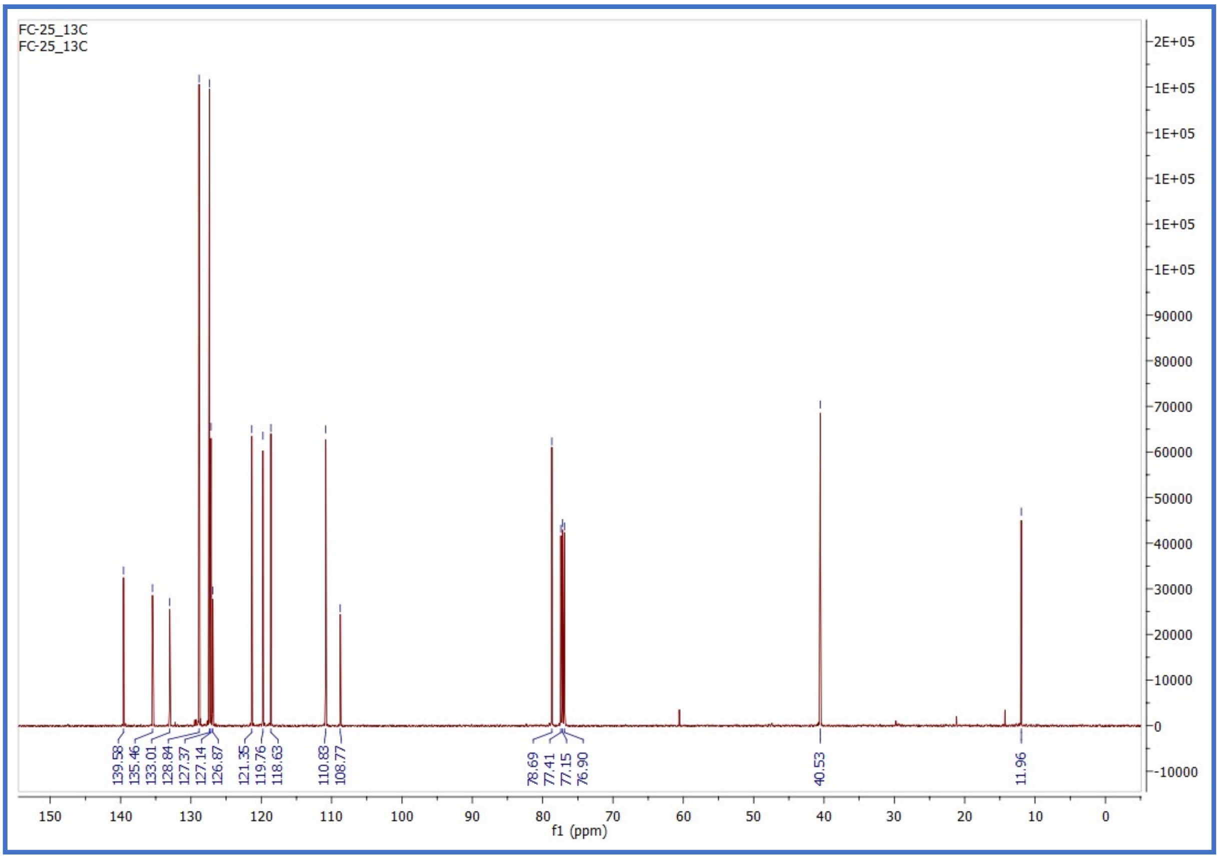

Figure S25: ^{1}H NMR and ^{13}C NMR of 3-(2-Nitro-1-4-bromophenyl-ethyl)-1H-methylindole **(4i).**

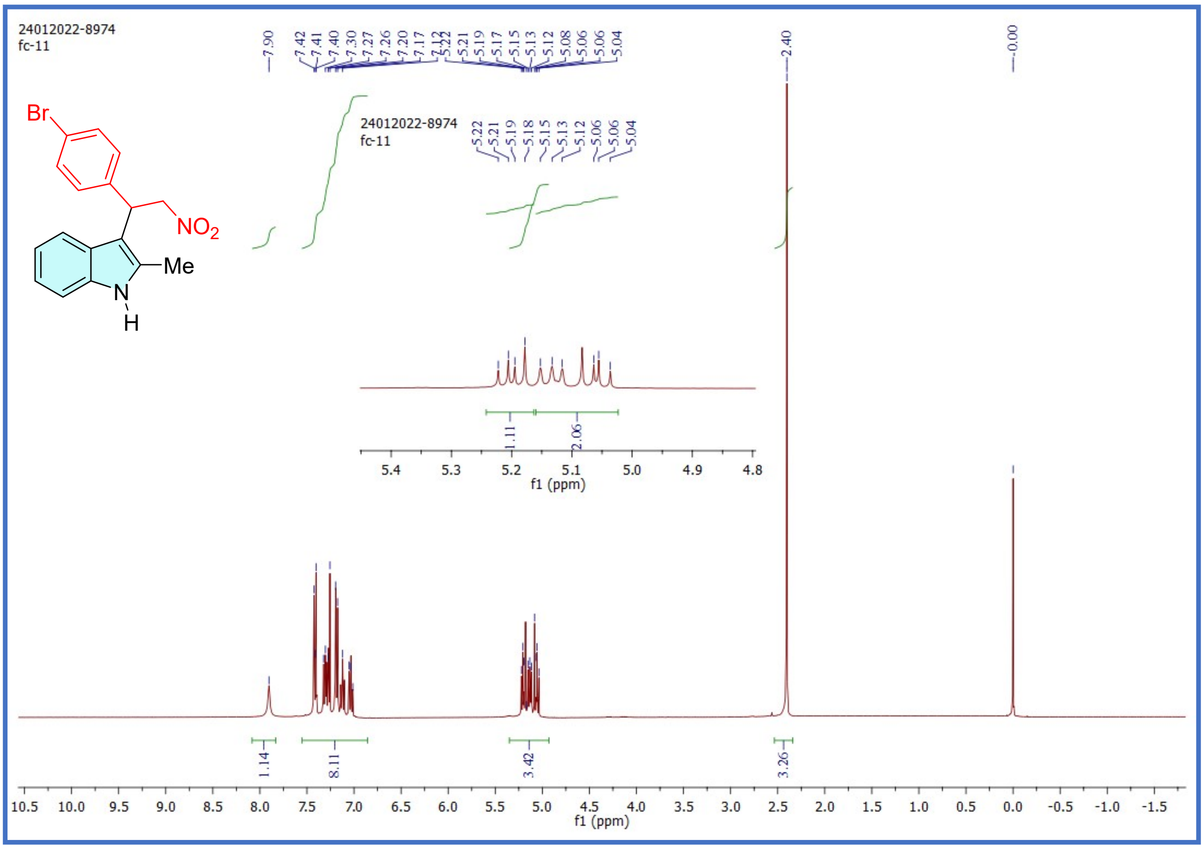

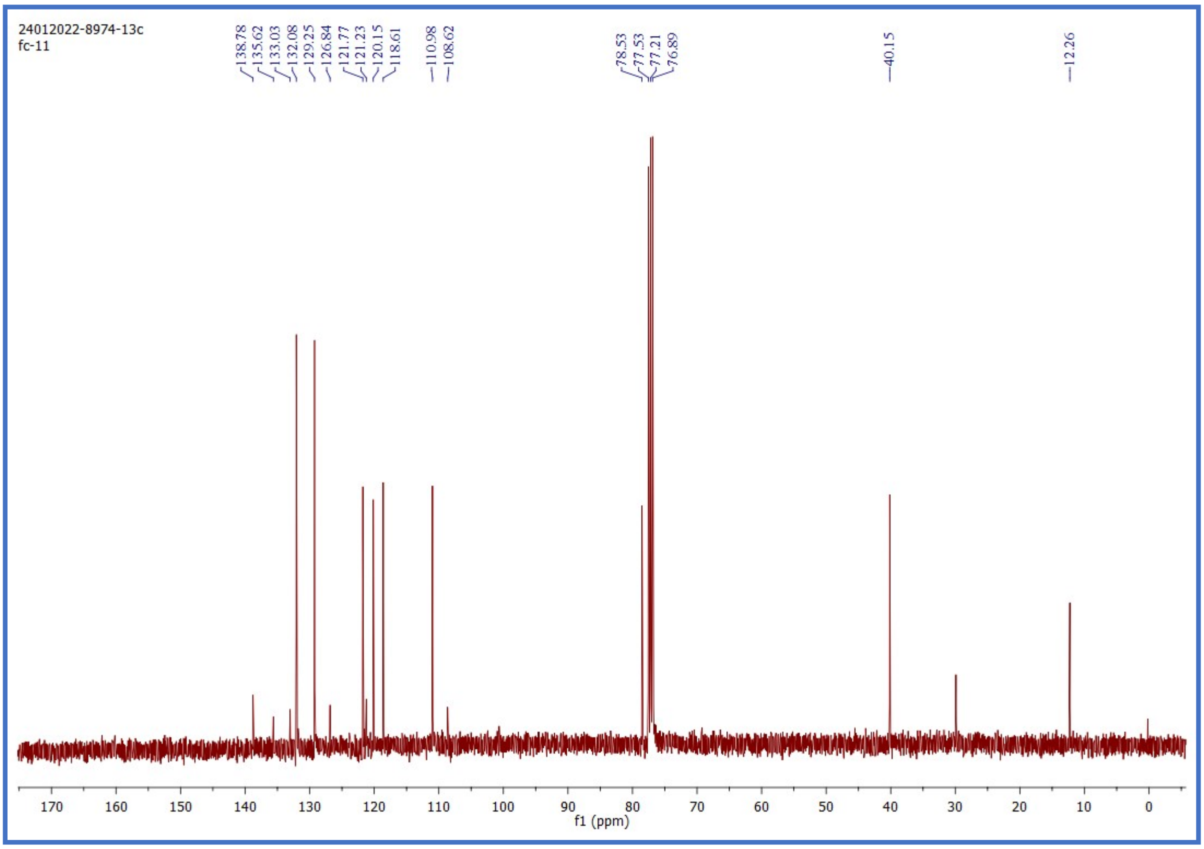

Figure S26: ^{1}H NMR and ^{13}C NMR of 3-(2-Nitro-1-2-chlorophenyl-ethyl)-1H-methylindole **(4j).**

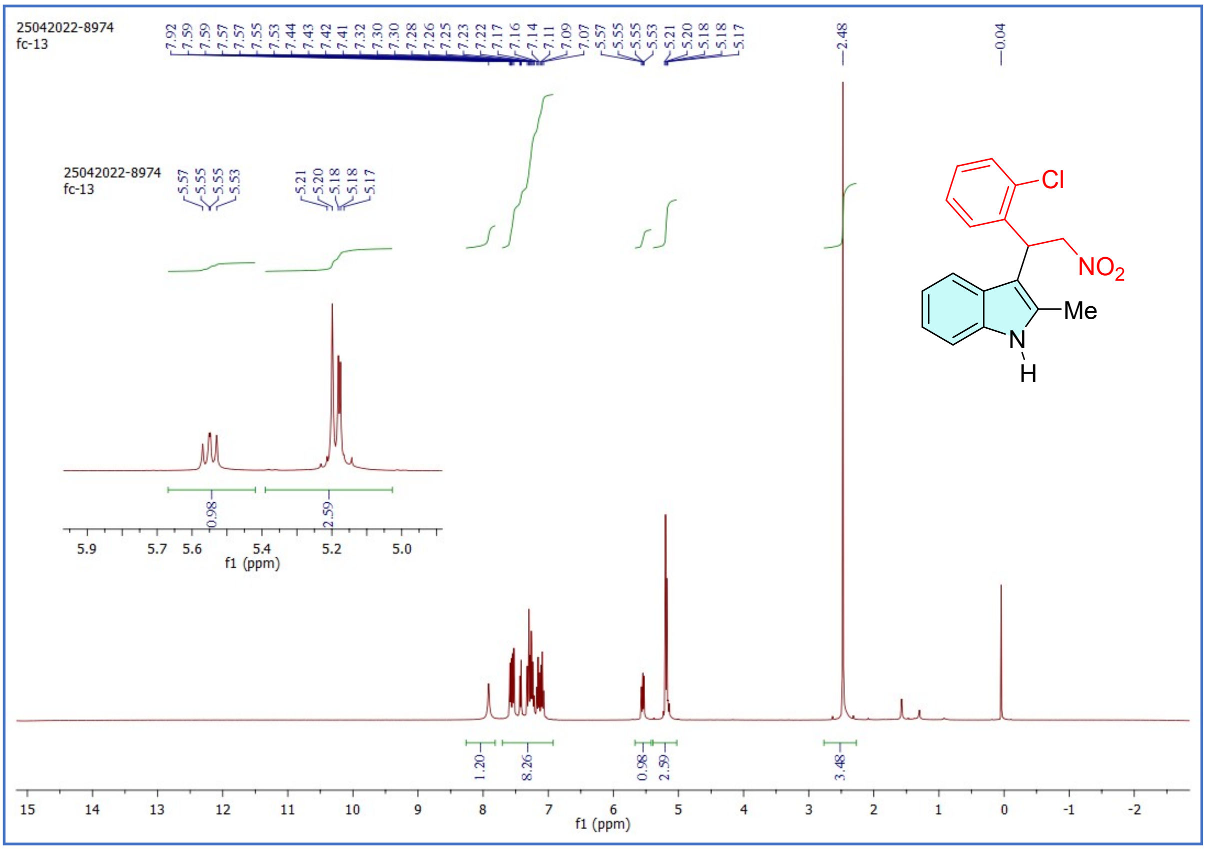

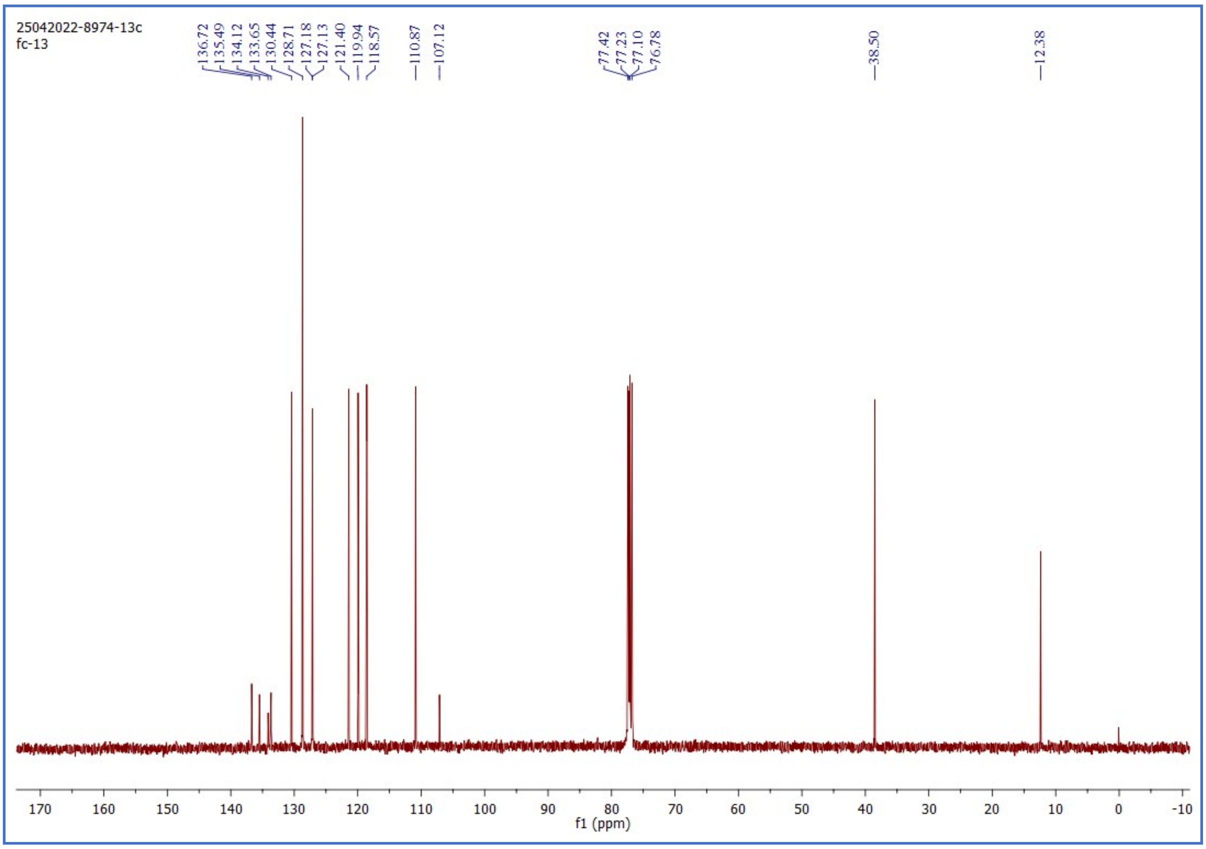

Figure S27: ^{1}H NMR and ^{13}C NMR 1-methyl-3-(2-nitro-1-phenylethyl)-1H-indole **(4k).**

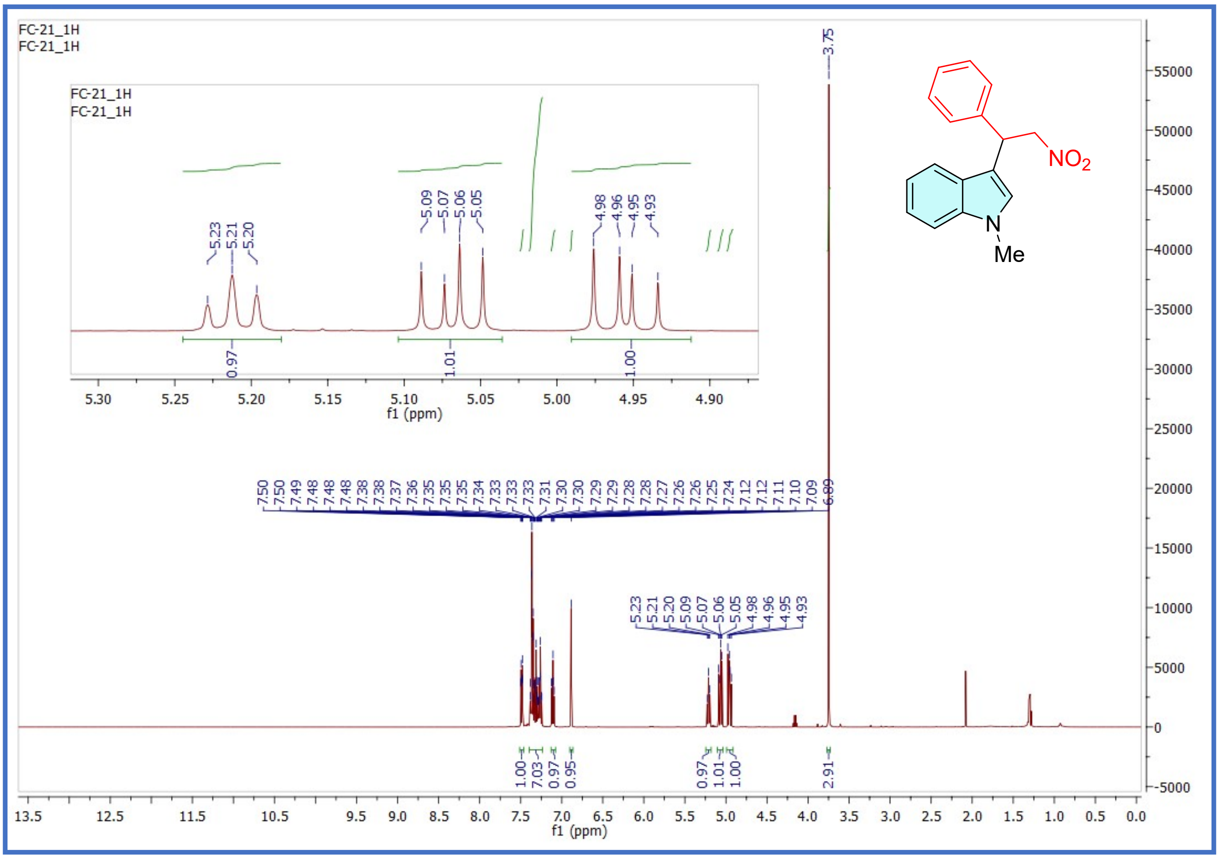

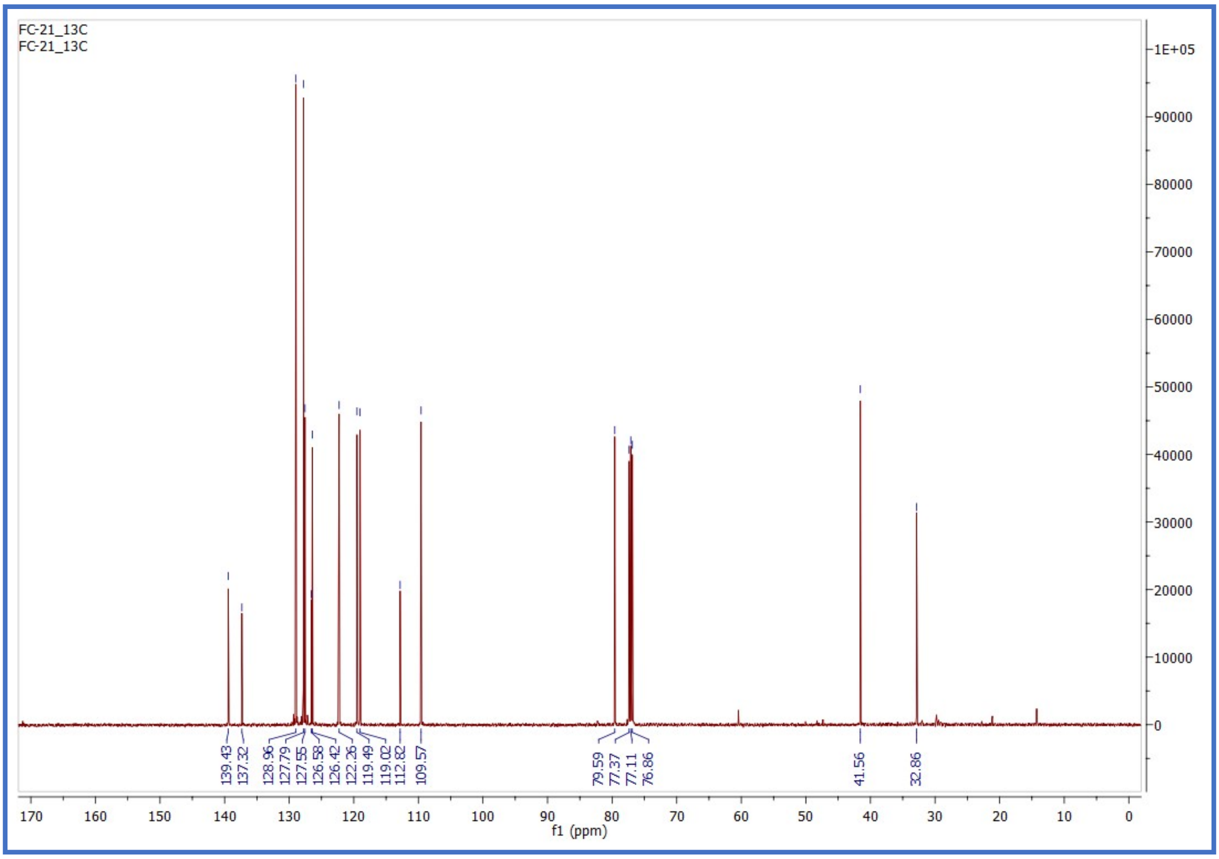

Figure S28: ^{1}H NMR and ^{13}C NMR 3-(1-(4-bromophenyl)-2-nitroethyl)-1-methyl-1H-indole **(4l).**

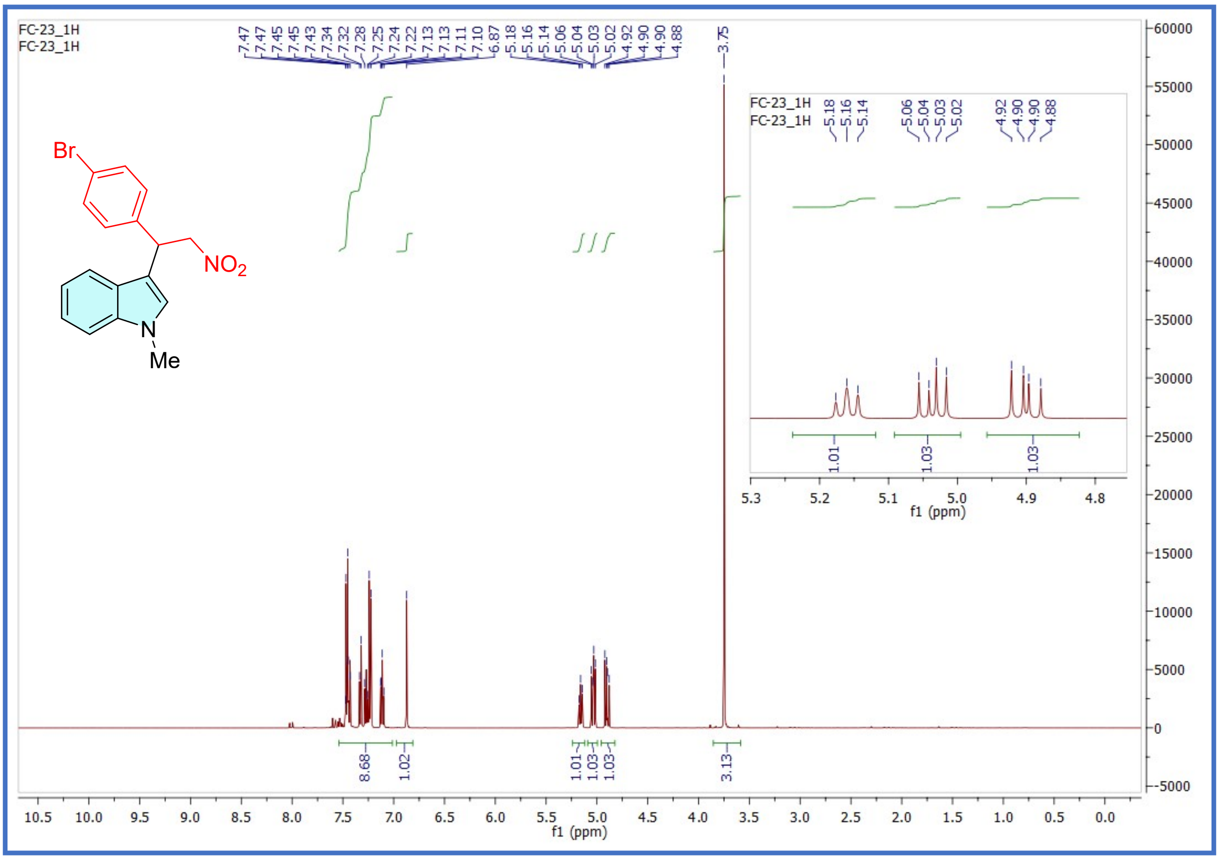

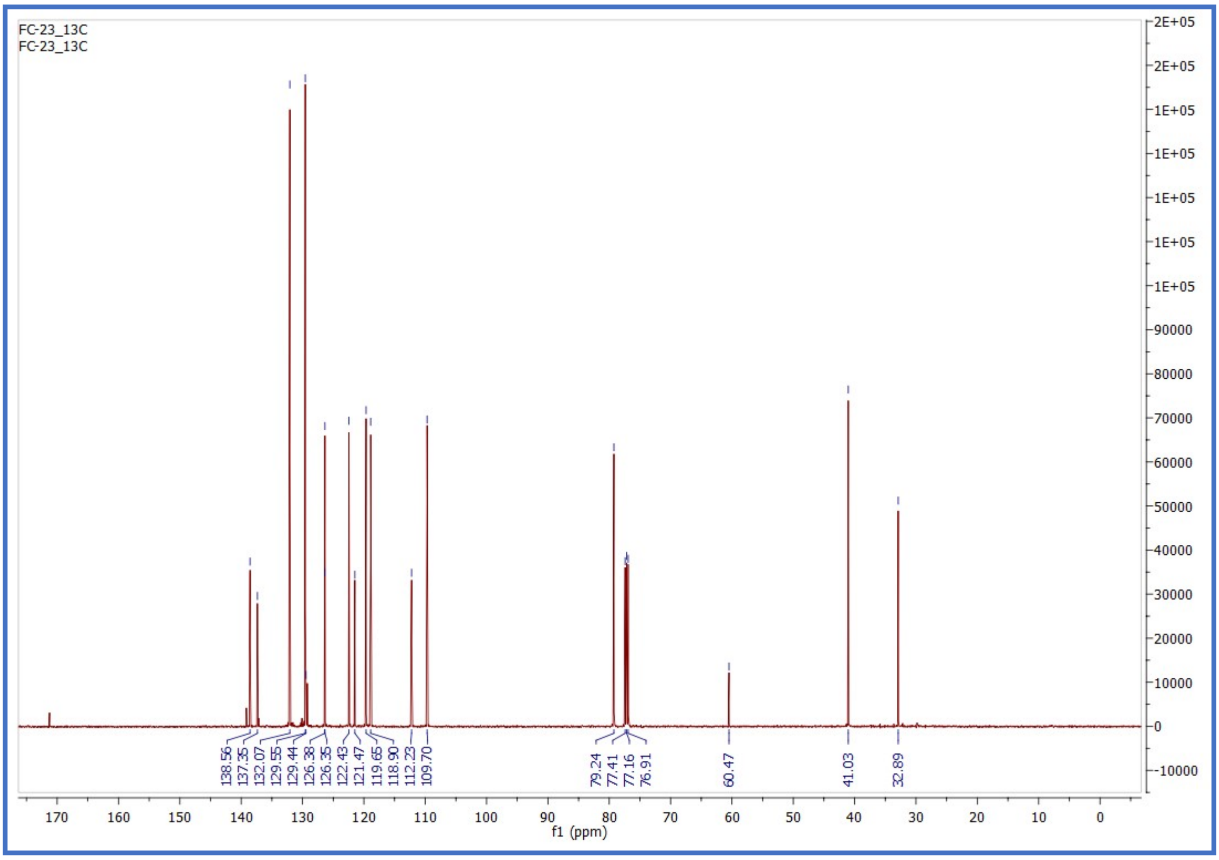

Figure S29: ^{1}H NMR and ^{13}C NMR 1-methyl-3-(2-nitro-1-(p-tolyl)ethyl)-1H-indole **(4m).**

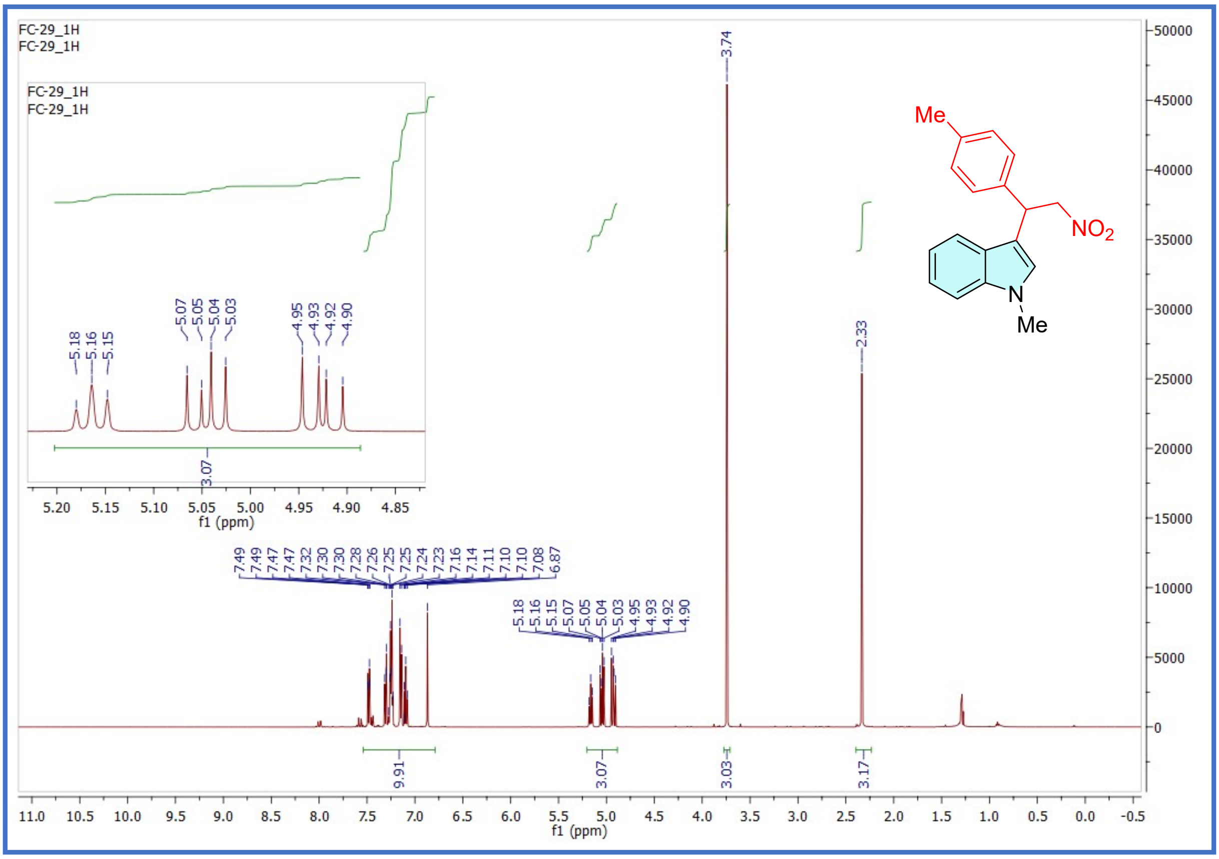

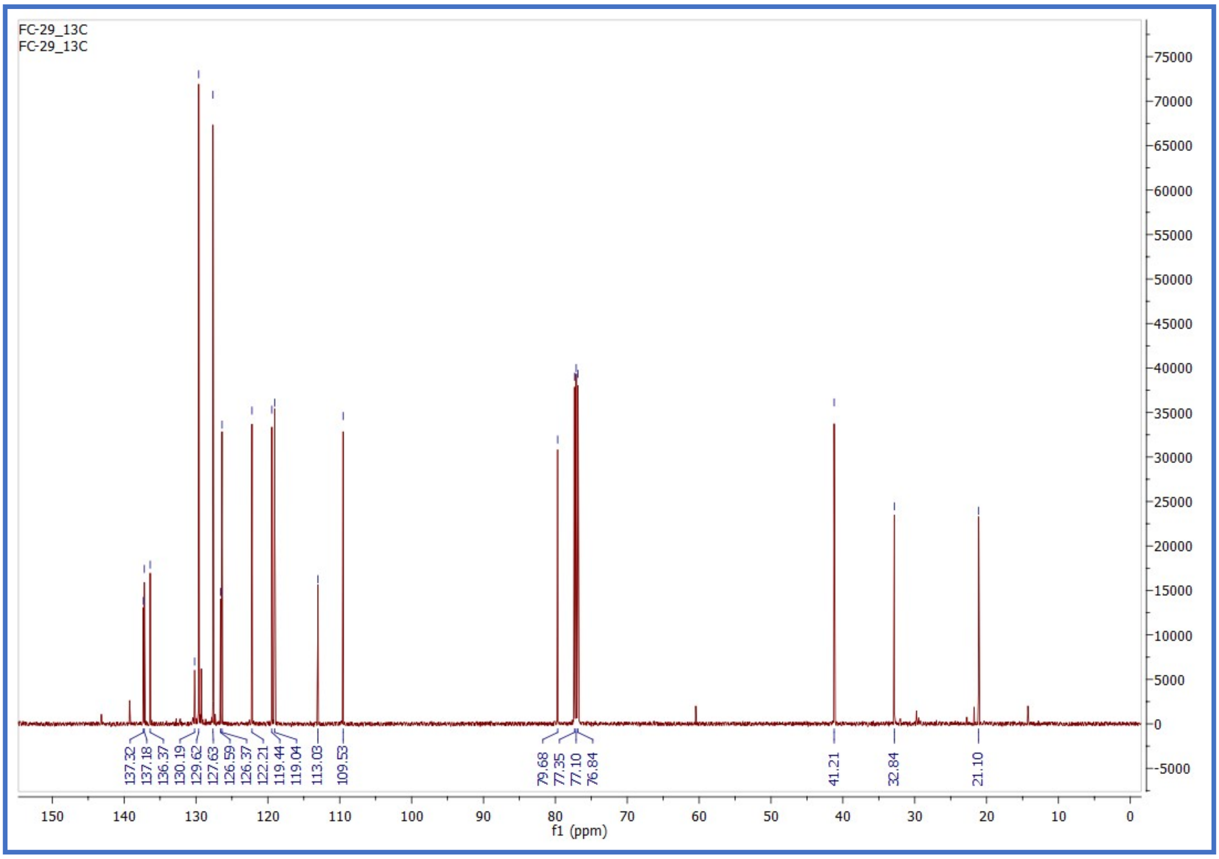

Figure S30: ^{1}H NMR and ^{13}C NMR 1-benzyl-3-(2-nitro-1-phenylethyl)-1H-indole **(4n).**

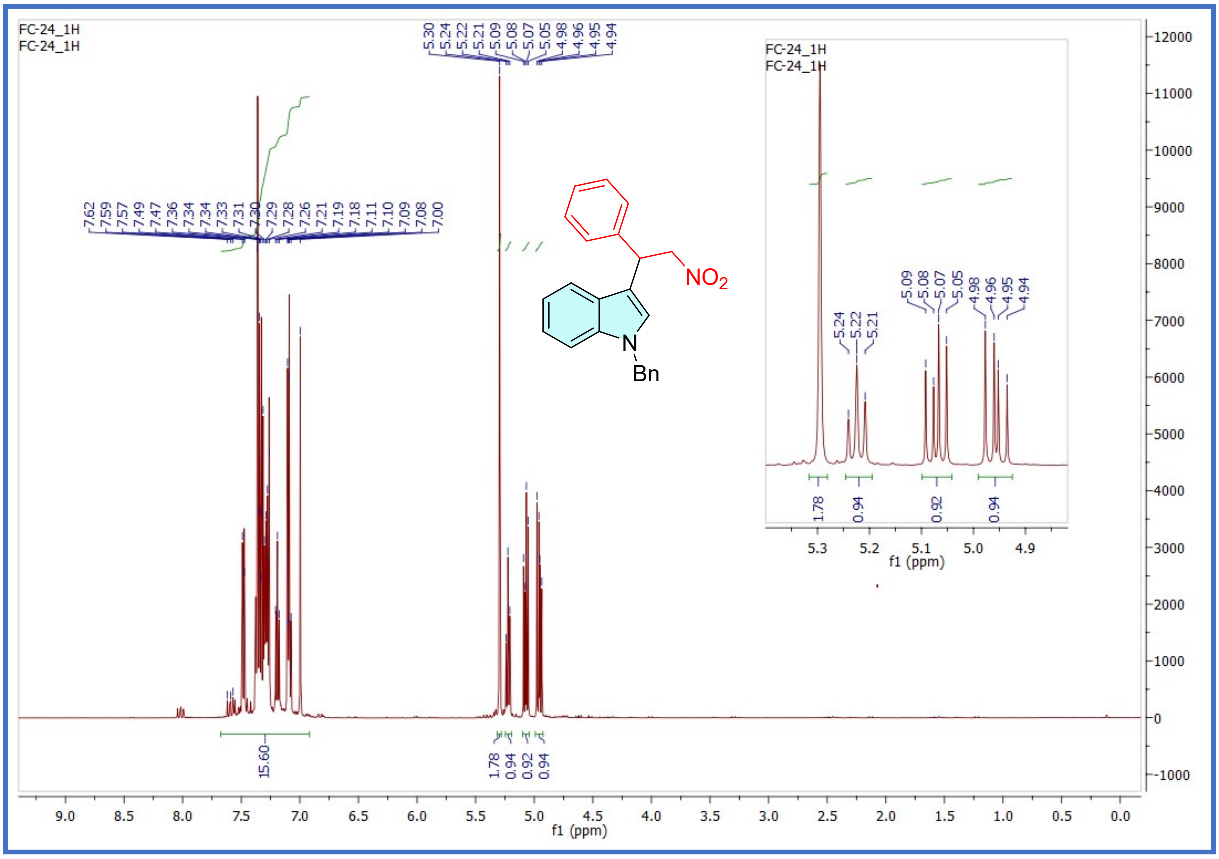

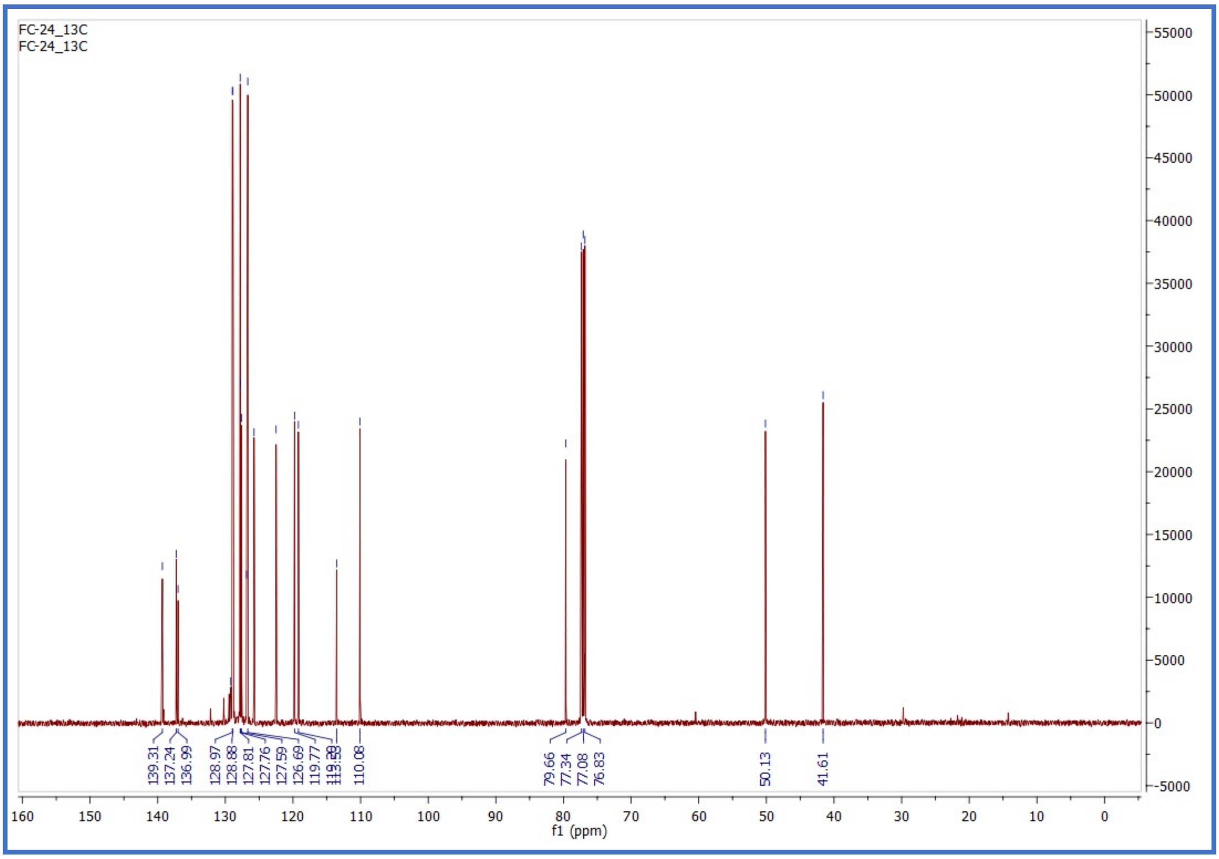

Figure S31: ^{1}H NMR and ^{13}C NMR 5-methyl-3-(2-nitro-1-phenylethyl)-1H-indole **(4o).**

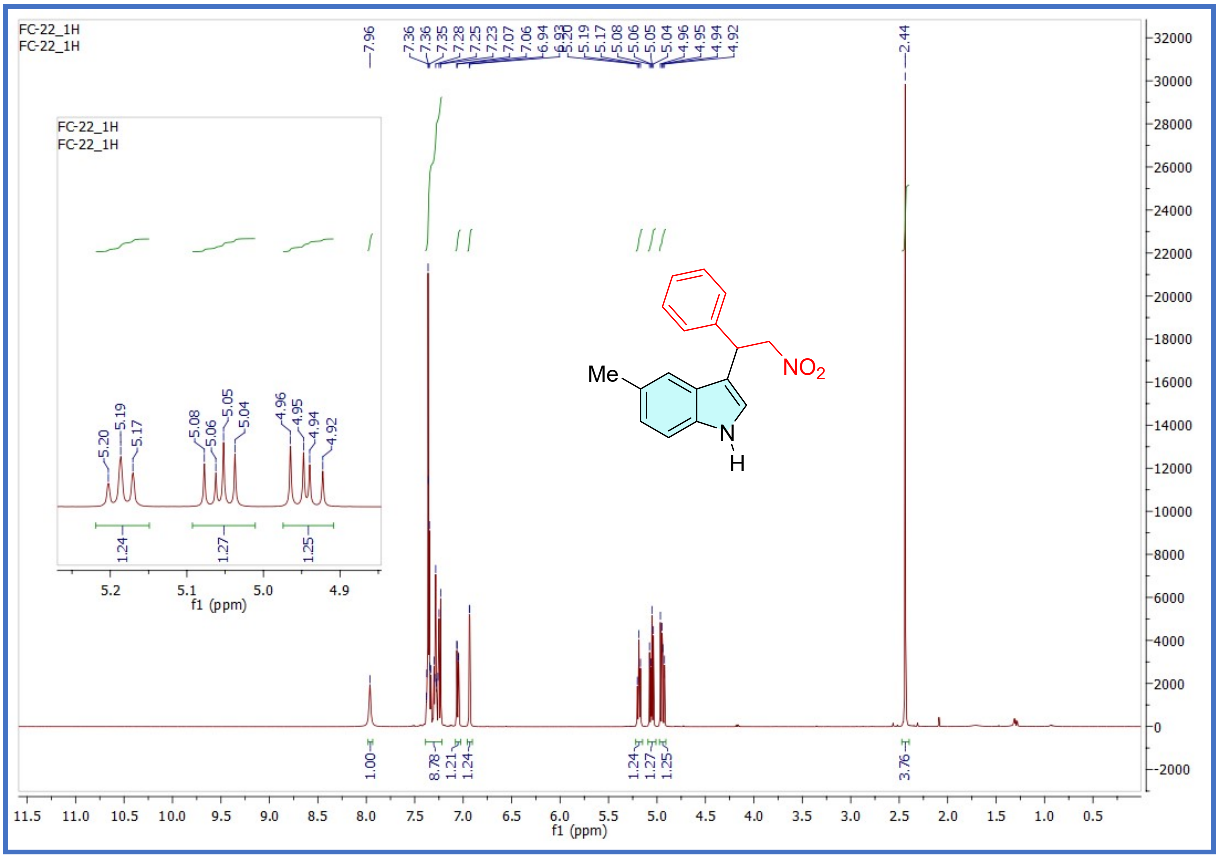

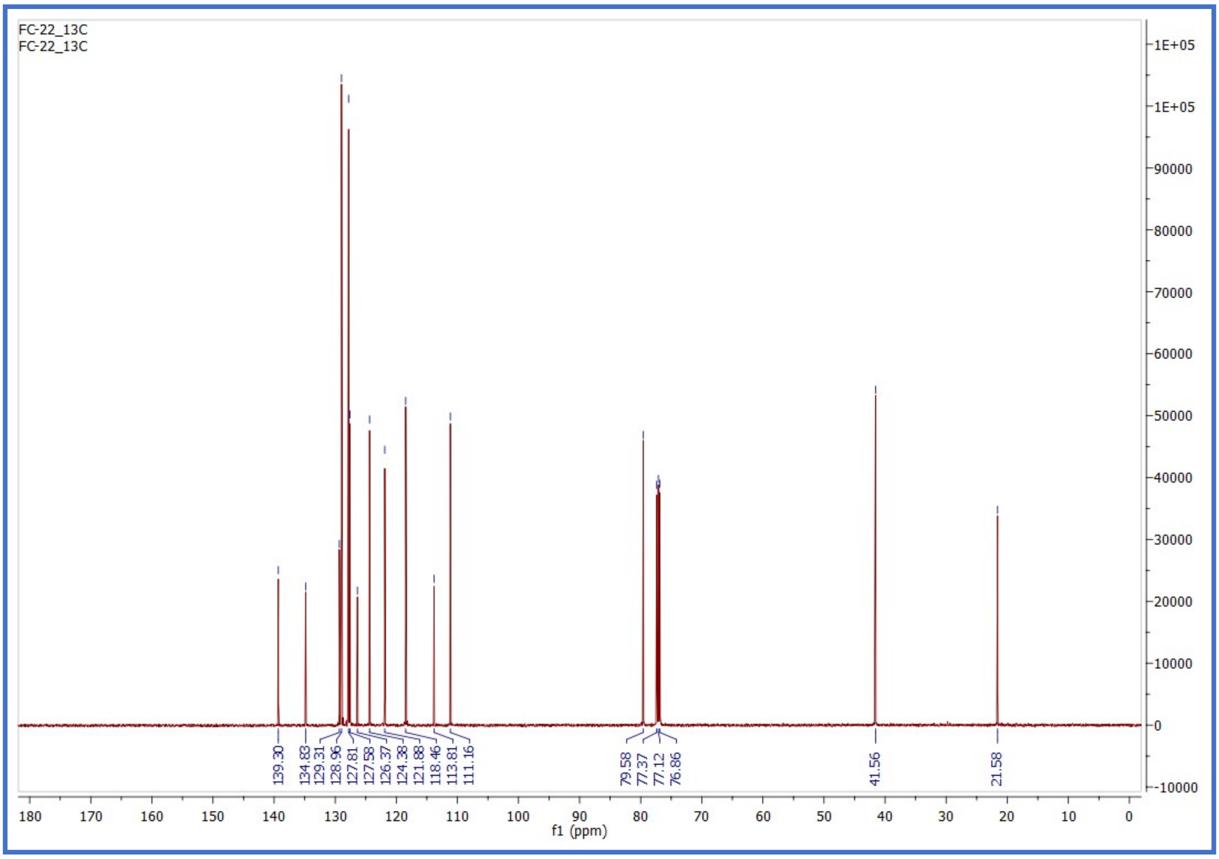

Figure S32: ^{1}H NMR and ^{13}C NMR 3-(2-nitro-1-phenylethyl)-1H-pyrrole **(5a).**

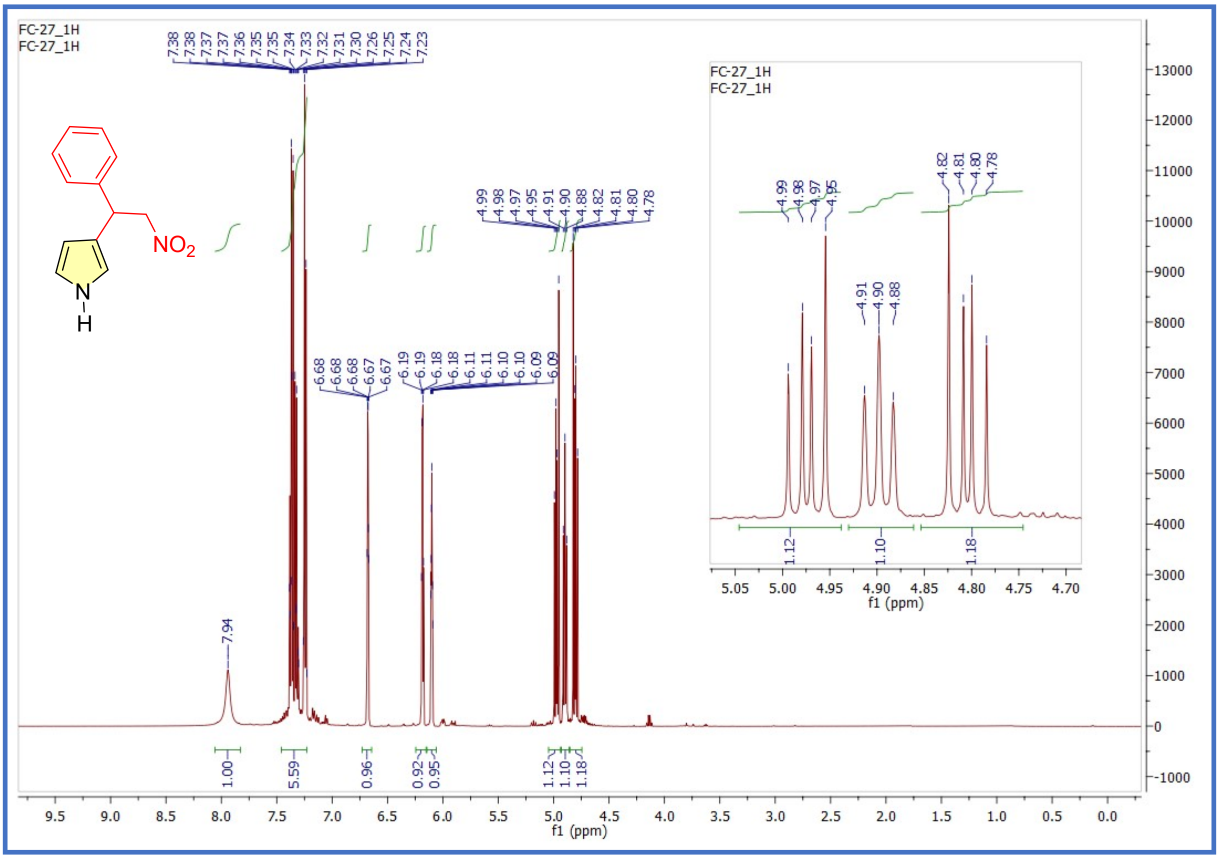

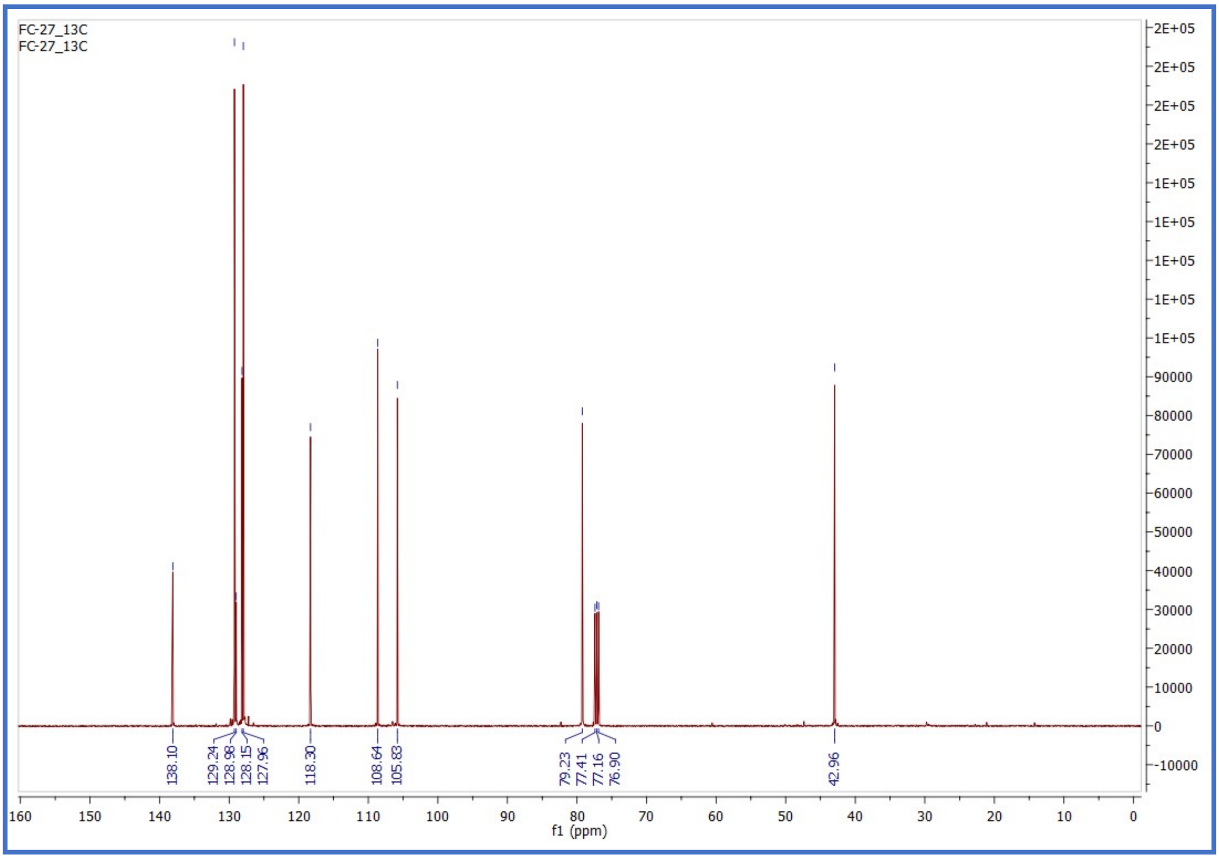

Figure S33: ^1H NMR and ^{13}C NMR 3-(1-(2-chlorophenyl)-2-nitroethyl)-1H-pyrrole **(5b).**

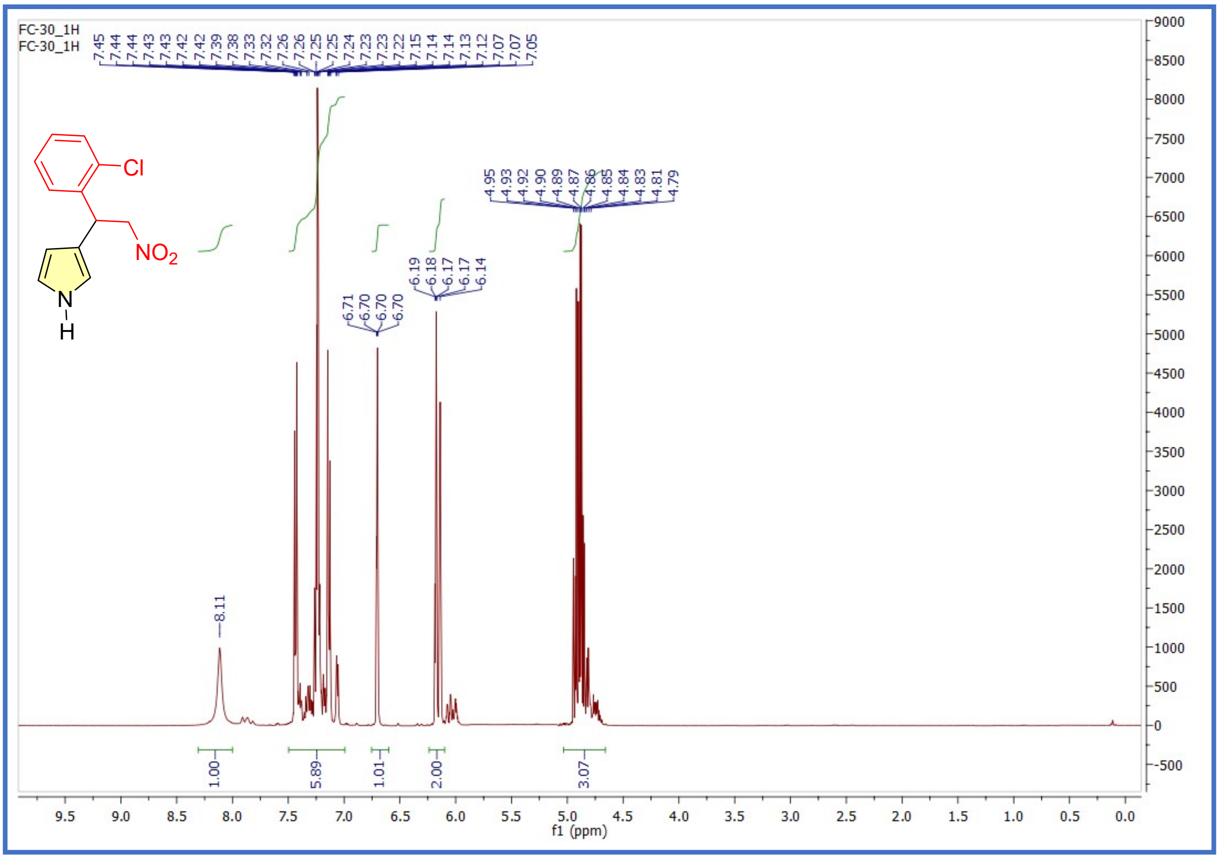

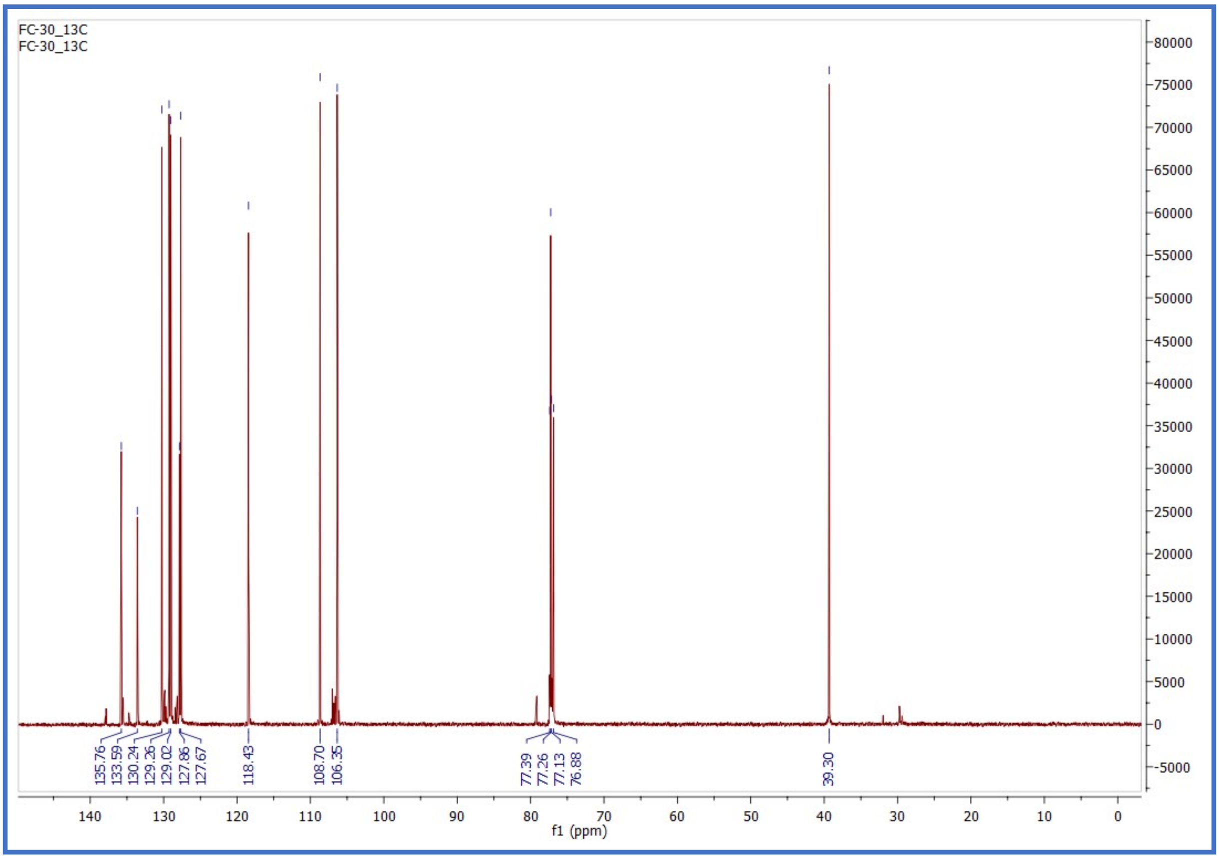

Figure S34: ^{1}H NMR and ^{13}C NMR 3-(1-(4-chlorophenyl)-2-nitroethyl)-1H-pyrrole **(5c).**

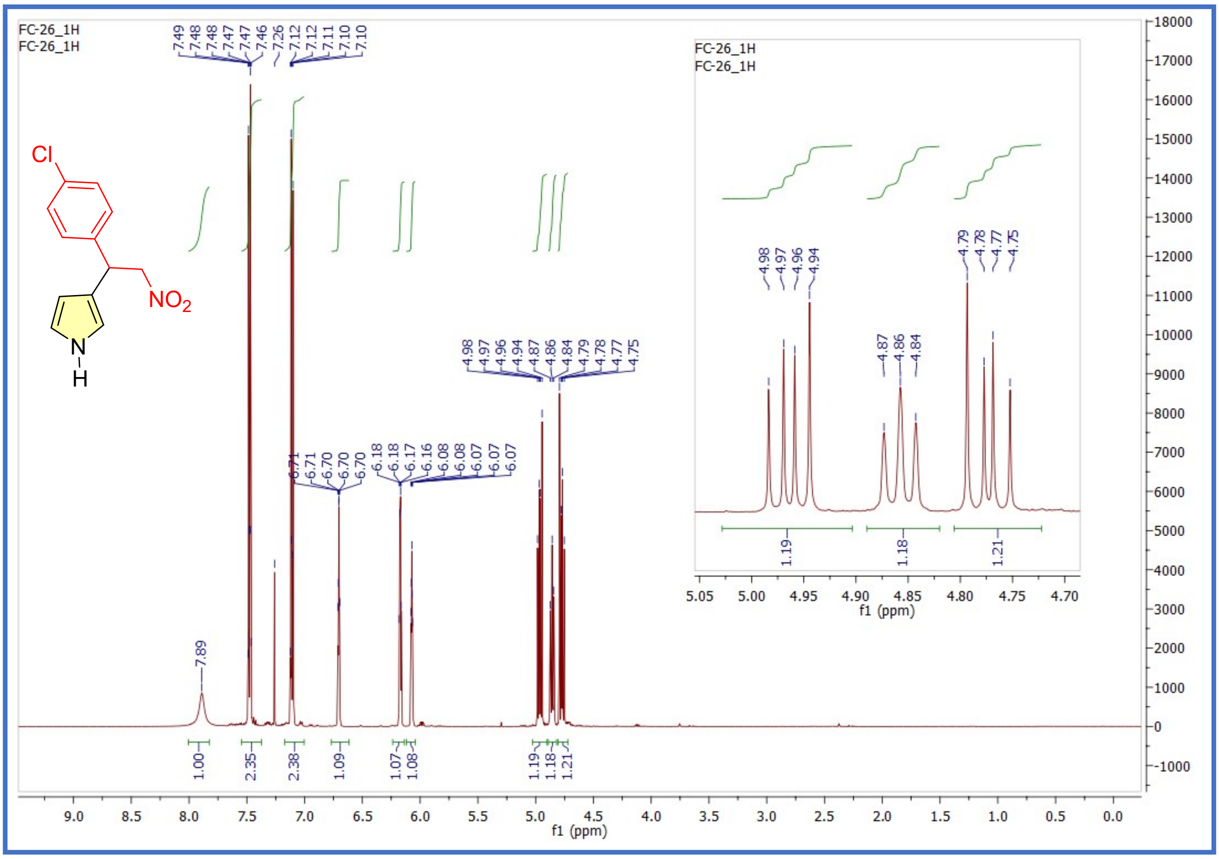

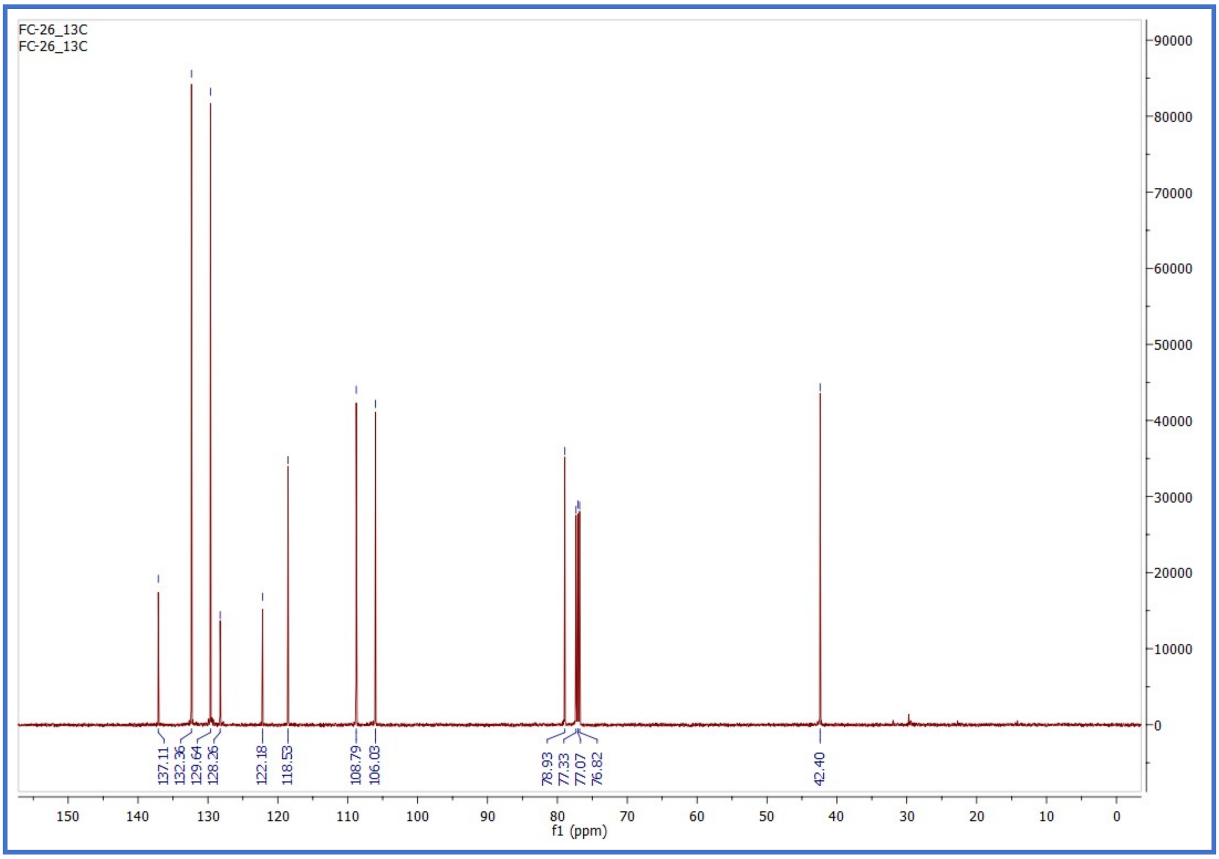

Figure S35: ^{1}H NMR and ^{13}C NMR 3-(2-nitro-1-(p-tolyl)ethyl)-1H-pyrrole **(5d).**

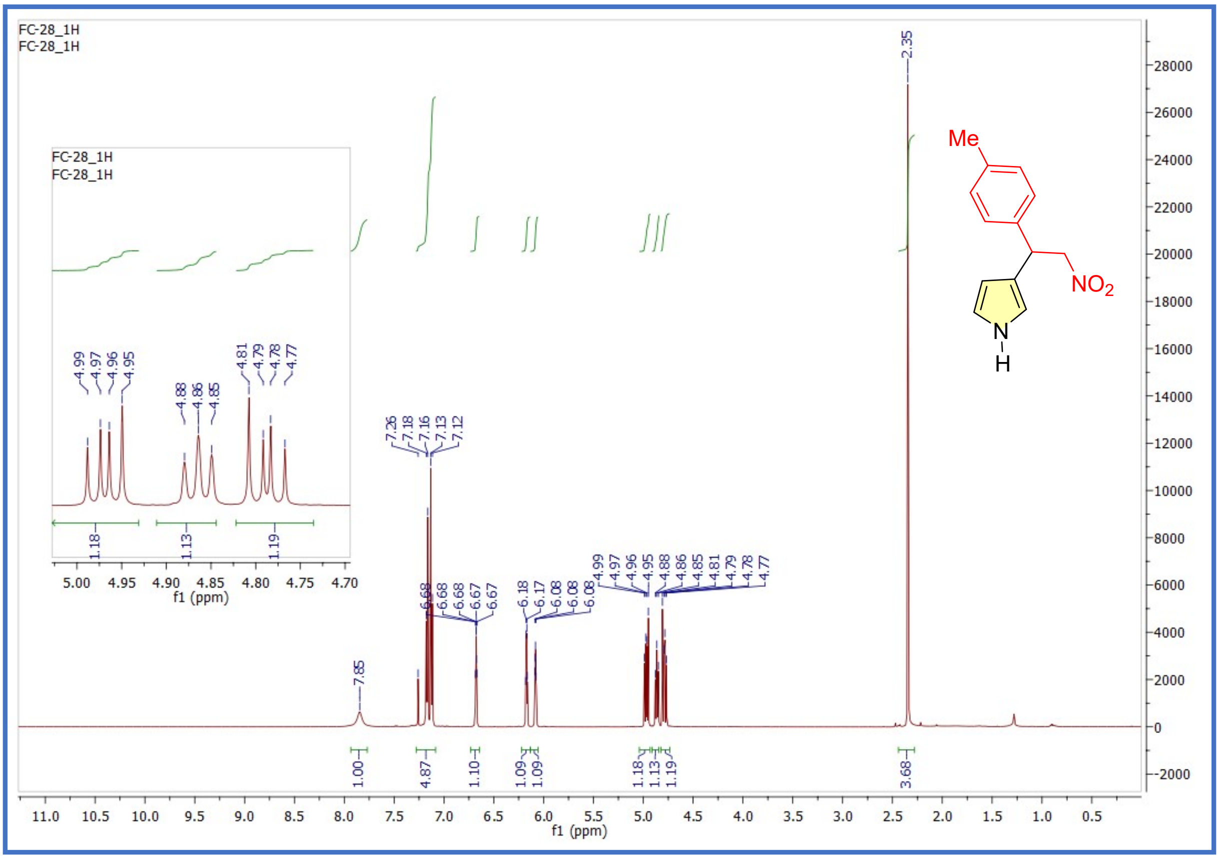

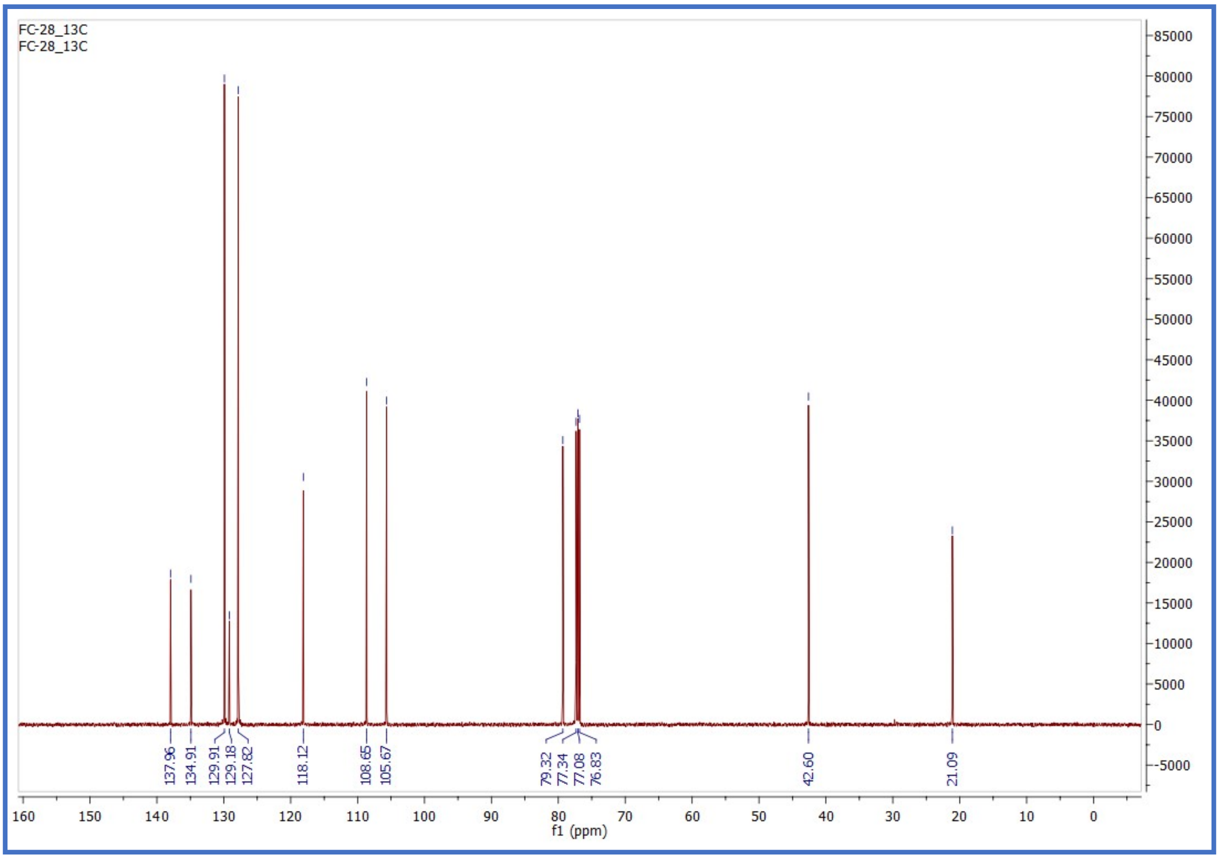

Figure S36: HPLC data of enantioenriched and racemic of **4a**.

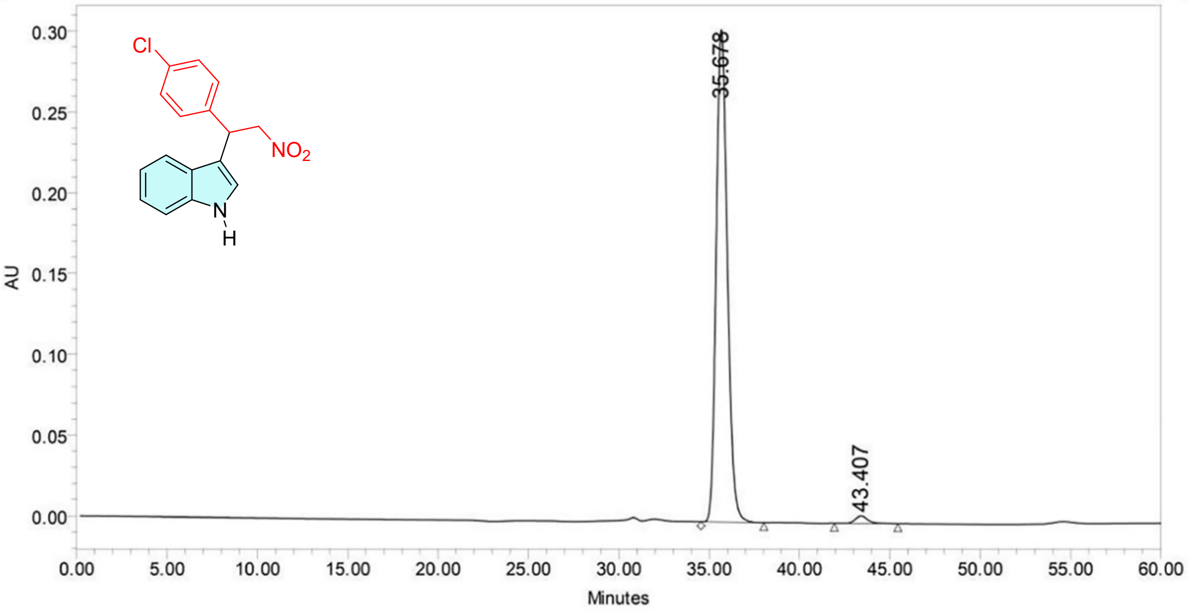

	RT (min)	Area (µV*sec)	% Area	Height (µV)	% Height
1	35.678	12979299	98.31	304588	98.51
2	43.407	223336	1.69	4599	1.49

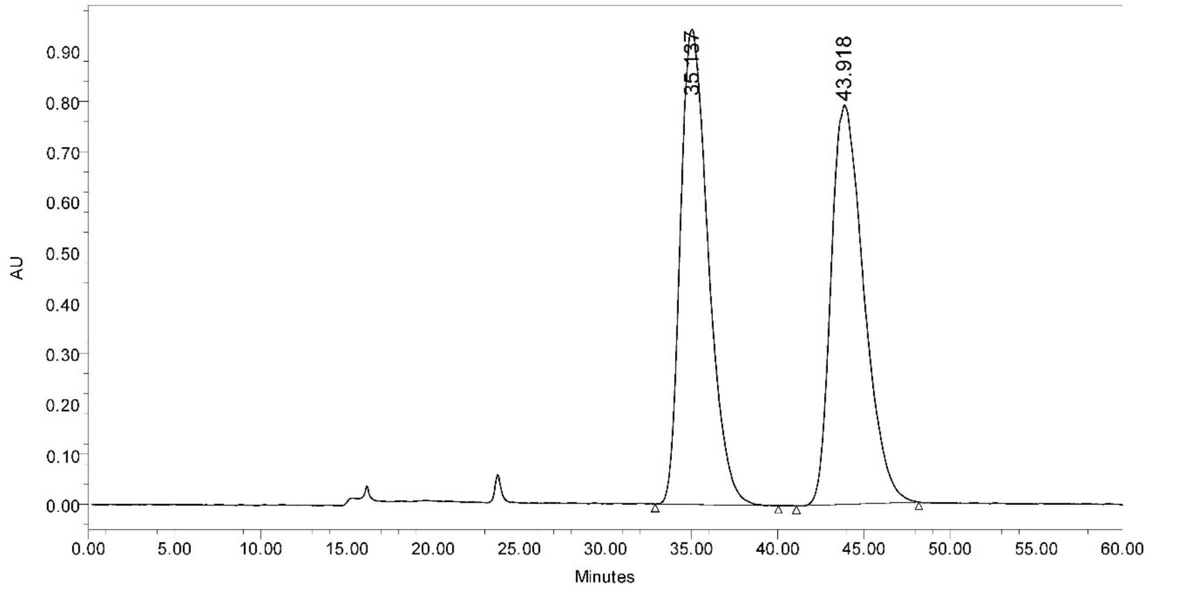

	RT (min)	Area (µV*sec)	% Area	Height (µV)	% Height
1	35.137	10641280	50.28	94325	54.32
2	43.918	10522125	49.72	79314	45.68

Figure S37: HPLC data of enantioenriched and racemic of **4b**.

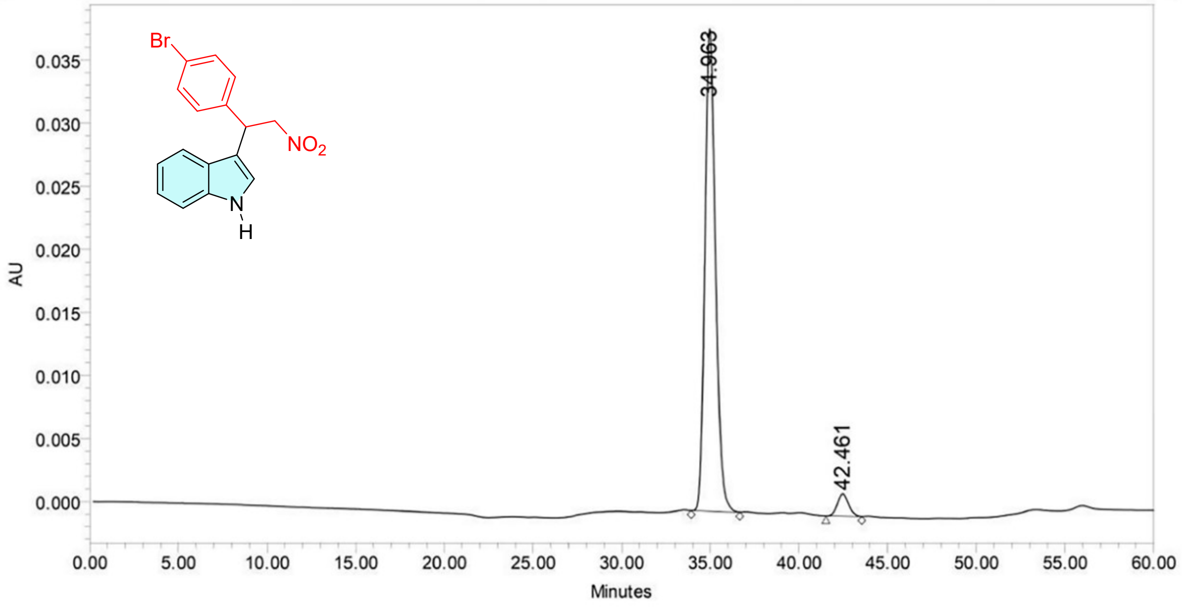

	RT (min)	Area (µV*sec)	% Area	Height (µV)	% Height
1	34.963	1590479	95.35	38242	95.61
2	42.461	77535	4.65	1758	4.39

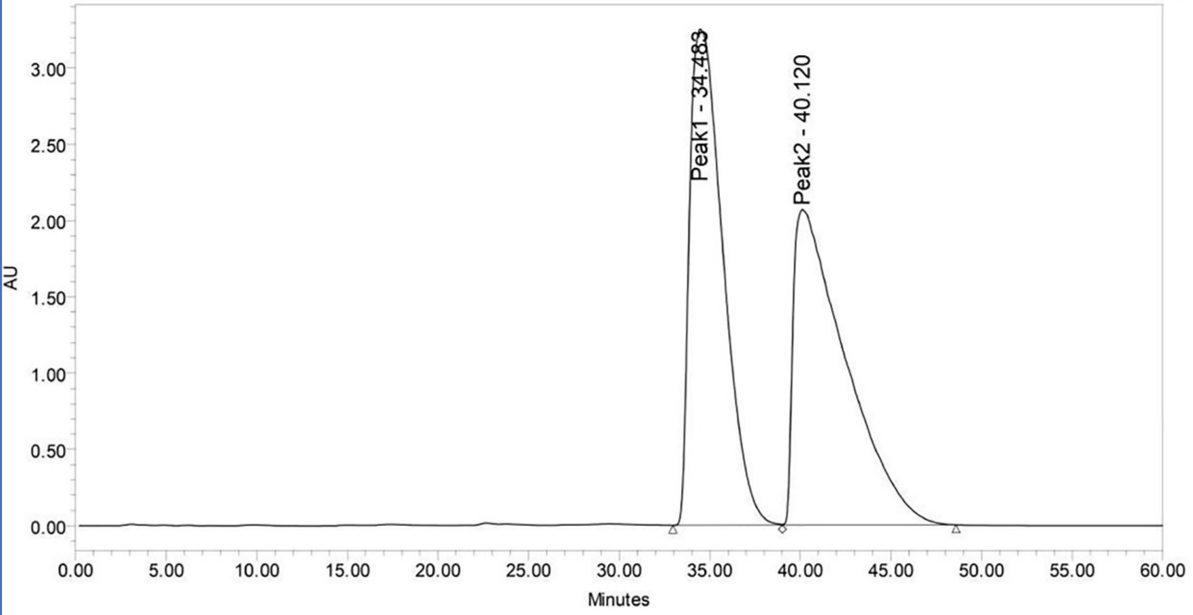

	Peak Name	RT (min)	Area (µV*sec)	% Area	Height (µV)	% Height
1	Peak1	34.483	416885486	50.05	3251024	61.11
2	Peak2	40.120	416045858	49.95	2068850	38.89

Figure S38: HPLC data of enantioenriched and racemic of **4c**.

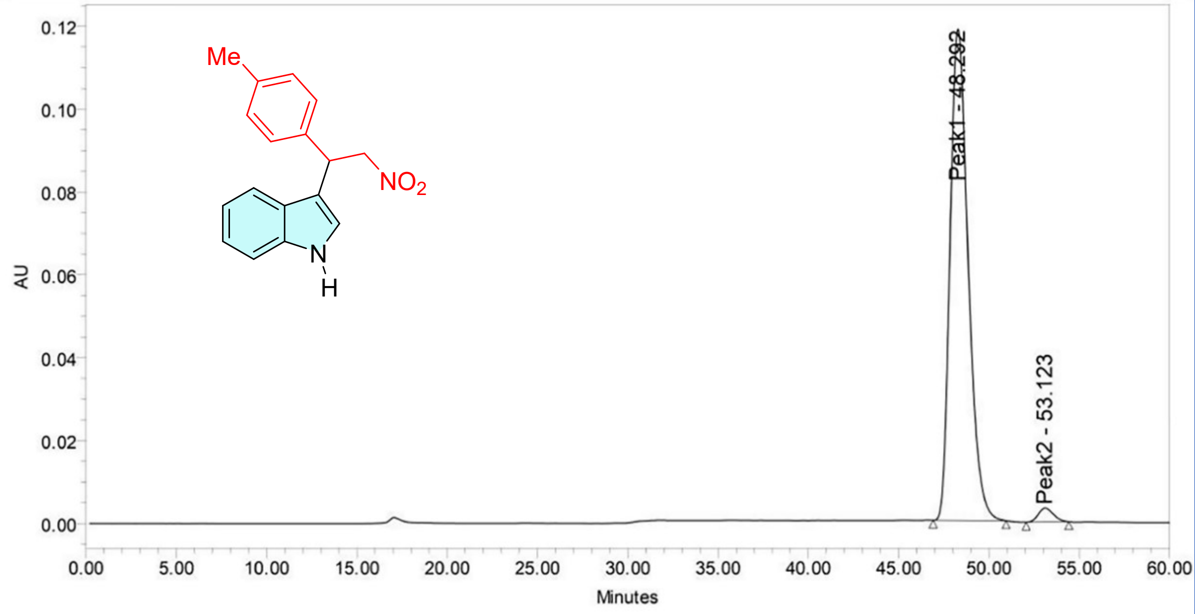

	Peak Name	RT (min)	Area (µV*sec)	% Area	Height (µV)	% Height
1	Peak1	48.292	8217636	97.71	118484	97.26
2	Peak2	53.123	192663	2.29	3333	2.74

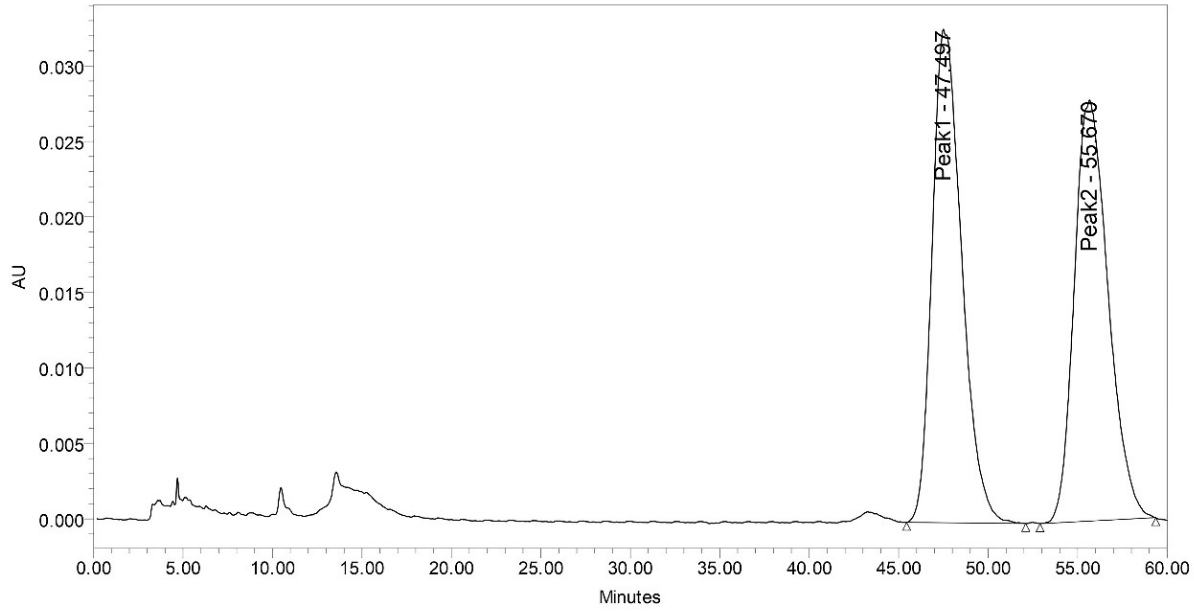

	Peak Name	RT (min)	Area (µV*sec)	% Area	Height (µV)	% Height
1	Peak1	47.497	3761594	50.59	32671	53.96
2	Peak2	55.670	3673762	49.41	27877	46.04

Figure S39: HPLC data of enantioenriched and racemic of **4d**.

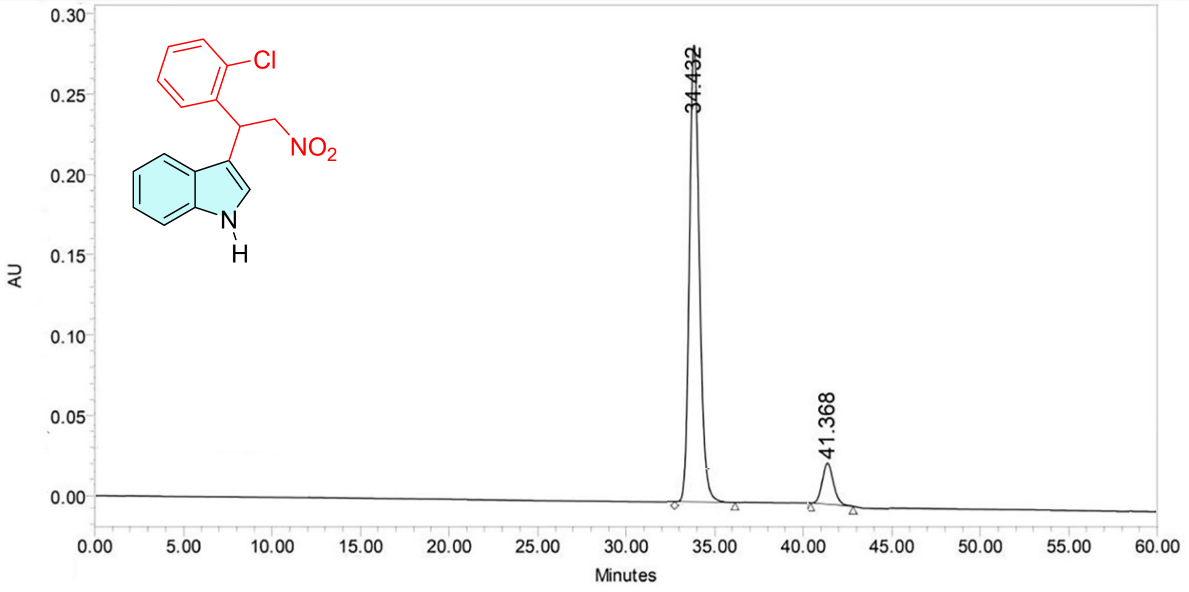

	RT (min)	Area (µV*sec)	% Area	Height (µV)	% Height
1	34.432	748137	90.75	19168	91.71
2	41.368	76239	9.25	1733	8.29

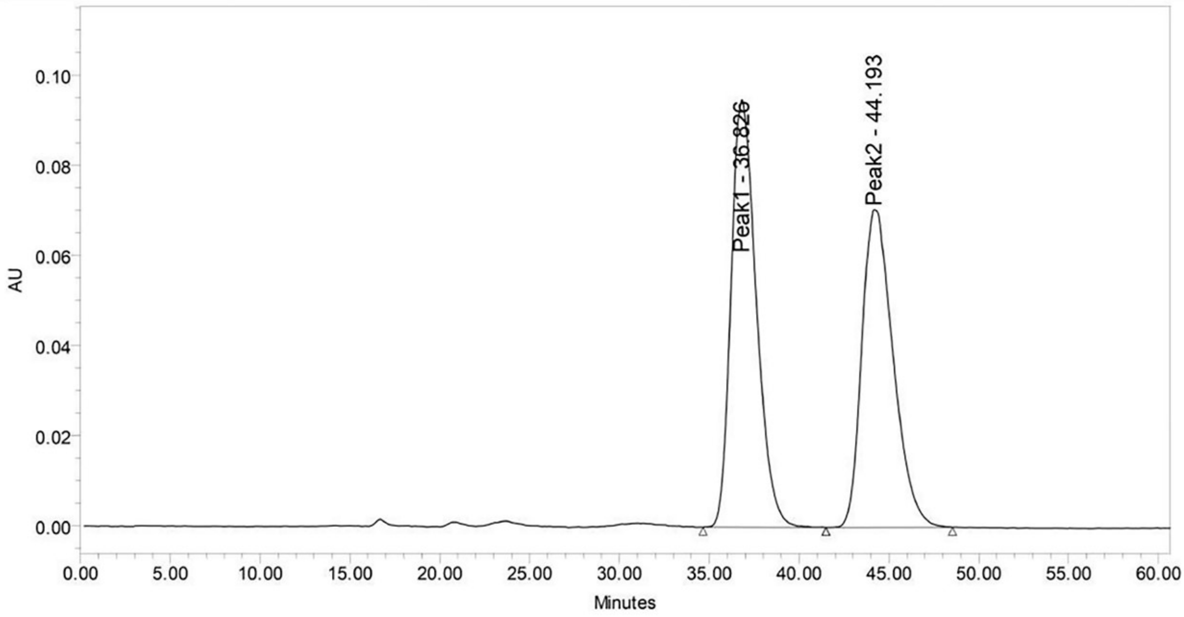

	Peak Name	RT (min)	Area (µV*sec)	% Area	Height (µV)	% Height
1	Peak1	36.826	9271940	52.03	94826	57.37
2	Peak2	44.193	8549610	47.97	70462	42.63

Figure S40: HPLC data of enantioenriched and racemic of **4e**.

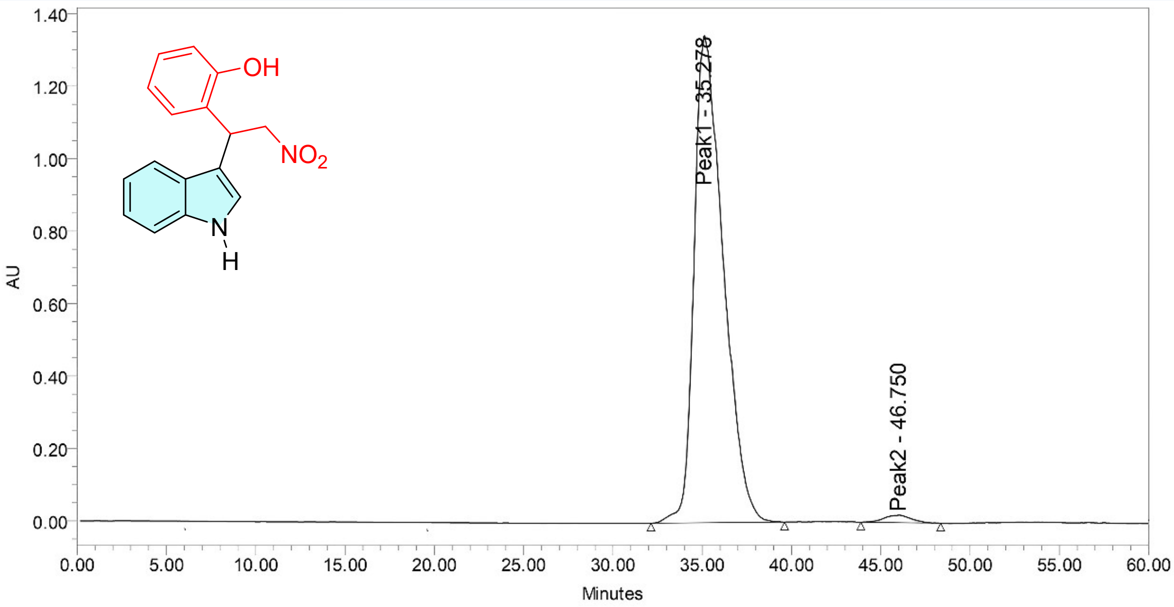

	Peak Name	RT (min)	Area (µV*sec)	% Area	Height (µV)	% Height
1	Peak1	35.278	152615283	98.54	1345113	98.46
2	Peak2	46.750	2263248	1.46	21044	1.54

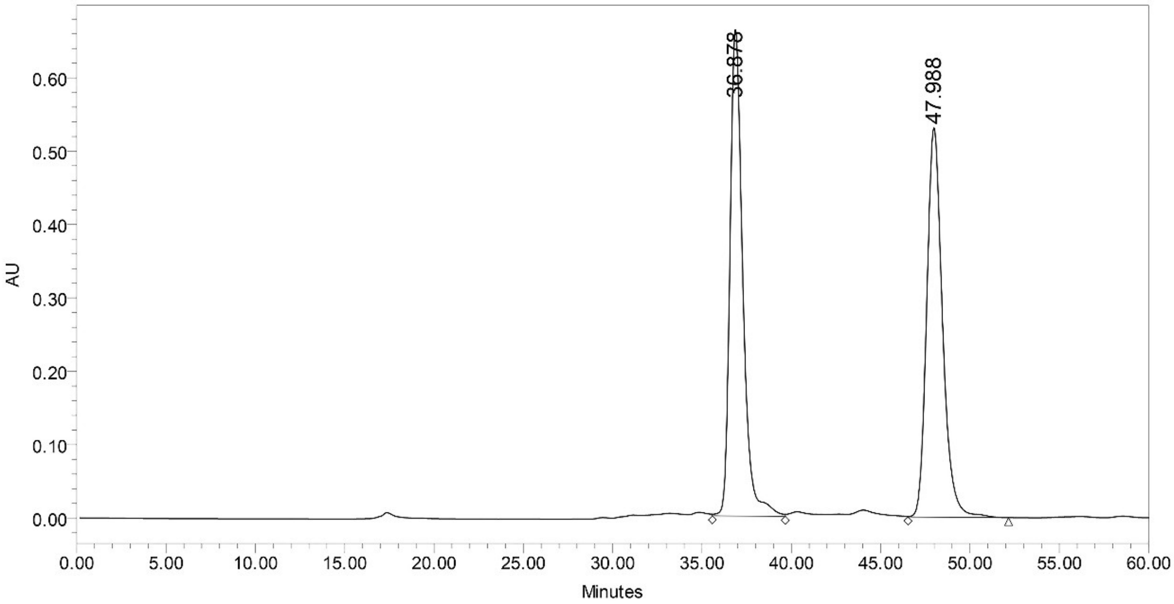

	RT (min)	Area (µV*sec)	% Area	Height (µV)	% Height
1	36.878	33157670	50.40	662754	55.55
2	47.988	32634095	49.60	530362	44.45

Figure S41: HPLC data of enantioenriched and racemic of **4f**.

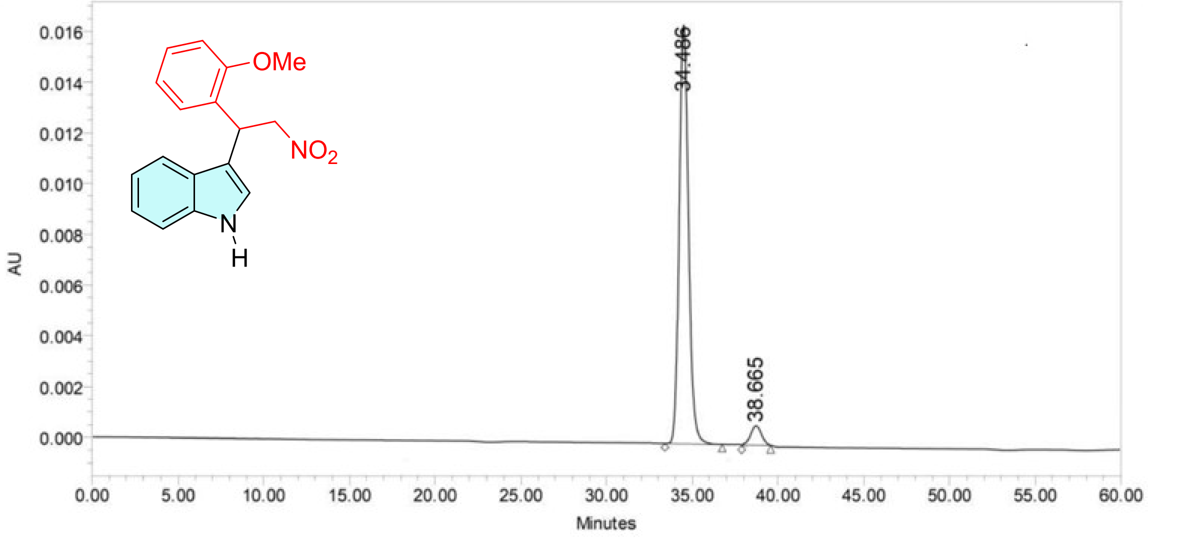

	RT (min)	Area (µV*sec)	% Area	Height (µV)	% Height
1	34.486	706616	95.46	17551	95.84
2	38.665	33629	4.54	762	4.16

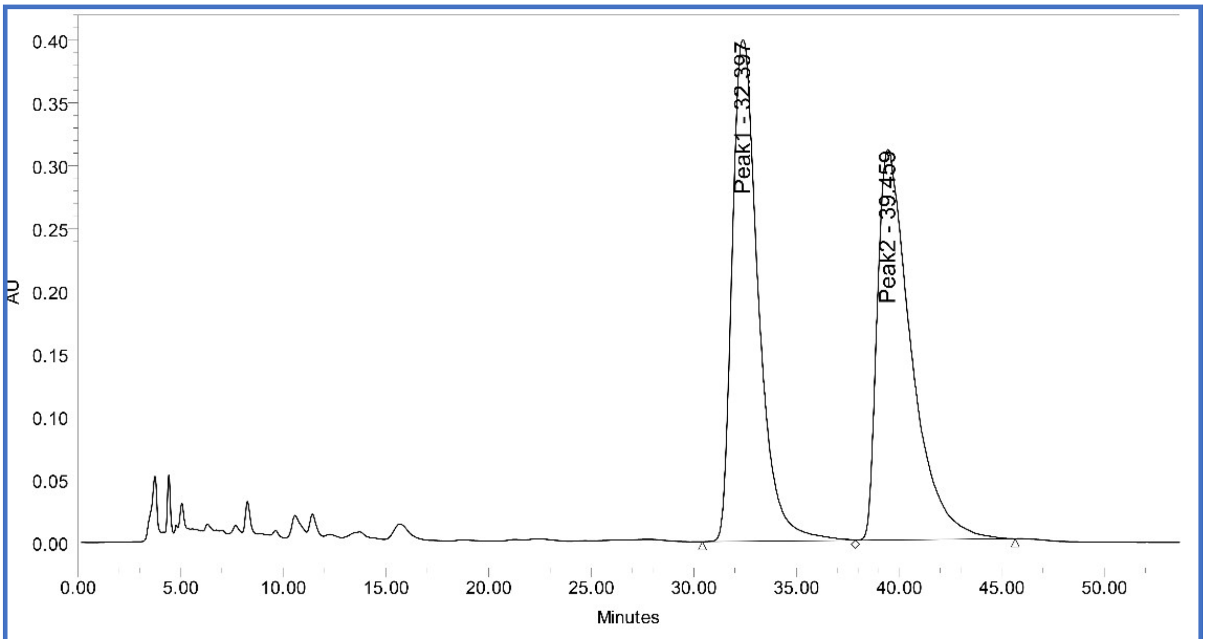

	Peak Name	RT (min)	Area (µV*sec)	% Area	Height (µV)	% Height
1	Peak1	32.397	35458724	50.00	399111	56.22
2	Peak2	39.459	35461021	50.00	310815	43.78

Figure S42: HPLC data of enantioenriched and racemic of **4g.**

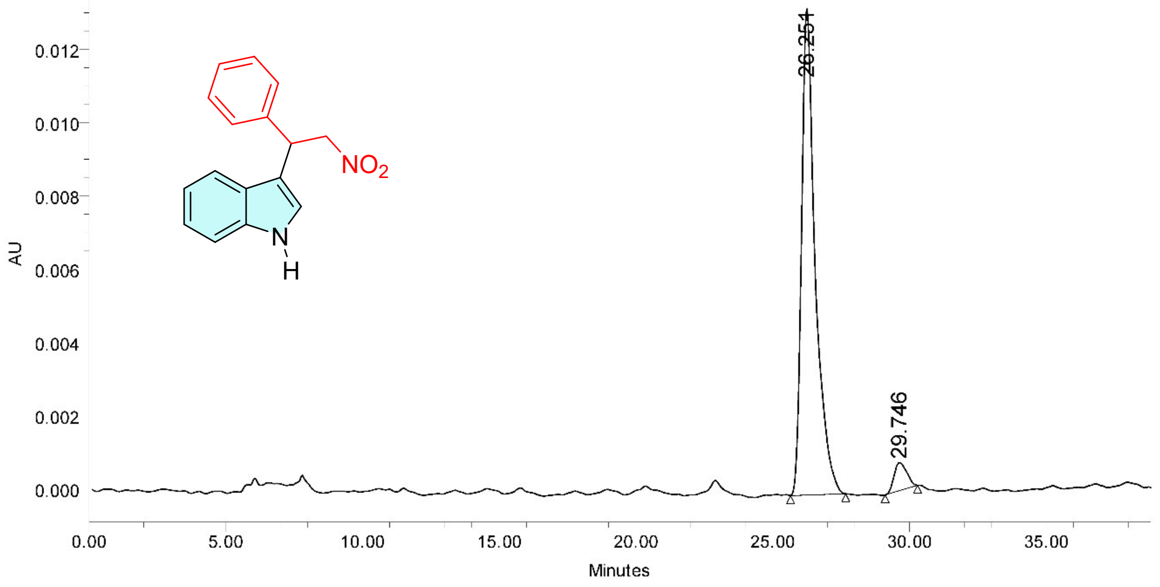

	RT (min)	Area (µV*sec)	% Area	Height (µV)	% Height
1	26.251	461241	94.97	13270	94.56
2	29.746	24408	5.03	764	5.44

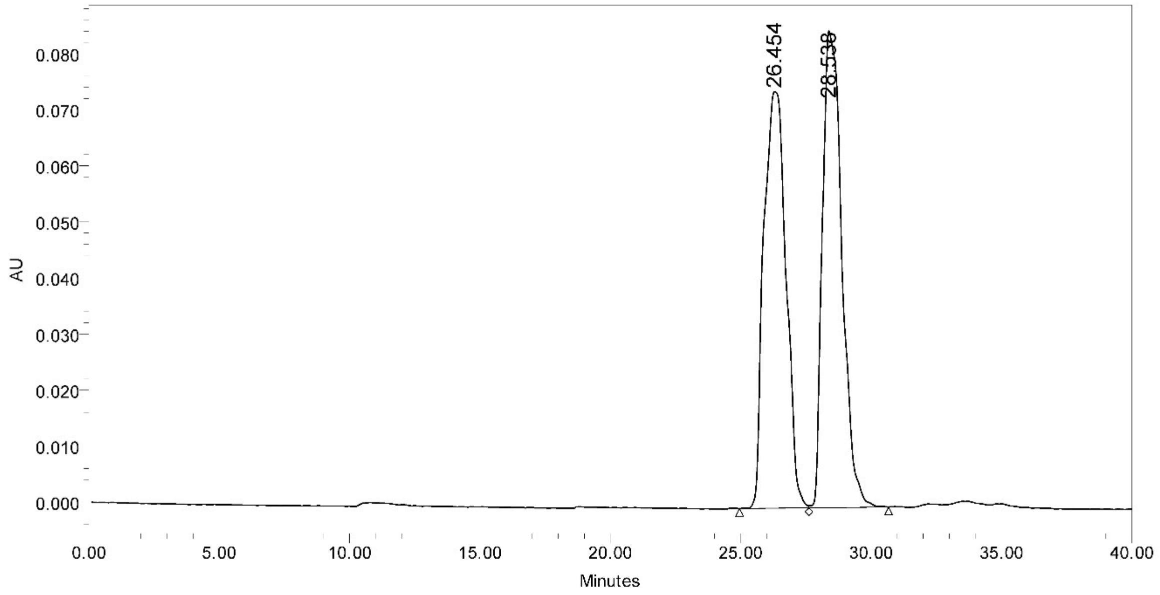

	RT (min)	Area (µV*sec)	% Area	Height (µV)	% Height
1	26.454	4406089	49.90	74313	46.63
2	28.538	4423115	50.10	85065	53.37

Figure S43: HPLC data of enantioenriched and racemic of **4h.**

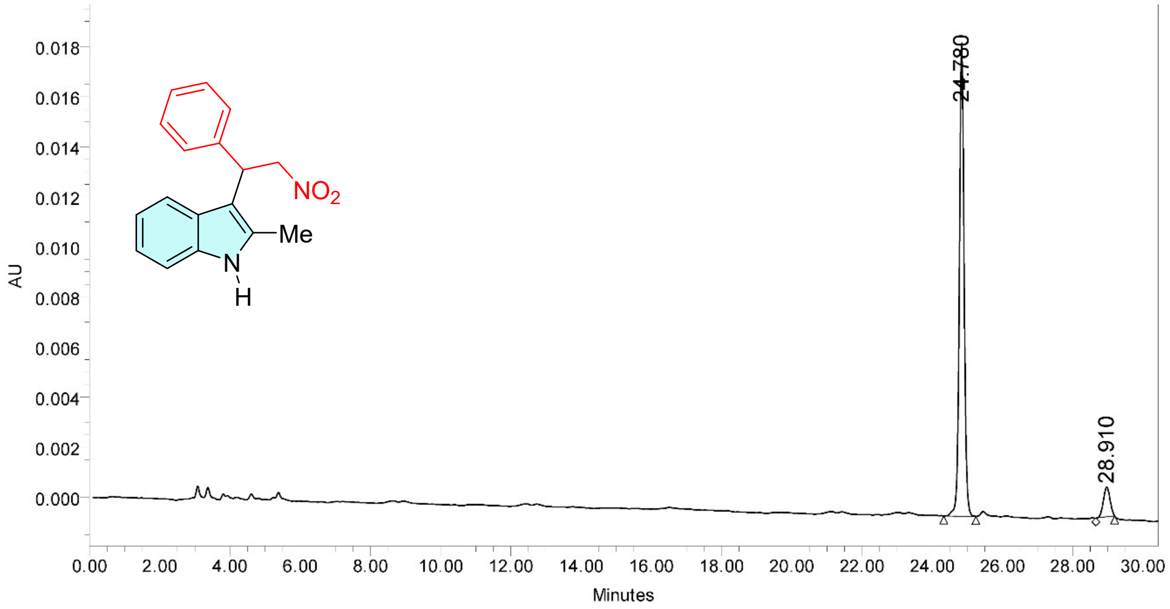

	RT (min)	Area (µV*sec)	% Area	Height (µV)	% Height
1	24.780	178799	92.02	18880	94.10
2	28.910	15508	7.98	1185	5.90

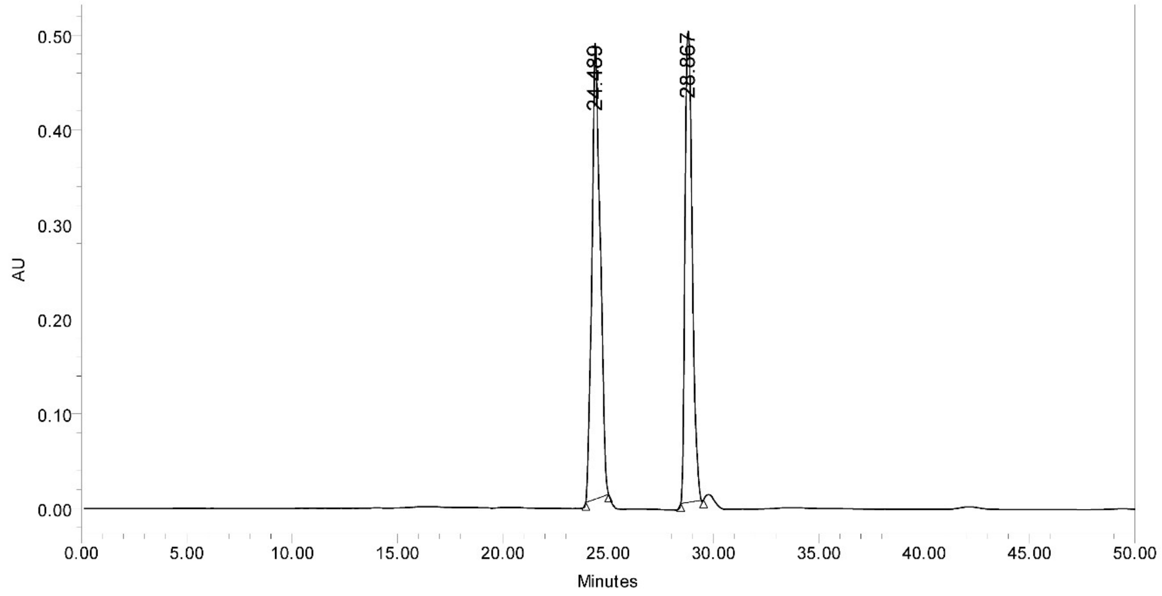

	RT (min)	Area (µV*sec)	% Area	Height (µV)	% Height
1	24.489	13078264	52.47	481589	49.17
2	28.867	11847213	47.53	497766	50.83

Figure S44: HPLC data of enantioenriched and racemic of **4i**.

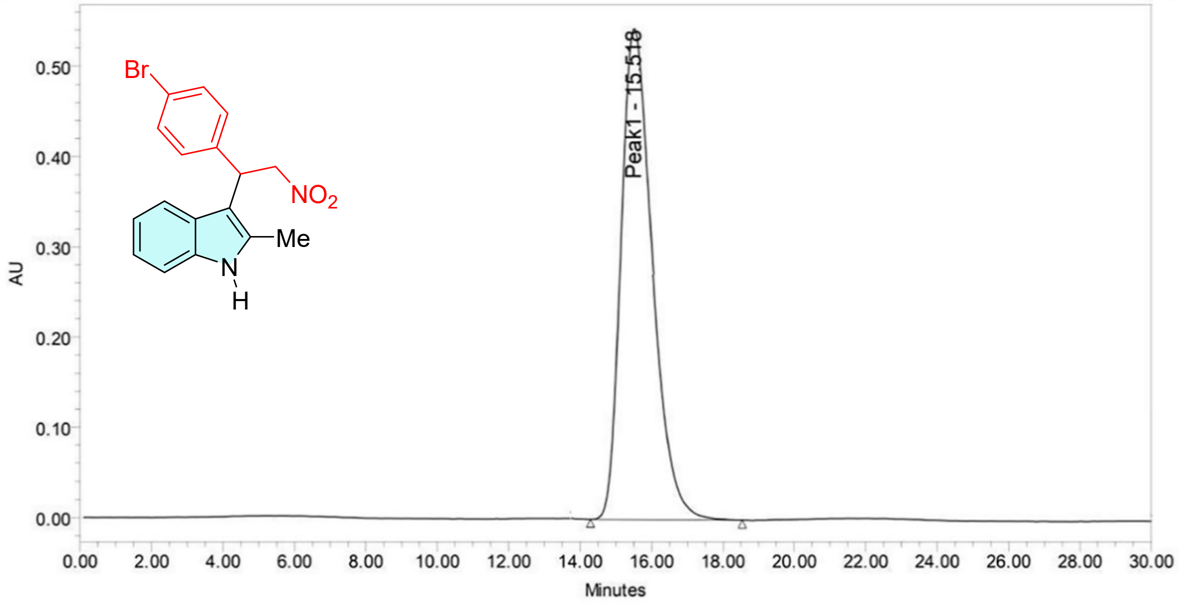

	Peak Name	RT (min)	Area (µV*sec)	% Area	Height (µV)	% Height
1	Peak1	15.518	32755555	100.00	537150	100.00

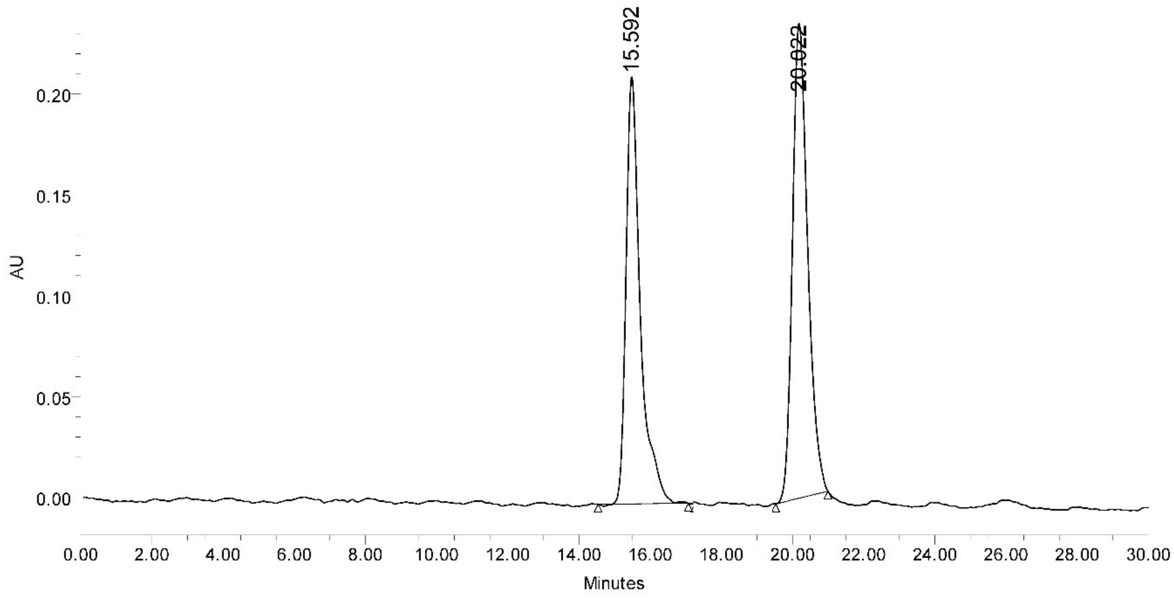

	RT (min)	Area (µV*sec)	% Area	Height (µV)	% Height
1	15.592	5950921	45.94	211760	47.35
2	20.022	7002855	54.06	235478	52.65

Figure S45: HPLC data of enantioenriched and racemic of **4j**.

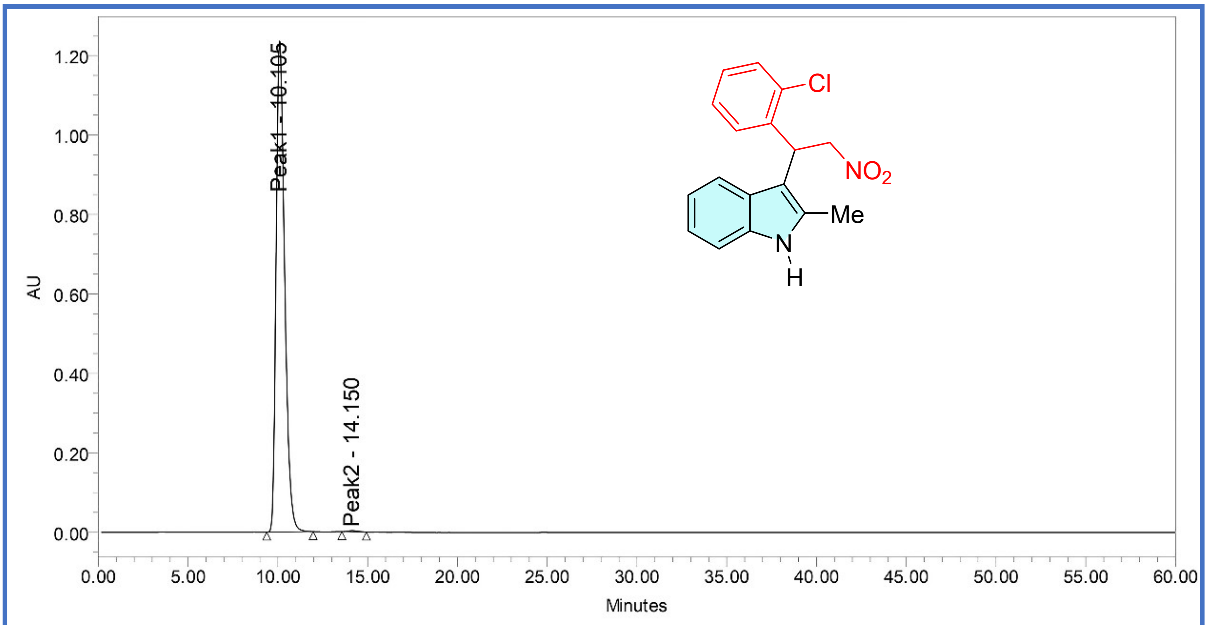

	Peak Name	RT (min)	Area (µV*sec)	% Area	Height (µV)	% Height
1	Peak1	10.105	40801028	99.74	1235399	99.78
2	Peak2	14.150	106828	0.26	2774	0.22

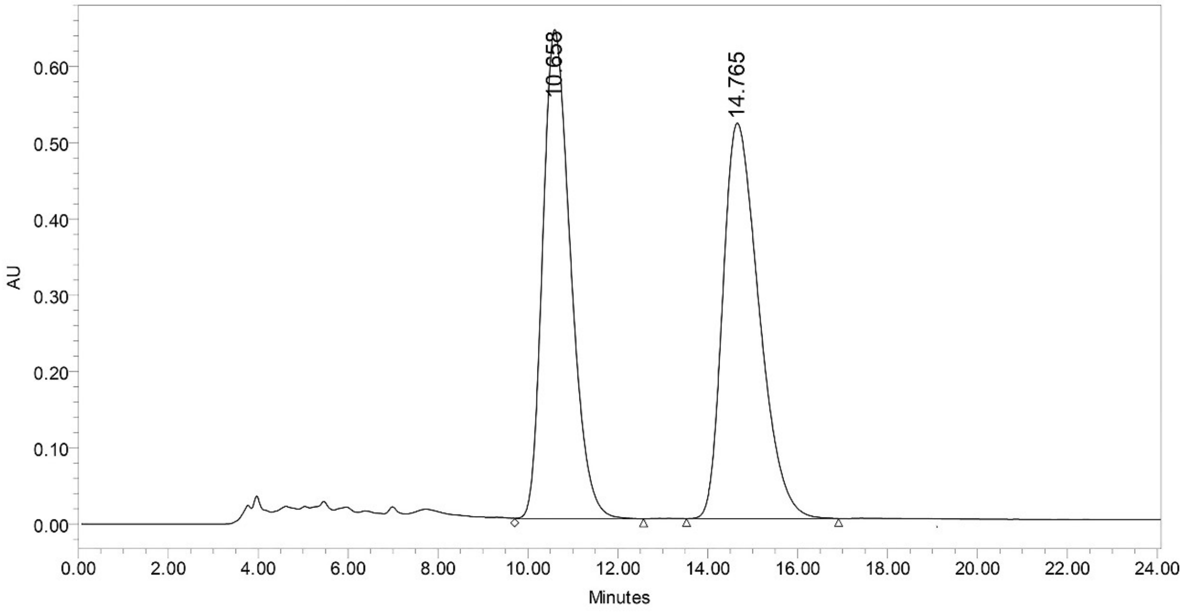

	RT (min)	Area (µV*sec)	% Area	Height (µV)	% Height
1	10.658	27618100	48.80	641646	55.30
2	14.765	28981732	51.20	518669	44.70

Figure S46: HPLC data of enantioenriched and racemic of **4k**.

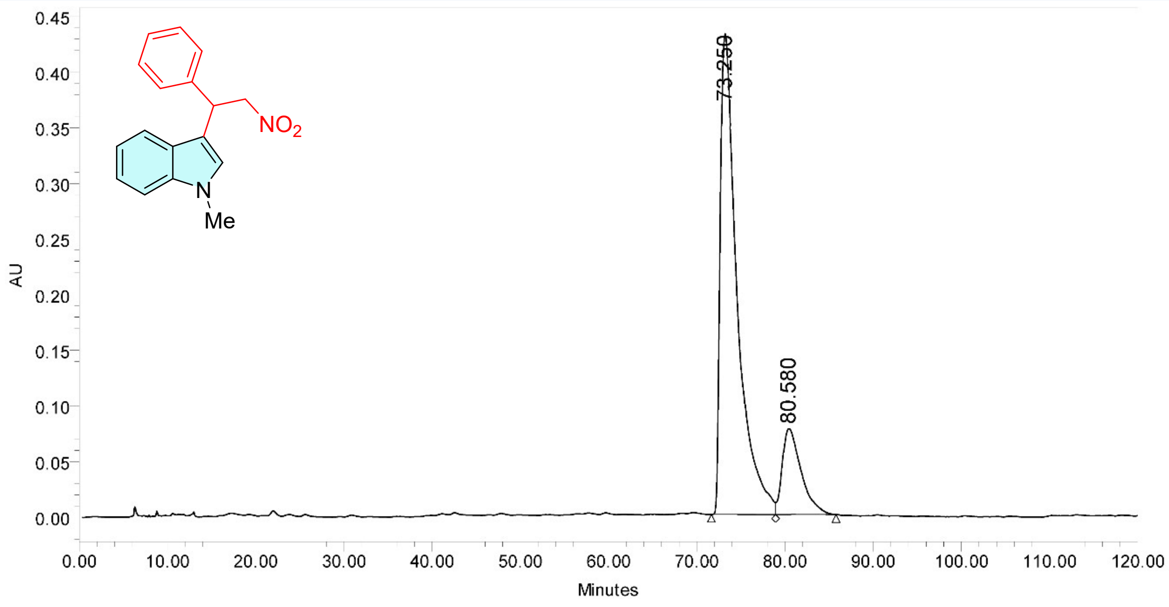

	RT (min)	Area (μV*sec)	% Area	Height (μV)	% Height
1	73.250	53980578	82.72	431509	84.86
2	80.580	11278880	17.28	76984	15.14

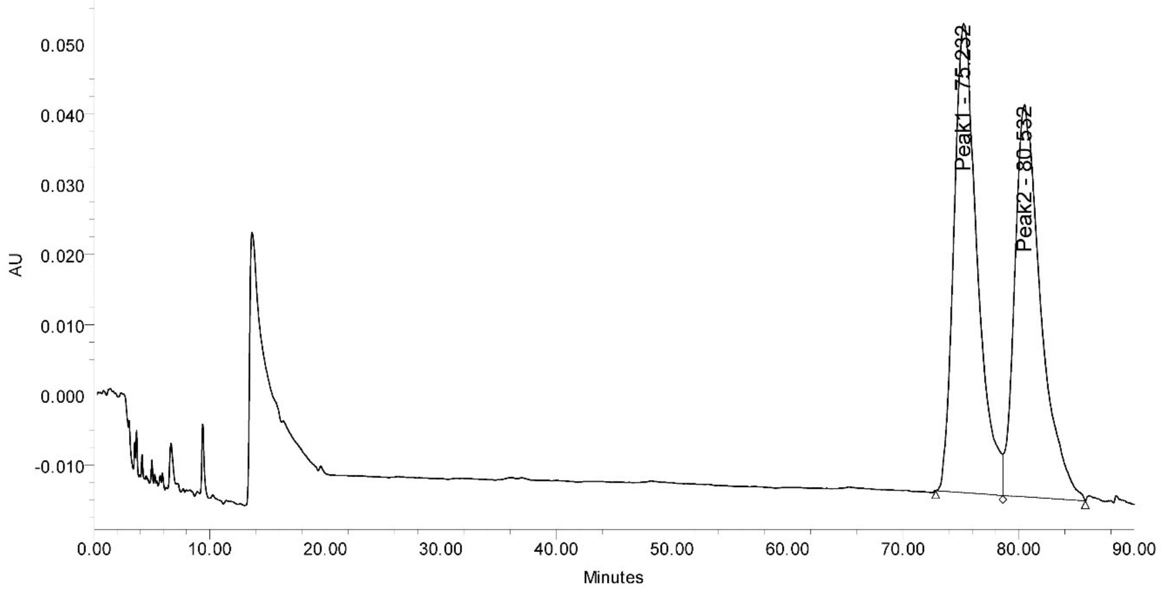

	Peak Name	RT (min)	Area (μV*sec)	% Area	Height (μV)	% Height
1	Peak1	75.232	1843665	50.93	13356	54.47
2	Peak2	80.532	1776247	49.07	11164	45.53

Figure S47: HPLC data of enantioenriched and racemic of **4l**.

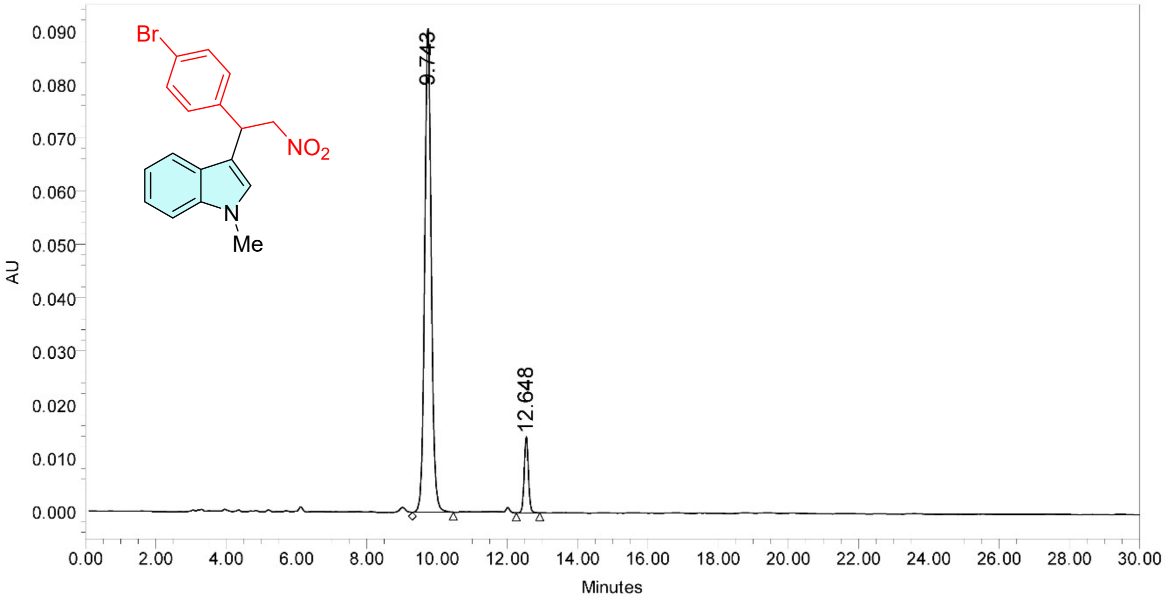

	RT (min)	Area (μV*sec)	% Area	Height (μV)	% Height
1	9.743	1180749	90.97	90858	86.44
2	12.648	117136	9.03	14248	13.56

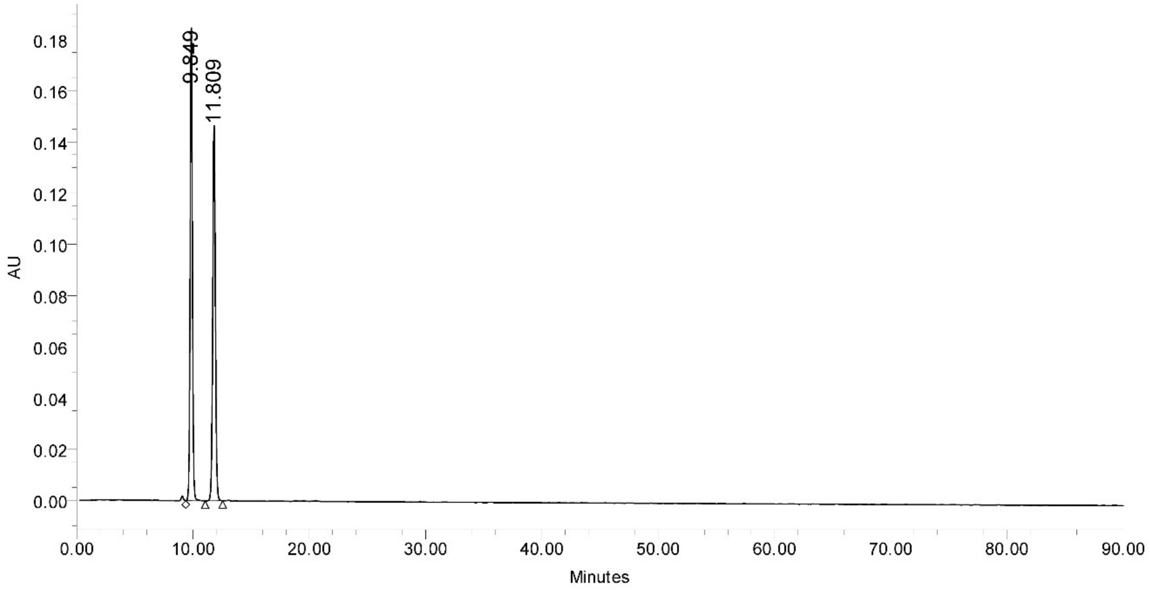

	RT (min)	Area (μV*sec)	% Area	Height (μV)	% Height
1	9.849	2444715	51.26	184652	55.67
2	11.809	2324442	48.74	147017	44.33

Figure S48: HPLC data of enantioenriched and racemic of **4m**.

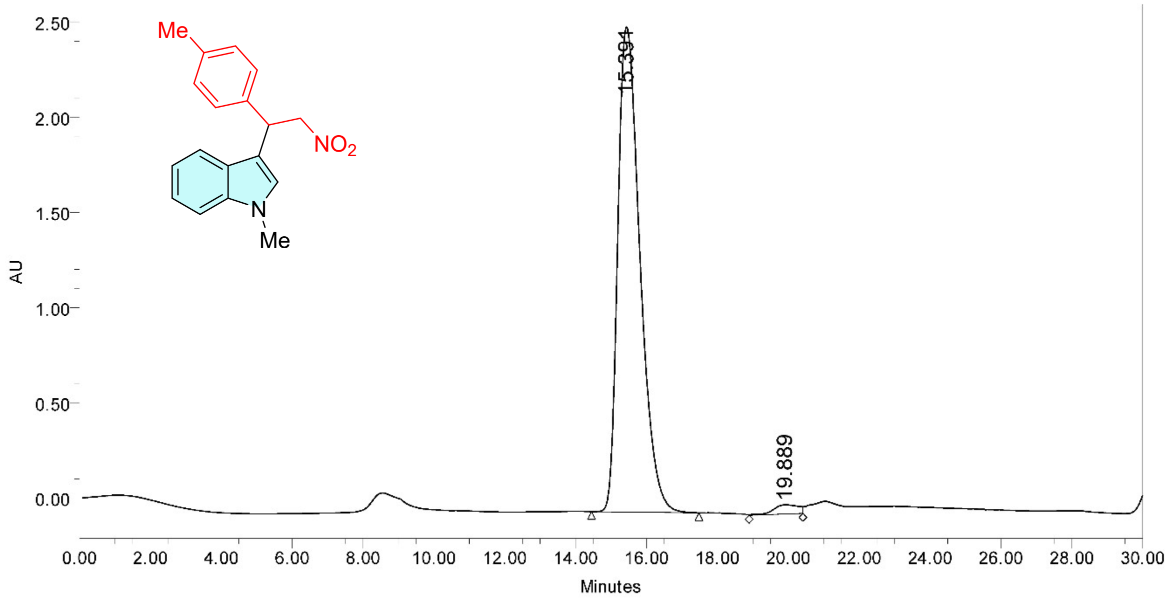

	RT (min)	Area (µV*sec)	% Area	Height (µV)	% Height
1	15.391	110475703	97.89	2547674	98.14
2	19.889	2385400	2.11	48410	1.86

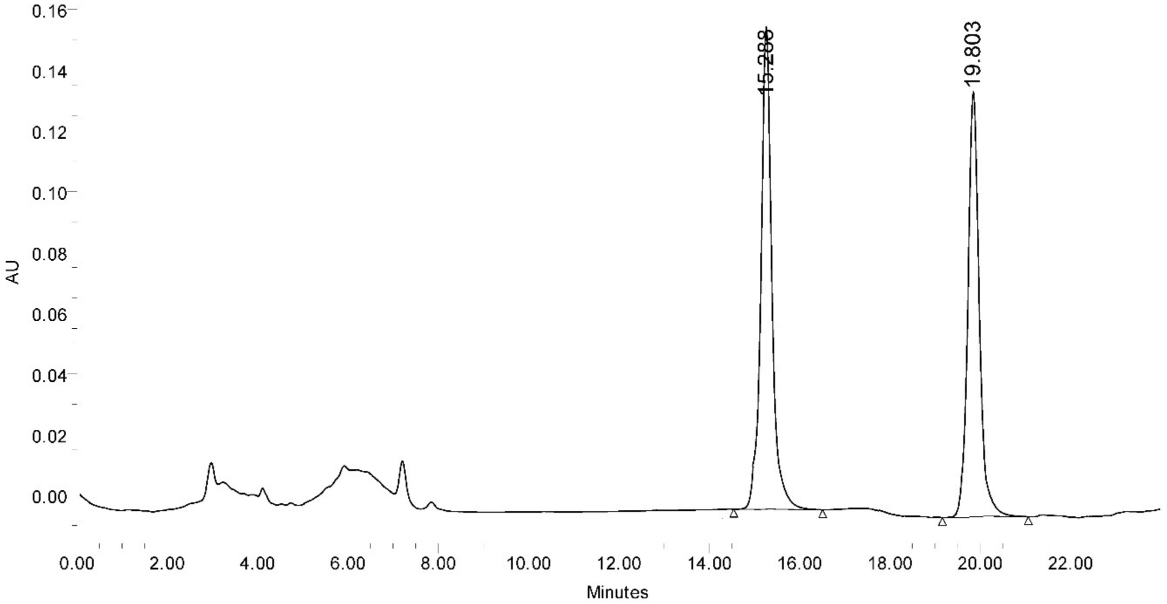

	RT (min)	Area (µV*sec)	% Area	Height (µV)	% Height
1	15.288	2840572	51.50	159035	53.18
2	19.803	2675167	48.50	140011	46.82

Figure S49: HPLC data of enantioenriched and racemic of **4n**.

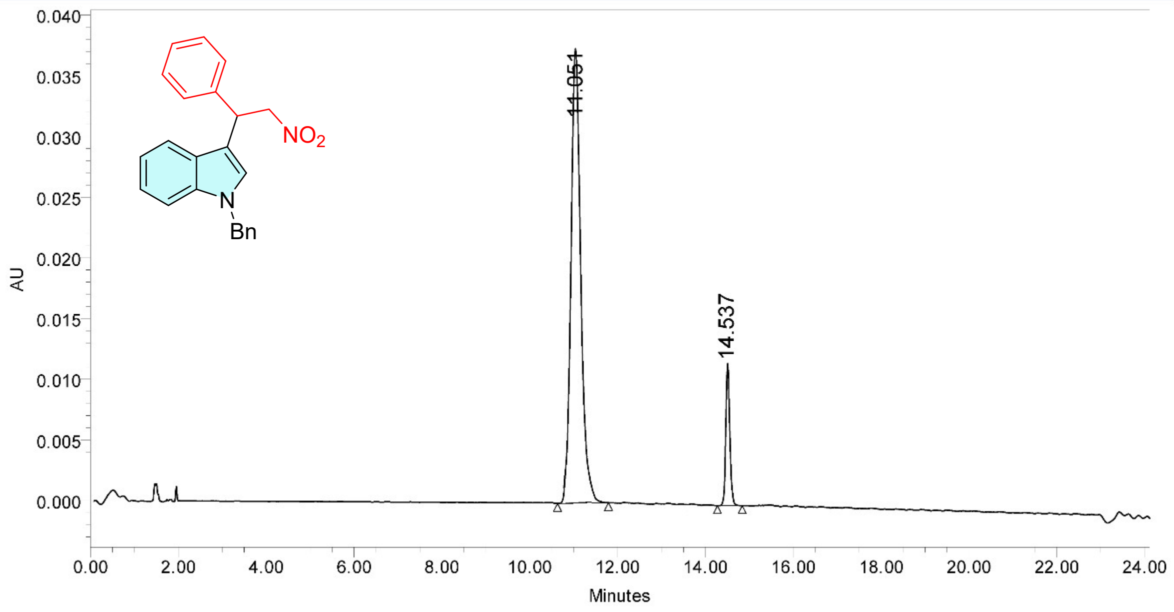

	RT (min)	Area (µV*sec)	% Area	Height (µV)	% Height
1	11.051	533100	87.63	36250	75.74
2	14.537	75223	12.37	11610	24.26

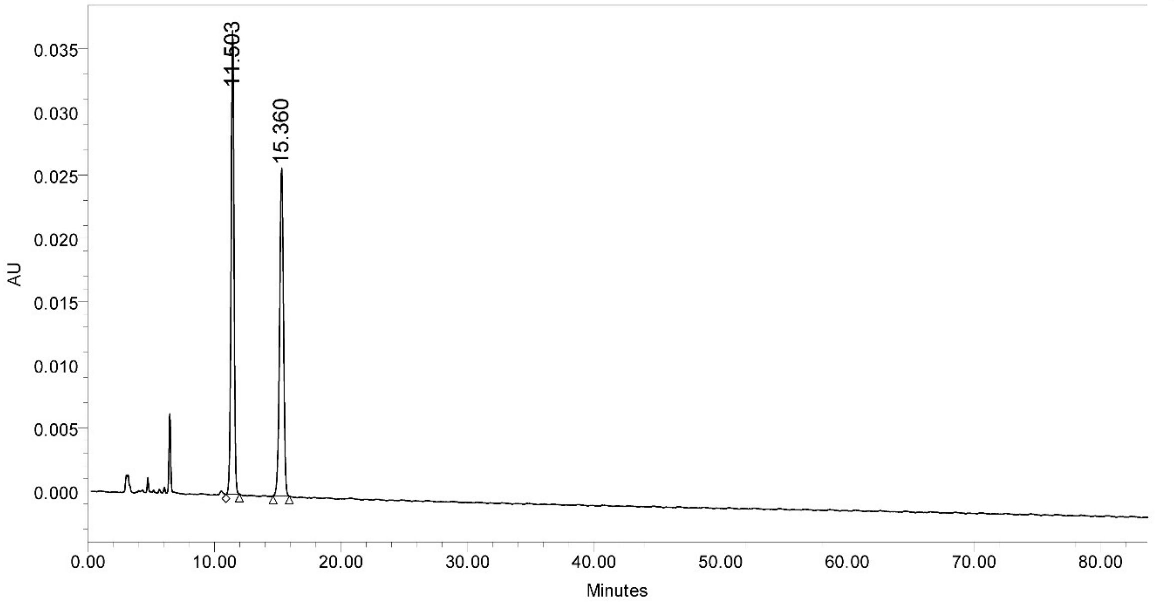

	RT (min)	Area (µV*sec)	% Area	Height (µV)	% Height
1	11.503	581884	51.16	36660	58.54
2	15.360	555453	48.84	25961	41.46

Figure S50: HPLC data of enantioenriched and racemic of **4o**

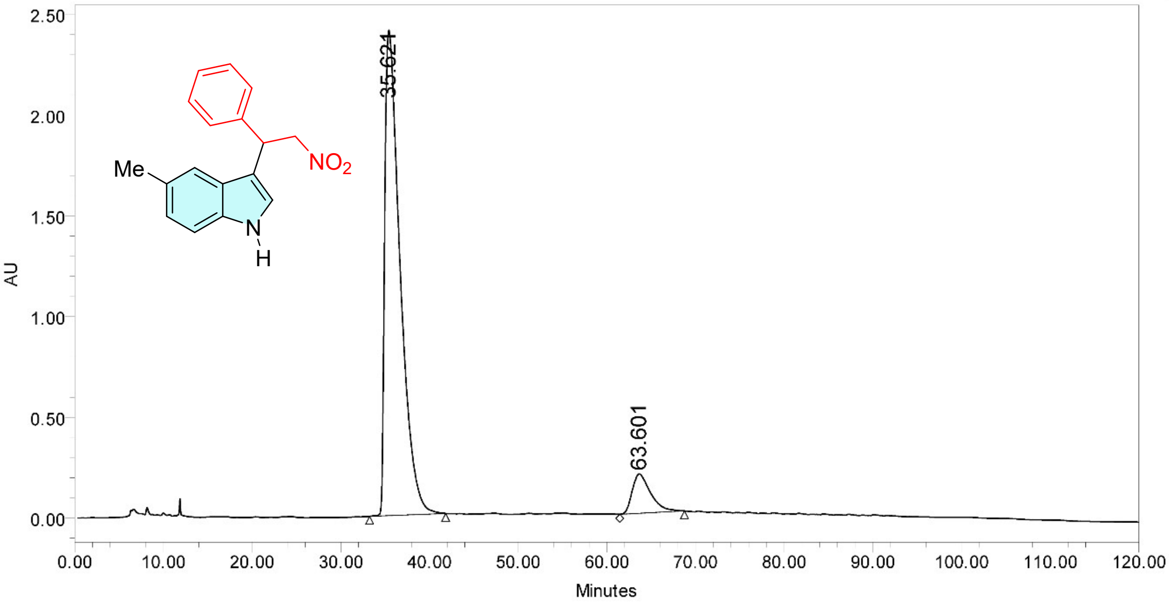

	RT (min)	Area (µV*sec)	% Area	Height (µV)	% Height
1	35.621	289669374	91.08	2410348	92.51
2	63.601	28366707	8.92	195209	7.49

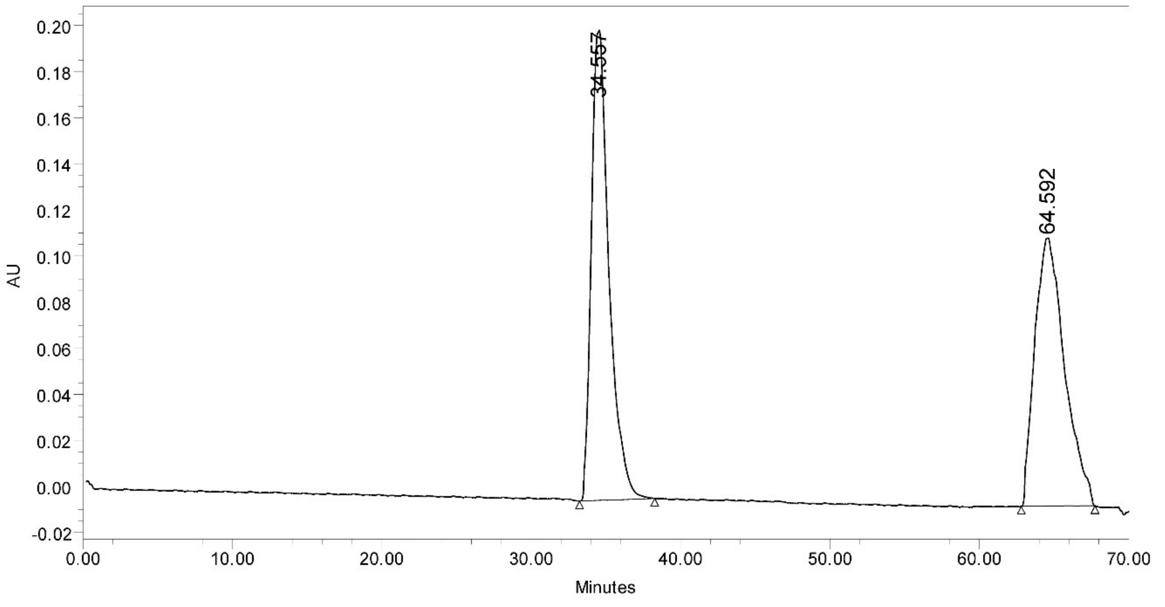

	RT (min)	Area (µV*sec)	% Area	Height (µV)	% Height
1	34.557	1617301	51.20	20167	64.22
2	64.592	1541726	48.80	11234	35.78

Figure S51: HPLC data of enantioenriched and racemic of **5a.**

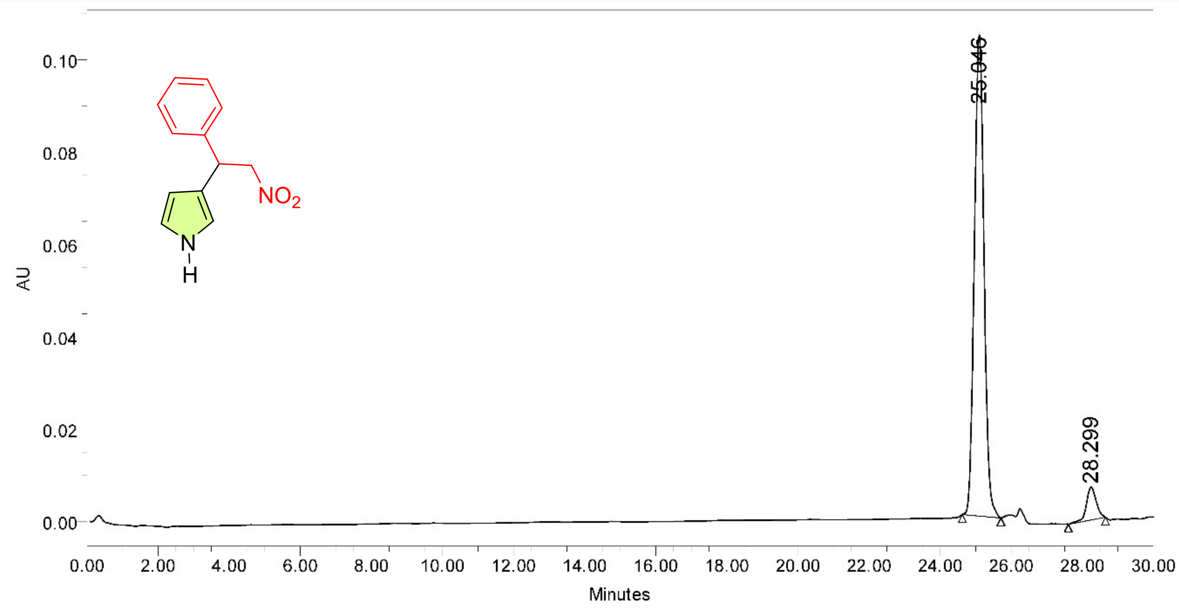

	RT (min)	Area (µV*sec)	% Area	Height (µV)	% Height
1	25.046	1965414	93.46	104151	93.59
2	28.299	137423	6.54	7133	6.41

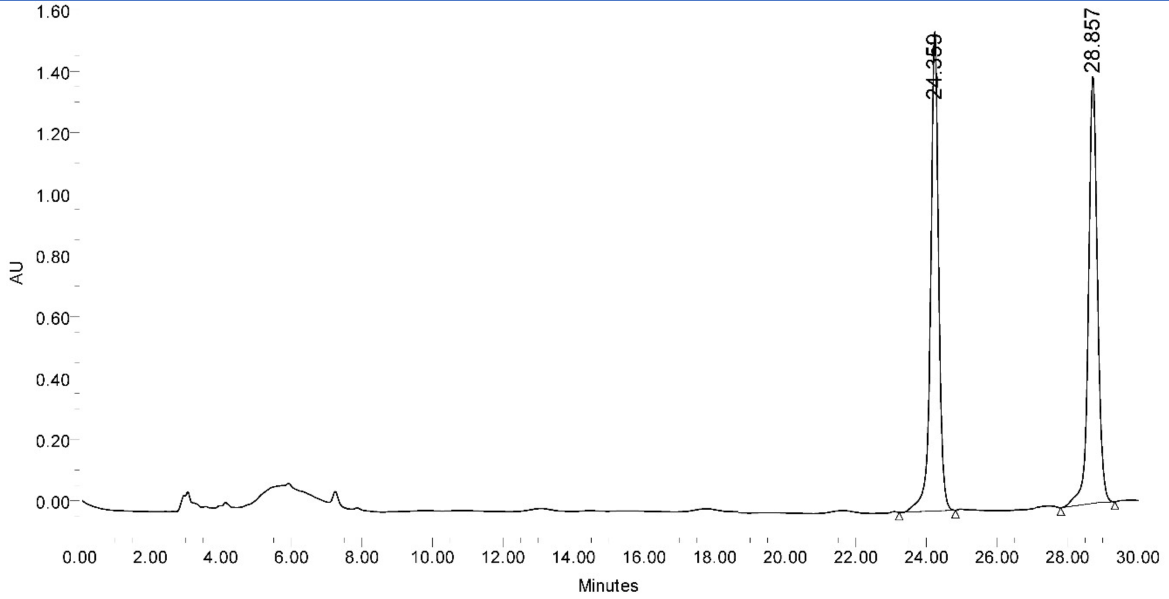

	RT (min)	Area (µV*sec)	% Area	Height (µV)	% Height
1	24.359	24726753	49.83	1565047	52.90
2	28.857	24897252	50.17	1393325	47.10

Figure S52: HPLC data of enantioenriched and racemic of **5b.**

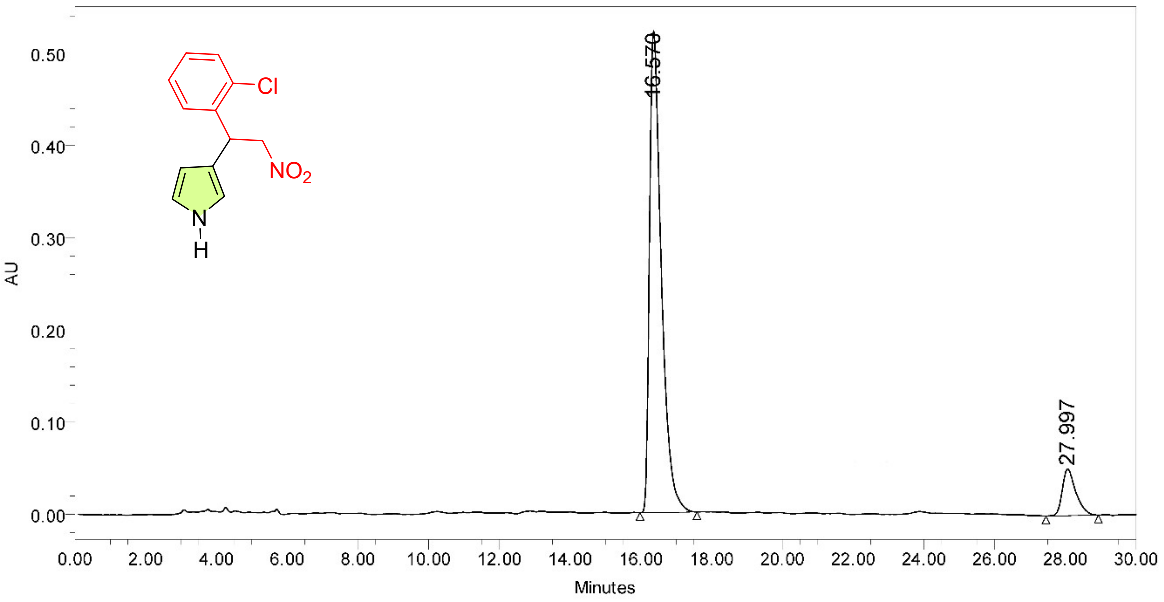

	RT (min)	Area (µV*sec)	% Area	Height (µV)	% Height
1	16.570	12125764	89.97	522551	91.17
2	27.997	1351939	10.03	50634	8.83

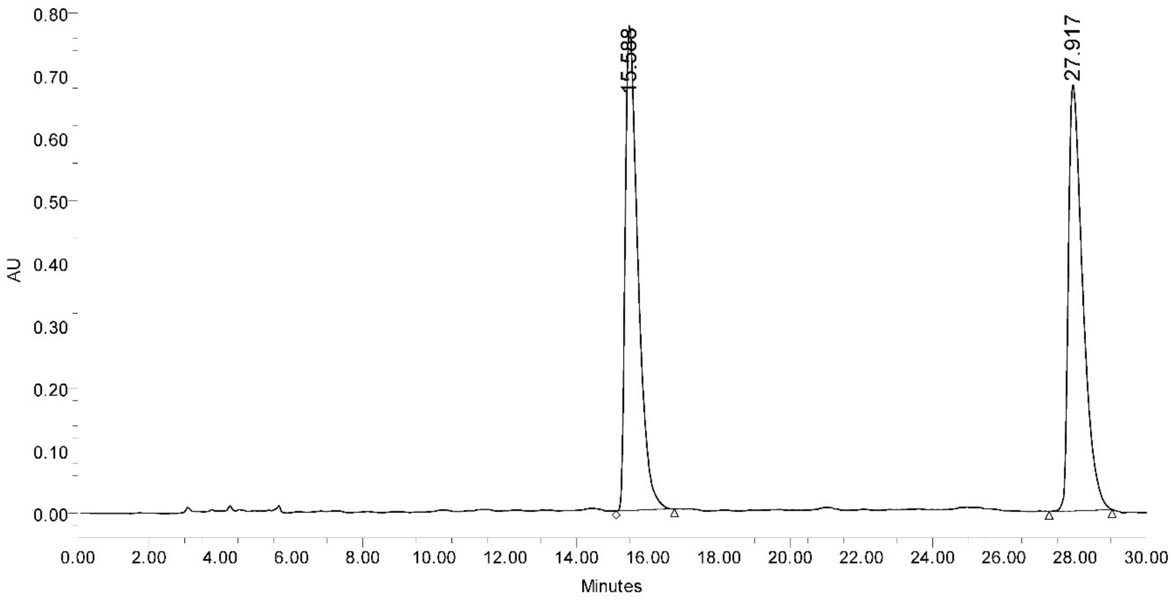

	RT (min)	Area (µV*sec)	% Area	Height (µV)	% Height
1	15.588	19276143	50.00	775829	53.22
2	27.917	19272901	50.00	682069	46.78

Figure S53: HPLC data of enantioenriched and racemic of **5c.**

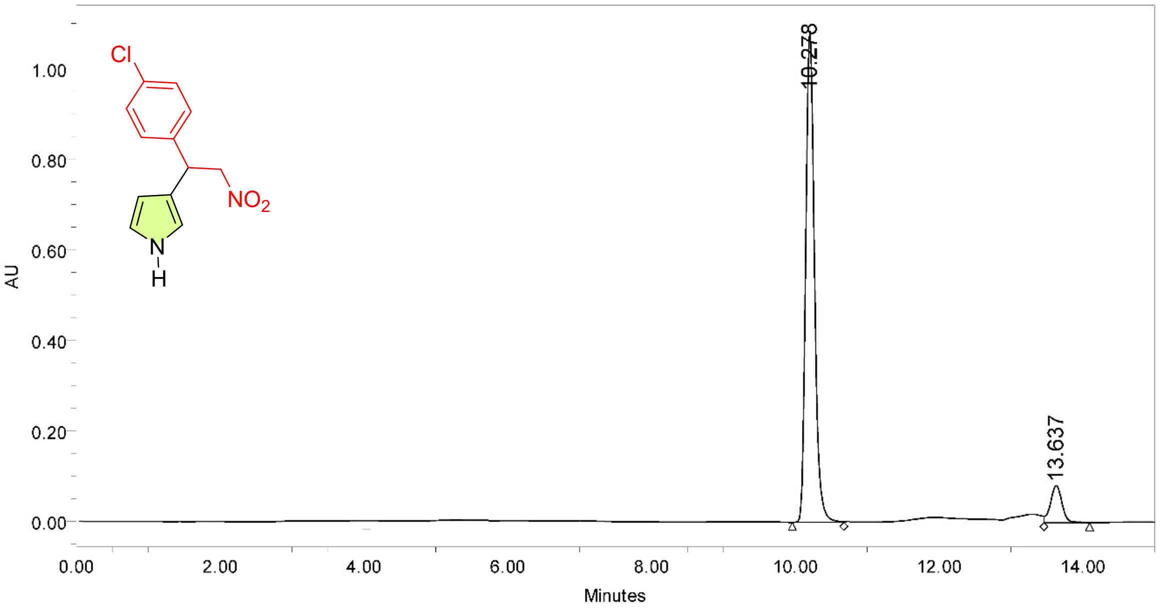

	RT (min)	Area (µV*sec)	% Area	Height (µV)	% Height
1	10.278	9136904	90.91	1087249	92.97
2	13.637	914120	9.09	82157	7.03

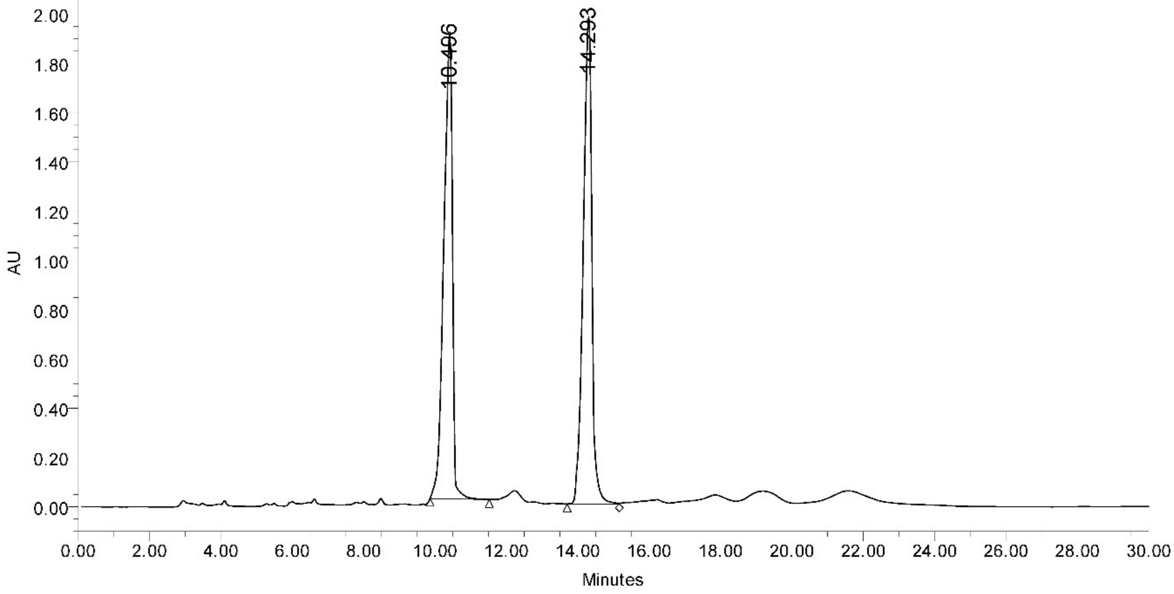

	RT (min)	Area (µV*sec)	% Area	Height (µV)	% Height
1	10.496	31370386	50.35	1900825	49.47
2	14.293	30929574	49.65	1941308	50.53

Figure S54: HPLC data of enantioenriched and racemic of **5d.**

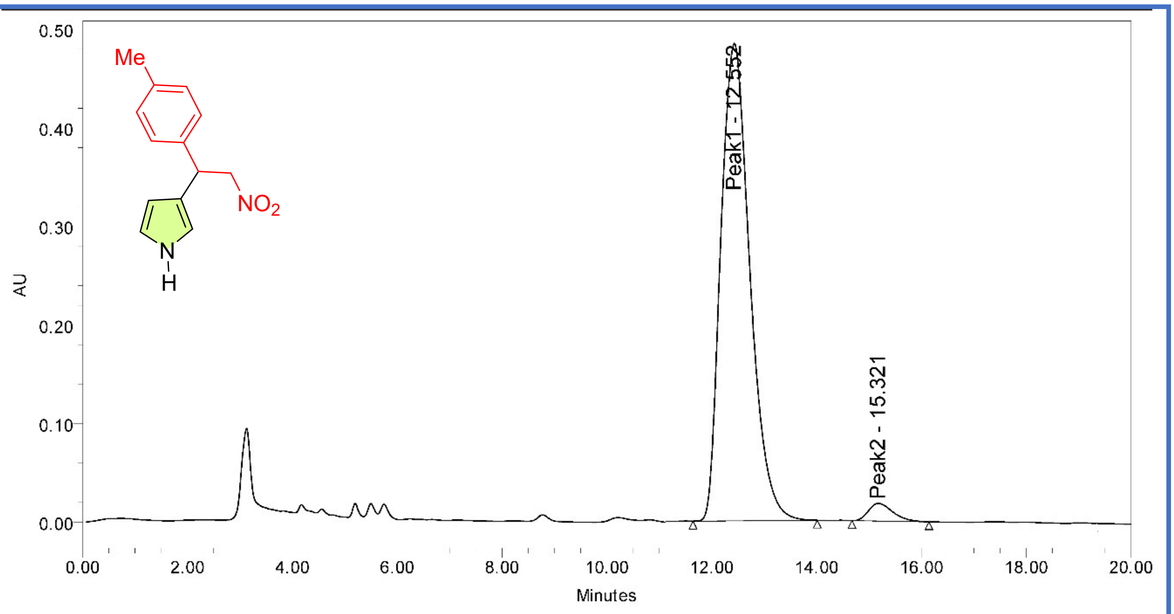

	Peak Name	RT (min)	Area (µV*sec)	% Area	Height (µV)	% Height
1	Peak1	12.552	18609941	96.97	484394	96.41
2	Peak2	15.321	580749	3.03	18061	3.59

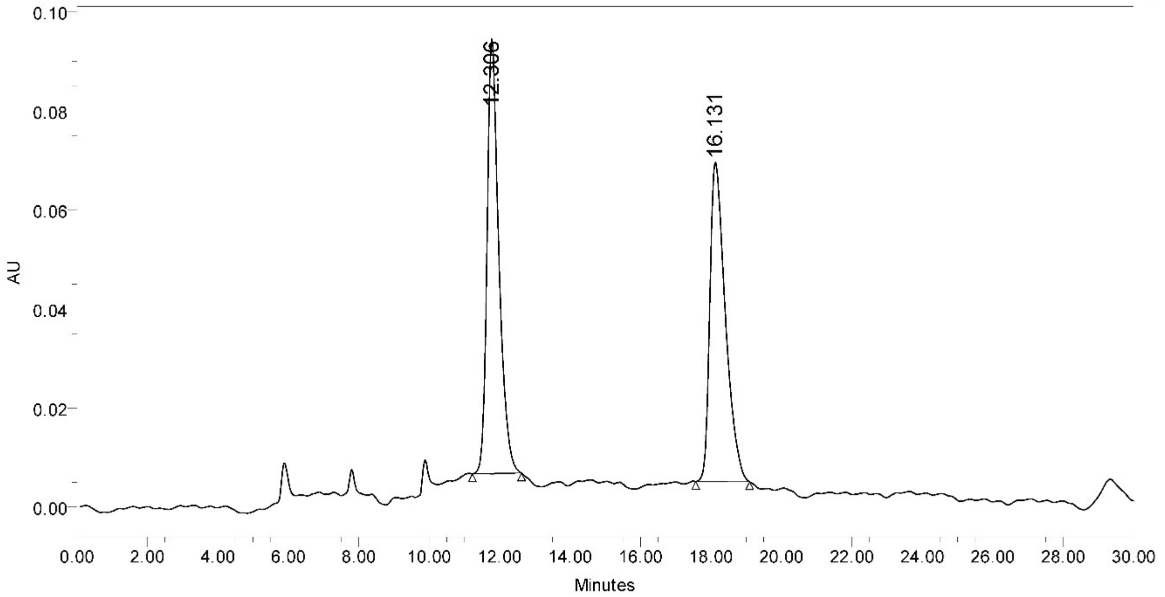

	RT (min)	Area (µV*sec)	% Area	Height (µV)	% Height
1	12.306	2046535	50.84	87759	57.64
2	16.131	1978512	49.16	64500	42.36

1. Lalhmangaihzuala S, Laldinpuii Z, Lalthanpuii PB, et al (2024) Screening of novel

carbohydrate-derived thioureas for antibacterial activity. J Appl Pharm Sci 14,:094–102. https://doi.org/10.7324/JAPS.2024.180485

2. Vanlaldinpuia K, Bora P, Bez G (2017) Monofunctional primary amine: A new class of organocatalyst for asymmetric Aldol reaction. J Chem Sci 2017 1293 129:301–312. https://doi.org/10.1007/S12039-017-1237-Y

3. Vanlaldinpuia K, Bez G (2011) Useful methods for the synthesis of isopropylidenes and their chemoselective cleavage. Tetrahedron Lett 52:3759–3764. https://doi.org/10.1016/J.TETLET.2011.05.050

4. Tu Y, Wang Z, Frohn M, et al (1998) Structural Probing of Ketone Catalysts for Asymmetric Epoxidation which show encouraging potential for asymmetric syn-. J Org Chem 63:8475–8485
